# *In-situ* detection based on the biofilm hydrophilicity for environmental biofilm formation

**DOI:** 10.1038/s41598-019-44167-6

**Published:** 2019-05-30

**Authors:** Nobuyuki Tanaka, Takeshi Kogo, Nobumitsu Hirai, Akiko Ogawa, Hideyuki Kanematsu, Junko Takahara, Akane Awazu, Nobuko Fujita, Yoshihide Haruzono, Shunji Ichida, Yo Tanaka

**Affiliations:** 1 RIKEN Center for Biosystems Dynamics Research, 1-3 Yamadaoka, Suita, Osaka 565-0871 Japan; 2 National Institute of Technology (KOSEN), Suzuka College, Shiroko-cho, Suzuka, Mie 510-0294 Japan; 3 Kitagawa Corporation, 77-1 Motomachi, Fuchu-shi, Hiroshima 726-8610 Japan

**Keywords:** Wetting, Characterization and analytical techniques

## Abstract

A biofilm has a unique structure composed of microorganisms, extracellular polymeric substances (EPSs), etc., and it is layered on a substrate in water. In material science, it is important to detect the biofilm formed on a surface to prevent biofouling. EPSs, the major component of the biofilm, mainly consist of polysaccharides, proteins, nucleic acids, and lipids. Because these biomolecules have a variety of hydrophilicities or hydrophobicities, the substrate covered with the biofilm shows different wettability from the initial state. To detect the biofilm formation, this study employed a liquid-squeezing-based wettability assessment method with a simple wettability index: the liquid-squeezed diameter of a smaller value indicates higher wettability. The method is based on the liquid-squeezing behaviour of a liquid that covers sample surfaces when an air-jet is applied. To form the biofilm, polystyrene surfaces were immersed and incubated in a water-circulated bioreactor that had collected microorganisms in ambient air. After the 14-d incubation, good formation of the biofilm on the surfaces was confirmed by staining with crystal violet. Although the contact angles of captive bubbles on the surfaces with the biofilm were unmeasurable, the liquid-squeezing method could distinguish between hydrophilic and hydrophobic initial surfaces with and without biofilm formation using the diameter of the liquid-squeezed area. The surface wettability is expected to be a promising property for *in-situ* detection of biofilm formation on a macroscopic scale.

## Introduction

Commonly, a biofilm is defined as an aggregate of microorganisms in which cells that are frequently embedded within a self-produced matrix of extracellular polymeric substances (EPSs) adhere to each other and/or to a surface. A biofilm is a system that can be adapted internally to environmental conditions by its inhabitants. The self-produced matrix of EPSs, which is also referred to as slime, is a polymeric conglomeration generally composed of extracellular biopolymers in various structural forms^1^. The biofilm formation is a phased process: (1) substances dissolved in water are physically adsorbed onto a surface, and this is referred to as a conditioning film; (2) next, microorganisms adhere onto the conditioning film as a scaffold; (3) the microorganisms secrete EPSs embedding the microorganisms themselves, and this results in a microorganism colony and biofilm; and (4) some of the microorganisms are detached from the biofilm and dispersed into the surrounding environment^2,3^. In the biofilm, microorganisms adapt to their surrounding area and easily grow; therefore, the biofilm affects the surrounding environmental and sanitary conditions^4,5^. Furthermore, because the biofilm has electrochemical properties, a substrate on which the biofilm has formed (referred to as a biofilm-formed substrate) is more easily corroded as compared with a biofilm-free substrate^6,7^. Detection of the biofilm is normally confirmed by the presence of EPSs, in particular, staining of EPSs is a common approach^8–10^. This study focused on the EPSs that consist of various types of substances; that is, polysaccharides, proteins, nucleic acids, and lipids, which show a variety of hydrophilicities or hydrophobicities^11^. Through biofilm formation, the surface covered with the biofilm is assumed to be hydrophilic; as a result, the wettability assessment becomes a method for detecting biofilm formation *in-situ*.

Wettability is a surface property that shows affinity of the surface to a contact liquid^12^. This property can be visualized by the behaviour of a liquid droplet on the surface; liquid droplets spread on a high wettability surface and are repelled on a low wettability one. The contact angle is a standard index to quantify the surface wettability in various scientific fields^12–16^. As an in-liquid measurement, the captive bubble method has been used for determining the contact angle of a bubble attaching on a cell surface in liquid^12,17–19^. However, the captive bubble method is unsuitable for some high wettability cases and it is difficult to use in an upright position where the sample surface faces upwards. Recently, a method for assessing surface wettability based on the behaviour of a liquid squeezed by an air-jet has been proposed^20,21^. In this method, the diameter of the liquid squeezed area during air-jet application can be used as an index of surface wettability with a high correlation to contact angle^21^. Furthermore, this index is measurable even *in situ*. Through the wettability assessment by liquid squeezing, this study demonstrated that the surface wettability is a useful property for *in-situ* detection of biofilm formation on a macroscopic scale.

## Results

### Biofilm formation

**Figure 1:**
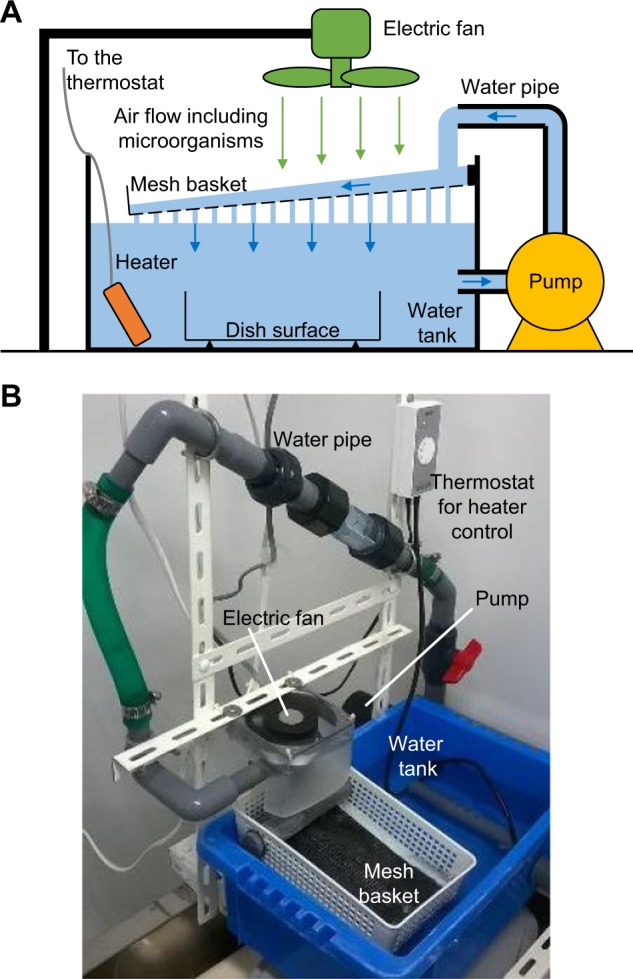
Bioreactor for biofilm formation by microorganisms in ambient air. Schematic diagram (**A**) shows the setup of the bioreactor with a water circulation system and an electric fan for collecting and introducing ambient air flow including microorganisms into the water. Photograph (**B**) shows the actual experimental setup.

For the biofilm formation, two types of sample dishes, (1) polystyrene (PS) and (2) vacuum gas plasma-treated PS (VGP-PS) dishes were prepared and placed in the water tank of the bioreactor with water circulation at 27 °C (Fig. 1A). Because the bioreactor was located in an ordinary laboratory room, microorganisms in the ambient air were collected and introduced into the water in the tank by using an electric fan.

**Figure 2:**
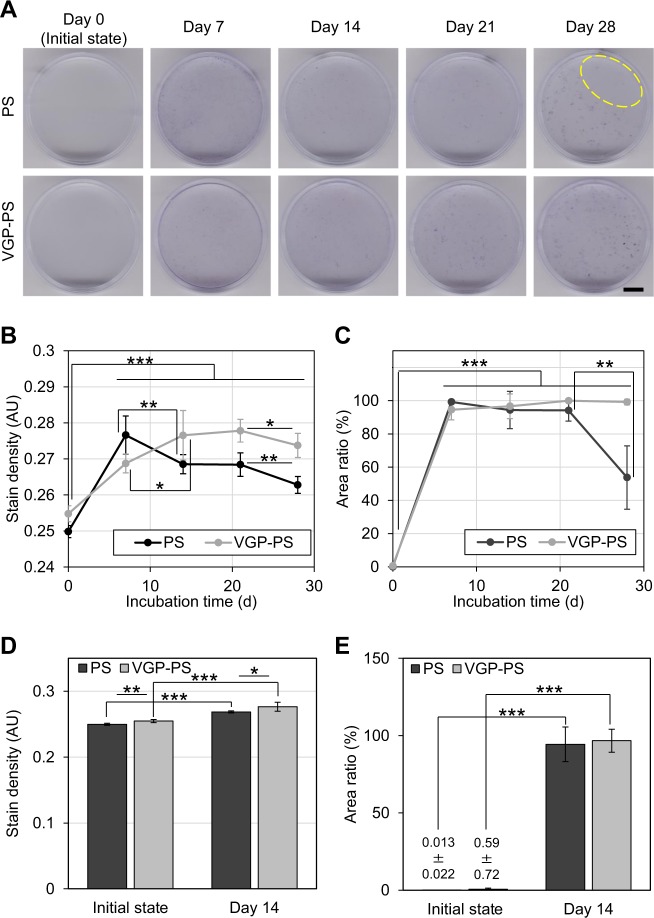
Biofilm formation in the bioreactor. Photographs (**A**) show crystal violet stained surfaces with respect to the incubation time of 0 (initial state), 7, 14, 21, and 28 d. PS, polystyrene; VGP, vacuum gas plasma-treated. The yellow dashed line surrounds the area showing biofilm detachment. The black bar indicates 1 cm. Graphs (**B**,**C**) show the time-series of average stain densities and percent ratios of stained area per unit area, respectively. Data points and error bars represent means and standard deviations, respectively (N = 5). Graphs (**D**,**E**) show the data at 0 and 14 d. Bars and error bars represent means and standard deviations, respectively (N = 5). Symbols: *p < 0.05; **p < 0.01; and ***p < 0.001 for each indicated combination. All original data are available in Supplementary Information 5.

To understand the biofilm formation in the bioreactor, the incubation for 0 (the initial state without incubation), 7, 14, 21, and 28 d and crystal violet staining on the dish after the incubation were performed (Fig. 2A). As a result, the stain densities on both the PS and VGP-PS dishes after the incubation were significantly higher than those before the incubation (p < 0.001) (Fig. 2B). The stain densities changed in the incubations from 7 d to 14 d and from 21 d to 28 d with statistical significances. By setting a threshold using the average density of dishes between the initial and incubated states, the percent ratio of stained area per unit area was determined (Fig. 2C). The area ratios in both PS and VGP-PS cases exceeded 90% at 7 d. Although the values of area ratios on the dishes with the incubation were significantly higher than those without the incubation, that on PS dish at 28 d significantly decreased to around 50% due to the partial detachment of biofilm (PS dish at Day 28 in Fig. 2A). As a result, the biofilms were speculated to cover almost the whole surface of the dish (over 90% total area) within the 7-d incubation and they were stably maintained between 14- and 21-d incubations. Based on these results, the incubation for biofilm detection was performed for 14 d. Therefore, four different conditions of sample dishes were prepared: (1) PS initial state; (2) PS with 14-d incubation; (3) VGP-PS initial state; and (4) VGP-PS with 14-d incubation. In the 14-d incubation, the stain densities had a statistically significant difference among the combinations of dish types or incubation times (Fig. 2D). On the other hand, the area ratios of stained area to unit area only had a statistically significant difference for the incubation times in each dish type (Fig. 2E).

**Figure 3:**
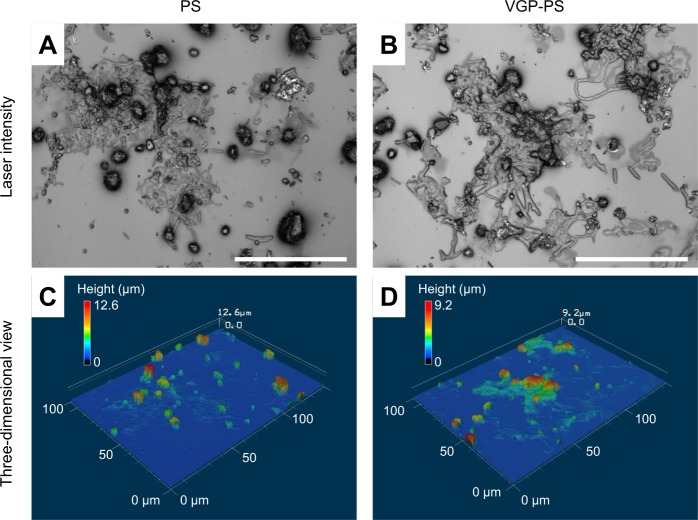
Confocal scanning laser microscopy on surfaces after 14-d incubation. Photographs (**A**,**B**) show the laser intensity on the PS and VGP-PS dishes, respectively. White bars indicate 50 μm. Graphs (**C**,**D**) show three-dimensional views of surfaces on the PS and VGP-PS dishes, respectively. The colour indicates the height with respect to the left-upper colour map in each graph.

From the confocal scanning laser microscopy on the surfaces of PS and VGP-PS dishes, small objects partly attached on the surfaces were observed (Fig. 3A,B). Some objects with the size of around 10 μm were found to have rod- and sphere-like shapes on both surfaces (Fig. 3A,B). As a result of the three-dimensional reconstruction of surface profiles (Fig. 3C,D), the maximum heights of objects on the PS and VGP-PS dishes were found to be around 12 and 9 μm, respectively. The individual objects with rod- or sphere-shapes had a height around 2 μm (Fig. 3C,D).

### Liquid squeezing by air-jet

**Figure 4:**
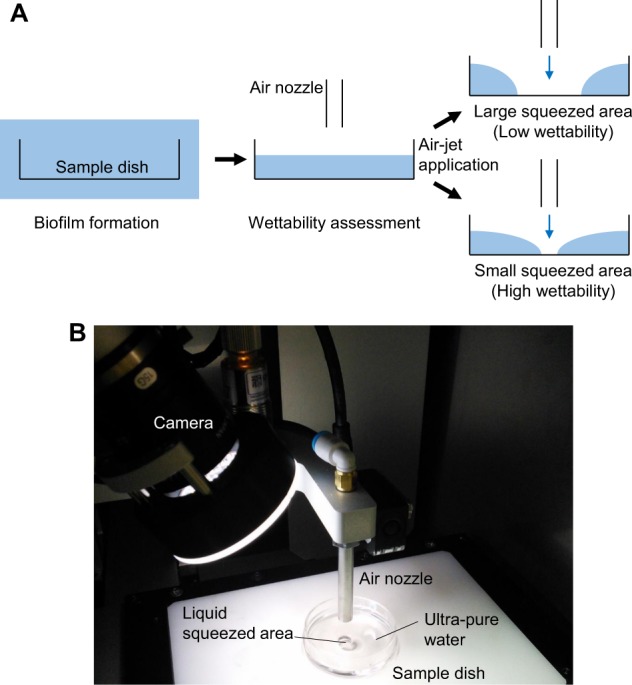
Wettability assessment by liquid squeezing. Illustration (**A**) shows a schematic diagram of the experiment from biofilm formation to wettability assessment. After the biofilm formation, the liquid covering the sample dish was squeezed by air-jet application from an air nozzle. Then, the surface wettability was assessed using the size of the liquid squeezed area. Photograph (**B**) is an overview of the experimental setup during liquid squeezing by air-jet application.

**Figure 5:**
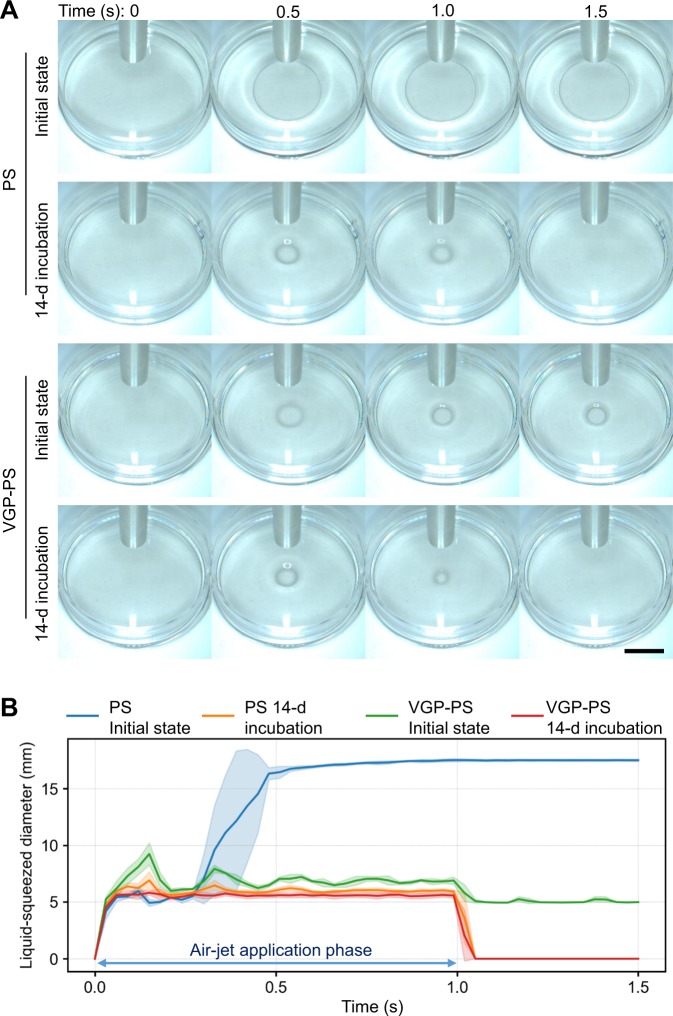
Liquid squeezing by air-jet application for differently conditioned sample dishes. Photographs (**A**) show the time-courses of liquid-squeezing behaviour by air-jet application. The row and column of these photographs indicate the time-course (0, 0.5, 1.0, and 1.5 s) and the different sample conditions, respectively. The black bar indicates 1 cm. Movies are available in Supplementary Information 1–4. Graph (**B**) shows the time-series of the liquid-squeezed diameter determined by image processing from the time-courses of photographs as shown in (**A**). Lines and lightly shaded corresponding-colour areas represent means and standard deviations, respectively (N = 3, PS initial state; N = 9, PS 14-d incubation; N = 3, VGP-PS initial state; N = 6, VGP-PS 14-d incubation). PS, polystyrene; VGP, vacuum gas plasma-treated. All original data are available in Supplementary Information 6.

The wettability on the bottom surface of the sample dish was assessed and relatively quantified by comparison of the liquid squeezed area (Fig. 4A)^20,21^. The constant volume of ultra-pure water in the dish was squeezed out around the area under the air-nozzle by air-jet application (Fig. 4B). The behaviour of liquid in the dish was monitored from camera images (Fig. 5A and Supplementary Information 1–4) and the liquid-squeezed diameter was determined in each experiment (Fig. 5B and Supplementary Information 6). A 5 kPa-compressed air-jet was applied for 1 s, and the liquid in the dishes for all conditions was squeezed during the air-jet application of t = 0 to 1 s. On the other hand, the liquid behaviours after ceasing air-jet application at t = 1 s were discriminated into two categories: (1) the remaining squeezed area was the same as in the initial state; and (2) there was complete recovery on the bottom surface by the liquid for the 14-d incubations. From the time-series data of the liquid-squeezed diameter (Fig. 5B), two-step liquid squeezing, where the liquid was squeezed to around 5 mm in diameter at 0.2 s and then further spread over 15 mm, was observed in the PS initial state condition. The liquid-squeezing diameter for the VGP-PS initial state condition oscillated with a frequency of around 6 Hz at an average diameter around 6 mm during the air-jet application. In both PS and VGP-PS 14-d incubations, the liquid-squeezed diameter simply reached a constant value and recovered to zero within 0.1 s after the beginning and ceasing of air-jet application, respectively.

### Surface wettability

**Figure 6:**
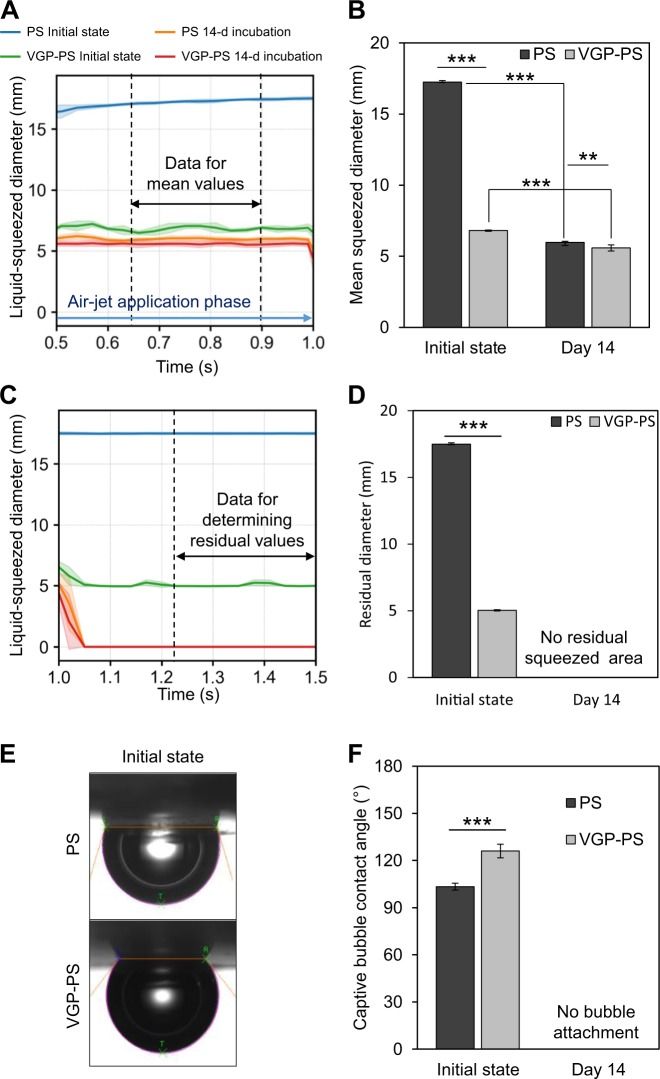
Wettability analysis for different sample dishes. Graph (**A**) shows the time-courses of liquid-squeezed diameters trimmed out at 0.5 to 1.0 s of Fig. 5B to indicate the data for mean values. Graph (**B**) compares the mean squeezed diameters. Graph (**C**) shows the time-course of liquid-squeezed diameters trimmed out at 1.0 to 1.5 s to indicate the data for determining the residual liquid-squeezed diameters. Graph (**D**) compares the residual liquid-squeezed diameters. Photographs (**E**) show a captive bubble attaching on a sample surface in water. Graph (**F**) shows the contact angles of captive bubbles. PS, polystyrene; VGP, vacuum gas plasma-treated. Lines and lightly shaded corresponding-colour areas in (**A**,**C**) or bars and error bars in (**B**,**D**,**F**) represent means and standard deviations, respectively (N = 3, PS initial state; N = 9, PS 14-d incubation; N = 3, VGP-PS initial state; N = 6, VGP-PS 14-d incubation for liquid-squeezed diameter. N = 6 for contact angle). Symbols: *p < 0.05; **p < 0.01; and ***p < 0.001 for each indicated combination. All original data are available in Supplementary Information 6.

Two indexes of the liquid-squeezed diameter were used for the quantification of wettability. The first index was the mean value of the liquid-squeezed diameter during air-jet application and it was determined as the arithmetic mean of the diameter from 0.63 to 0.9 s. That range was the later phase of liquid squeezing (Fig. 6A). For the initial dishes, the mean squeezed diameter on the PS dishes reached around 17 mm, which was 2.5 times larger than that on VGP-PS dishes which had a mean of 6.8 mm (Fig. 6B). This relationship indicated that the surface of the PS dishes was more hydrophobic than the surface of the VGP-PS dishes at the initial state. On the other hand, the mean squeezed diameters on PS and VGP-PS dishes after 14-d incubations for biofilm formation were 6.0 and 5.6 mm, respectively. The mean squeezed diameters on PS and VGP-PS dishes after the 14-d incubations were reduced to 39% and 93% of the original mean square diameters at the initial state, respectively. This result indicated that both surfaces at 14 d were becoming significantly hydrophilic in comparison with those at the initial state. The difference of mean squeezed diameters between PS and VGP-PS dishes was reduced within 8% from the initial difference of 254%.

Next, to analyse the liquid recovery behaviour, the second index of the residual diameter of liquid-squeezed area after ceasing the air-jet application from 1.23 to 1.5 s was determined (Fig. 6C). The residual diameters of PS and VGP-PS initially were 17 and 5.0 mm, respectively, and the liquid was slightly recovered for the VGP-PS dishes after ceasing the air-jet application, though the liquid never recovered for the PS dishes. In the 14-d incubations, the liquid was fully recovered on the bottom surfaces of both PS and VGP-PS dishes. Therefore, the residual diameter was determined as 0 for those dishes after the 14-d incubation.

The contact angle of a captive bubble in water attached on the bottom surface of a sample dish was also measured as a standard index of surface wettability (Fig. 6E). However, because the dishes at the 14-d incubation strongly repelled the air bubble, the contact angle could not be measured for them. For the initial state, the captive bubble contact angles on PS and VGP-PS dishes were 103 deg and 126 deg, respectively. This result indicated that the PS surface was relatively hydrophobic compared with the VGP-PS surface.

## Discussion

Because of the increasing stain densities on the dishes after the 7-d incubation as compared with those at the initial state, the biofilm was speculated to be well formed on both PS and VGP-PS dishes. Since small objects having rod- or sphere-like shapes and other larger structures were observed by confocal scanning laser microscopy for the 14-d incubated dishes, the steps in the biofilm formation in this bioreactor were inferred to include not only physical adsorption of substances, resulting in the formation of a conditioning film, but also, the growth of microorganisms and the accumulation of EPSs. However, the dispersion stage was unable to be determined in this experiment, although the biofilm detachment occurred on the PS dish at 28 d. In previous studies^22,23^, incubation with the bioreactor successfully yielded well-formed biofilms on various types of material surfaces including glass, metals, and plastics other than PS. Therefore, this incubation system was judged as applicable to the biofilm formation on PS and VGP-PS surfaces. Various types of surface modifications affecting hydrophobic and hydrophilic properties have been investigated for reduction of biofilm formation^24–28^, and in the present study both hydrophilic VGP-PS and hydrophobic bare PS surfaces had no remarkable effect for preventing biofilm formation. The cause of biofilm formation on both PS and VGP-PS dishes was speculated to be the enhancement of physical absorption such as by a hydrophobic effect in the PS case and hydrogen bond formation to a hydroxy group in the VGP-PS case^29^. Hydrophobic PS surfaces generally cause the hydrophobic effect which enhances the physical absorption of hydrophobic sites of molecules in water onto hydrophobic surfaces by repelling the surrounding water^30^. On the other hand, because the VGP process introduces hydroxy groups onto a surface, a hydrogen bond occurs between the hydroxy groups on the VGP surface and some types or parts of molecules in solution. Therefore, the hydrogen bond on the VGP-PS surface was speculated as assisting the physical absorption of molecules onto that surface^31^.

The 14-d incubations in both PS and VGP-PS cases produced the biofilm that was the same as the biofilm formed following the 21-d incubation and relatively stabler in comparison with the early stage biofilm for the 7-d incubation or the biofilm for the longer 28-d incubation. Those PS and VGP-PS surfaces at 14 d were more hydrophilic than the original surfaces, even in the case of the originally hydrophilic VGP-PS, the surface with the biofilm was more hydrophilic than the original one. Because the contact angle of the captive bubble on the PS dish surface was smaller than that on the VGP-PS one, the PS and VGP-PS dishes at the initial state had relatively hydrophobic and hydrophilic properties, respectively. This result agreed with the general understanding of the VGP process, which can change a surface into a more hydrophilic by the addition of the hydroxy group onto the surface. In a previous study regarding liquid-squeezing-based wettability assessment^21^, a clear relationship between the liquid-squeezed diameter and contact angle of the water droplet onto bare and atmospheric pressure nitrogen gas plasma-treated polystyrene surfaces was confirmed. The larger liquid-squeezed diameter during air-jet application and the residual squeezed area on the PS dish indicated this dish had a better hydrophobic property compared with the VGP-PS dish, and the results agreed with the results from the captive bubble method.

Although the contact angle of the captive bubble could not be measured due to no attachment of the air bubble for biofilm-formed dishes, the liquid-squeezing approach worked on these dishes. Both dishes after the biofilm formation had smaller mean values of liquid-squeezed diameter during air-jet application and the liquids were fully recovered over the dishes, resulting in no residual squeezed area. Therefore, liquid-squeezing-based wettability assessment was an appropriate method for detecting biofilm formation via its wettability change. *In situ* Raman spectroscopy^32^ and FTIR spectroscopy^33^ have revealed the existence of lipids, which are fundamental hydrophobic substances, in biofilms. However, the lipids were speculated to be embedded in a micelle structure in the EPSs or microorganisms because of interaction by surrounding water, therefore, the wettability of surfaces on both PS and VGP-PS dishes expressed hydrophilicity. Especially, because of the non-zero values in the case of biofilm-formed dishes, instead of zero in the residual squeezed diameter, the mean value of liquid-squeezed diameter during air-jet application was a more useful index for quantitative evaluation.

To date, various types of *in-situ* approaches for biofilm characterization have been proposed and practically used (Table 1). The present wettability approach has the two important features: (1) the capability for *in-situ* assessment of an intact hydrous biofilm and (2) the capability for macroscopic representation of physicochemical surface information via the wettability index (liquid-squeezed diameter). Unfortunately, though local detailed information such as a microscopic surface profile cannot be obtained directly, the wettability approach is useful for the simple assessment of surface change in the incubation of anti-biofilm material development with complementary use of a microscopic approach.

**Table 1:** *In-situ* approaches^11^ for biofilm characterization with additional references.

Approach	Features	Reference
Optical microscopy	Local observation of planar microstructure in an intact biofilm in case of no staining.	^38, 39^
Confocal laser scanning microscopy (CLSM)	Volumeric microstructure of biofilm can be observed *in situ* from laser intensity without staining.	^40– 44^
Environmental scanning electron microscopy	Observation of local microstructure in near-atmospherical vacuum environment with higher resolution than optical diffraction limit.	^45, 46^
Raman spectroscopy	Chemical structures in biofilm can be assessed *in situ*.	^22, 39, 47^
Fourier transform infraredspectroscopy (FTIR)	The chemical functional groups in biofilm can be analysed macroscopically *in situ* in case of attenued total reflection technique.	^33, 34, 47^
Quartz crystal microbalance (QCM)	Time-series biofilm formation can be indirectly detected *in situ* via frequency changes.	^48^
Wettability	*In-situ* macroscopic representation of physicochemical information of intact hydrous biofilm.	This study

## Methods

### Biofilm formation

Biofilm formation with a bioreactor (Fig. 1B) was performed as previously reported^22,23,34^. Two types of substrates were used for sampling biofilms: (1) a polystyrene petri dish (PS) (Falcon 351008) (Corning, Corning, NY); and (2) a vacuum-gas-plasma-treated polystyrene dish (VGP-PS) (Falcon 353001) (Corning). In advance of biofilm formation, a plastic mesh basket was positioned obliquely above an open-top water tank (water volume of 12 L) and ambient air was collected by an electric fan (MU1025S-11N) (Oriental Motor, Tokyo, Japan) and introduced onto the water flow passing over the mesh basket for over a month. The water flow that circulates in the bioreactor was generated by a water pump (PMD-221B2M) (Sanso Electric, Hyogo, Japan). The substrate was immersed into the water tank and placed onto the tank bottom in the bioreactor, and then biofilm formation was carried out with water circulation without air flow by the fan for 28 d at 27 ± 1 °C. The flow rates of air blown by the electric fan and circulating water were set at 1.4 m^3^/min and 5 L/min, respectively.

### Staining and quantification

To confirm the biofilm formation, crystal violet staining was performed^35,36^. In brief, the water-immersed dishes were removed from the water tank at incubations of 7, 14, 21, and 28 d. A bare dish was used as the initial state condition and was referred to as the 0-d incubation. After water that remained inside the selected dish was removed, 0.1 w/v% crystal violet aqueous solution was added into the dish by pouring it gently down the dish wall. After the incubation at room temperature for 30 min, the added crystal violet aqueous solution was removed, and the dish was immersed into tap water. After being air-dried, the stained dish was photographed with an image scanner (MG6930) (Canon, Tokyo, Japan).

**Figure 7:**
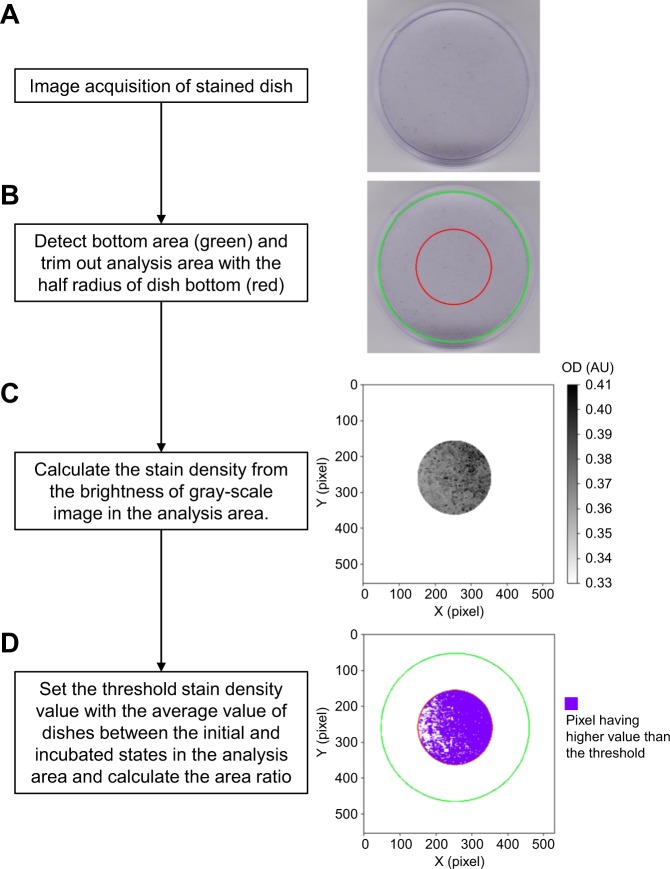
Flow chart of quantification for staining. The homemade software had a four-step process: (**A**) acquiring an image; (**B**) setting the analysis area; (**C**) calculating the stain density; and (**D**) calculating the area ratio. Images on the right side of the flow chart (**A**–**D**) correspond to the process results. The software code is available in Supplementary Information 5.

From the image of the stained dish (Fig. 7A), the density of the stain was quantified by assuming it was represented by optical density (OD)^37^. Briefly, the centre part of the dish bottom with half the radius of the dish bottom was trimmed out as an analysis area (Fig. 7B). The brightness of each pixel in the gray scale image *I* was converted into the brightness without compensation for visual display *I*′ by Eq. (1) (Fig. 7C):

1 $$I^{\prime} =-\frac{\gamma }{{\rm{I}}{\rm{n}}\,10}\text{In}(\frac{I}{{2}^{b}-1})$$

where *γ* and *b* are the gamma value of the scanner (=1.8 in this study) and the bit depth of the image (=8 bits in this study), respectively. The stain density *d* was calculated by Eq. (2) with two coefficients c_0_ and c_1_ determined from the linear relationship between OD standards (visible light ND filters No. 4; OD = 0.6, 8; OD = 0.9 and 16; OD = 1.2) (Nikon, Tokyo, Japan) and the corresponding values of *I*′ in advance (Supplementary Information 5):

2 $$d={c}_{1}I^{\prime} +{c}_{0}$$

After setting the threshold d value of each pixel with the average value of dishes between the initial and incubated states, the ratio of stained area per unit area was calculated (Fig. 7D).

### Confocal scanning laser microscopy

After being air-dried, the 14-d incubated surfaces on PS and VGP-PS dishes were observed with a confocal scanning laser microscope (VK-8710) (Keyence, Osaka, Japan). The observed data were three-dimensionally reconstructed using the accessory software of the microscope (VK Analyzer) (Keyence).

### Wettability assessment

The surface wettability of sample dishes was assessed by the previously proposed liquid-squeezing method^20,21^. In brief, the sample dish was rinsed with fresh ultra-pure water, the rinsing water was carefully removed from the dish, and then 1.5 mL of ultra-pure water was poured into the dish for the assessment. After the sample dish with the constant volume of water was set on the flat stage of a wettability assessment system (prototype) (Kitagawa Corporation, Hiroshima, Japan) (Fig. 4B), air compressed at 5 kPa was released from an air nozzle (i.d., 0.5 mm) positioned at a height of 15 mm from the water surface in the dish. The behaviour of liquid during air-jet application was monitored by the accessory digital camera of the system. The diameter of the liquid-squeezed area was automatically determined from the monitored images with the accessory image processing software of the system.

As a conventional method, the captive bubble contact angle measurement was employed for assessing the wettability of sample surfaces in liquid. Before the contact angle measurement, the bottom surface of the sample dish was trimmed out with an ultrasonic cutter (USW-334) (Honda Electronics, Aichi, Japan) to get a 1-cm width specimen, and then, the specimen was placed with its bottom surface up and immersed into the ultra-pure water that filled the water chamber of a contact angle meter (DMs-401) (Kyowa Interface Science, Saitama, Japan). A single bubble with a volume of 2 to 5 μL was formed onto the specimen surface, and then, the angle of the attached bubble was automatically measured.

### Data analysis

For the liquid-squeezed diameter, two different indexes were defined: (1) the mean value during the air-jet application phase (t = 0.63 to 0.9 s); and (2) the residual diameter as a mean value after ceasing the air-jet application (t = 1.23 to 1.5 s). Those two indexes were determined for each liquid-squeezing experiment.

Statistical significance between two data sets was confirmed with Student’s t-test for equal variances or Welch’s t-test for unequal ones after homoscedasticity confirmation with an F-test.

## Supplementary information

Supplementary Information 1 Liquid-squeezing behaviour by air-jet application on a bare polystyrene dish at the initial state.

Supplementary Information 2 Liquid-squeezing behaviour by air-jet application on a polystyrene dish after the 14-d incubation.

Supplementary Information 3 Liquid-squeezing behaviour by air-jet application on a vacuum-gas-plasma treated polystyrene dish at the initial state.

Supplementary Information 4 Liquid-squeezing behaviour by air-jet application on a vacuum-gas-plasma treated polystyrene dish after the 14-d incubation.

Supplementary Information 5 A dataset of crystal violet staining experiment including raw images, software code and analysis results.

Supplementary Information 6 A Microsoft Excel spreadsheet including all data in the wettability assessment and data analysis.
