# Supplementary figures and images for: In-situ detection based on the biofilm hydrophilicity for environmental biofilm formation (part 2 of 2)
Source: Sci Rep. 2019 May 30;9:8070. doi: 10.1038/s41598-019-44167-6 (PMC6542837; doi:10.1038/s41598-019-44167-6)

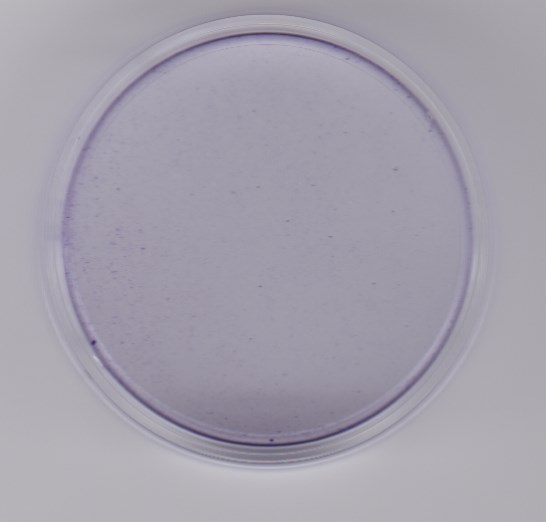

Supplement: Supplementary file 5 — Supplementary Information 5 A dataset of crystal violet staining experiment including raw images, software code and analysis results. [file 41598_2019_44167_MOESM5_ESM.zip › images/d07_v_2.jpg]

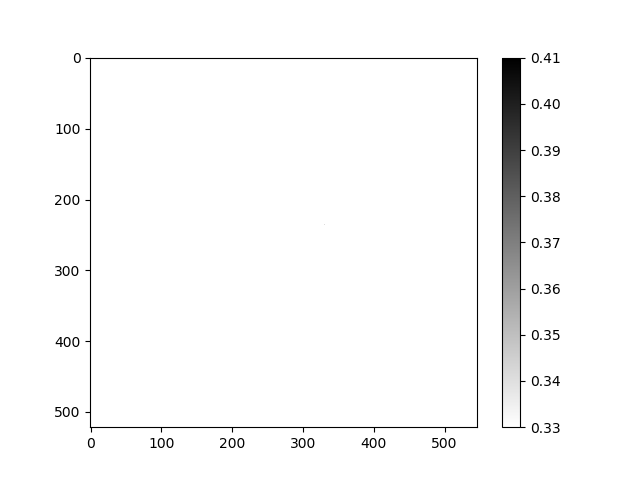

Supplement: Supplementary file 5 — Supplementary Information 5 A dataset of crystal violet staining experiment including raw images, software code and analysis results. [file 41598_2019_44167_MOESM5_ESM.zip › images/d07_v_2.jpg.1.png]

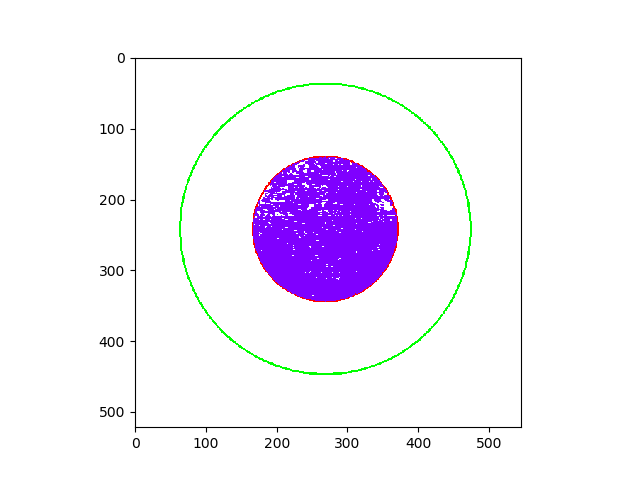

Supplement: Supplementary file 5 — Supplementary Information 5 A dataset of crystal violet staining experiment including raw images, software code and analysis results. [file 41598_2019_44167_MOESM5_ESM.zip › images/d07_v_2.jpg.2.png]

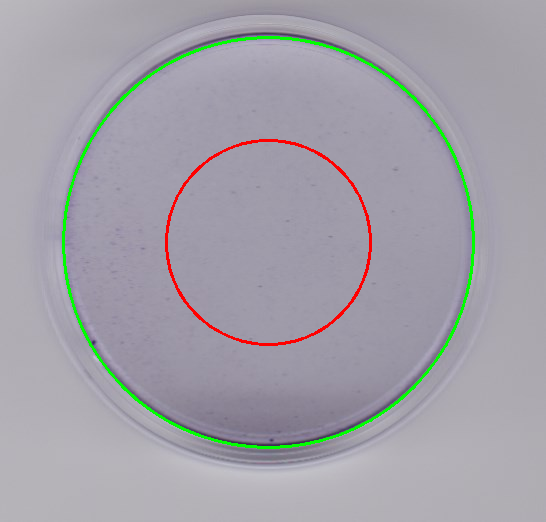

Supplement: Supplementary file 5 — Supplementary Information 5 A dataset of crystal violet staining experiment including raw images, software code and analysis results. [file 41598_2019_44167_MOESM5_ESM.zip › images/d07_v_2.jpg.tiff]

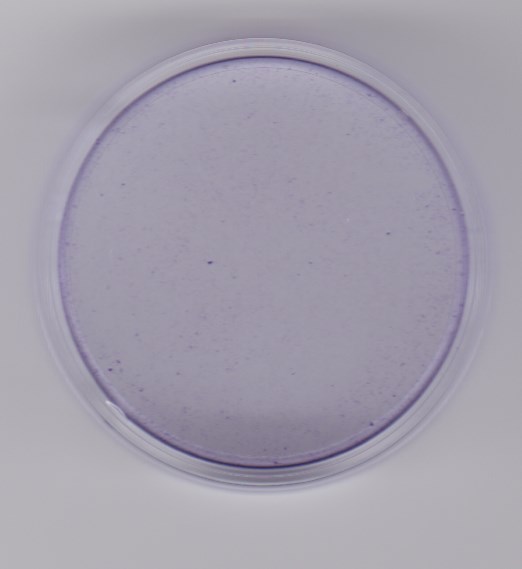

Supplement: Supplementary file 5 — Supplementary Information 5 A dataset of crystal violet staining experiment including raw images, software code and analysis results. [file 41598_2019_44167_MOESM5_ESM.zip › images/d07_v_3.jpg]

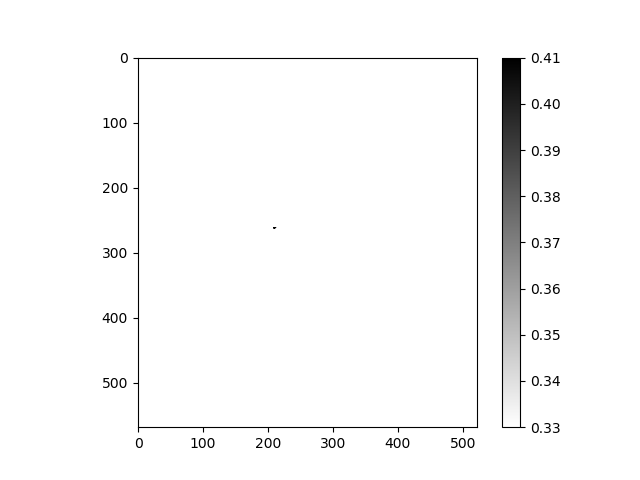

Supplement: Supplementary file 5 — Supplementary Information 5 A dataset of crystal violet staining experiment including raw images, software code and analysis results. [file 41598_2019_44167_MOESM5_ESM.zip › images/d07_v_3.jpg.1.png]

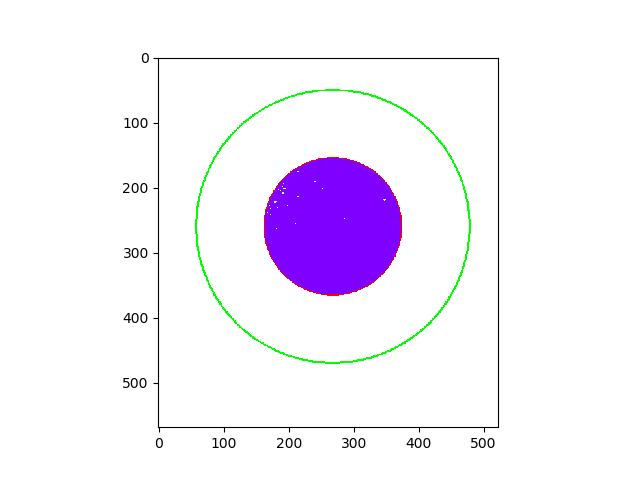

Supplement: Supplementary file 5 — Supplementary Information 5 A dataset of crystal violet staining experiment including raw images, software code and analysis results. [file 41598_2019_44167_MOESM5_ESM.zip › images/d07_v_3.jpg.2.png]

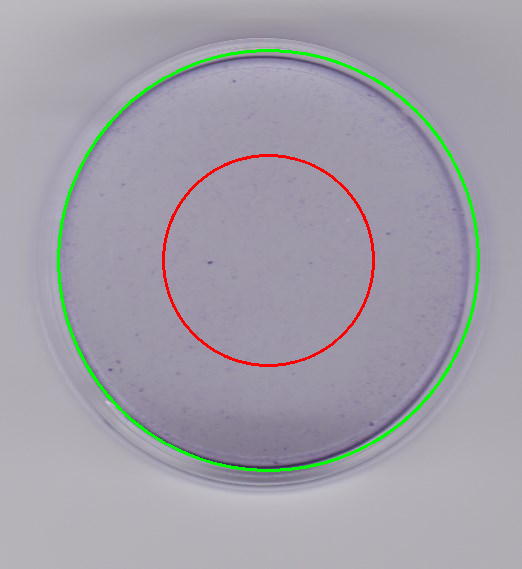

Supplement: Supplementary file 5 — Supplementary Information 5 A dataset of crystal violet staining experiment including raw images, software code and analysis results. [file 41598_2019_44167_MOESM5_ESM.zip › images/d07_v_3.jpg.tiff]

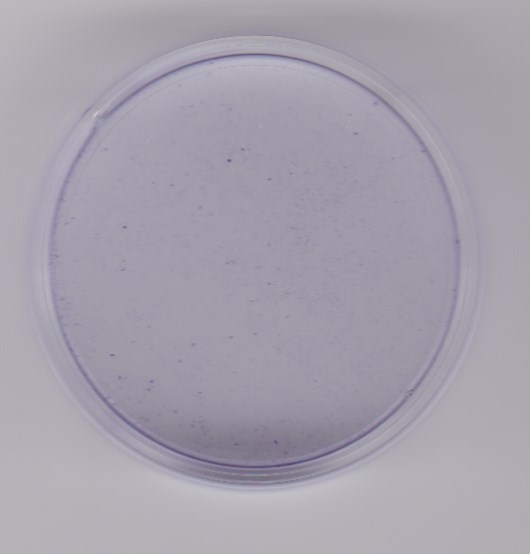

Supplement: Supplementary file 5 — Supplementary Information 5 A dataset of crystal violet staining experiment including raw images, software code and analysis results. [file 41598_2019_44167_MOESM5_ESM.zip › images/d07_v_4.jpg]

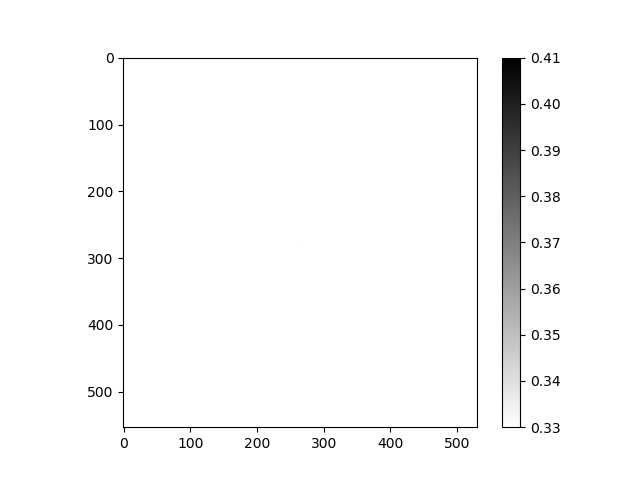

Supplement: Supplementary file 5 — Supplementary Information 5 A dataset of crystal violet staining experiment including raw images, software code and analysis results. [file 41598_2019_44167_MOESM5_ESM.zip › images/d07_v_4.jpg.1.png]

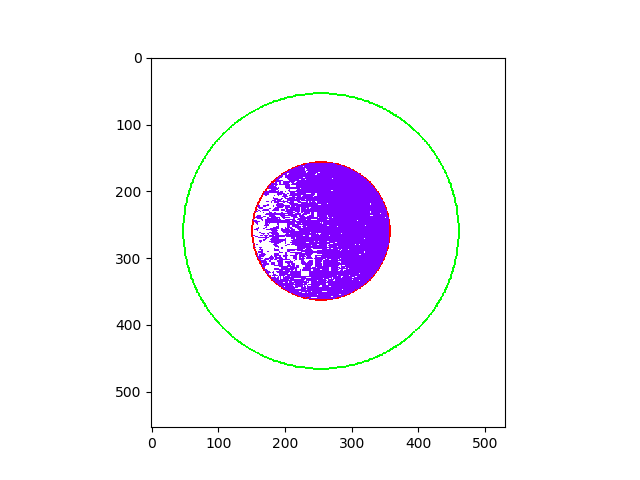

Supplement: Supplementary file 5 — Supplementary Information 5 A dataset of crystal violet staining experiment including raw images, software code and analysis results. [file 41598_2019_44167_MOESM5_ESM.zip › images/d07_v_4.jpg.2.png]

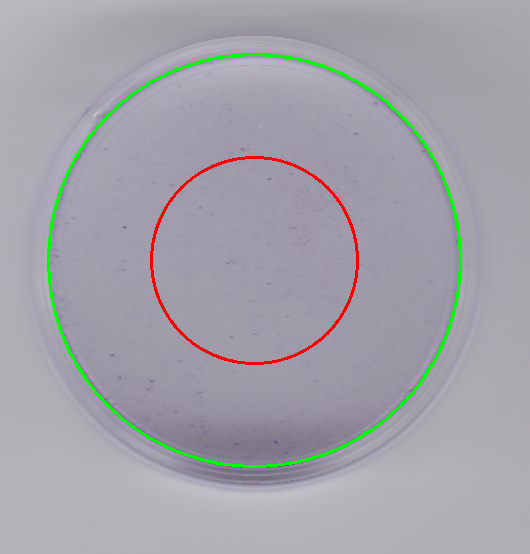

Supplement: Supplementary file 5 — Supplementary Information 5 A dataset of crystal violet staining experiment including raw images, software code and analysis results. [file 41598_2019_44167_MOESM5_ESM.zip › images/d07_v_4.jpg.tiff]

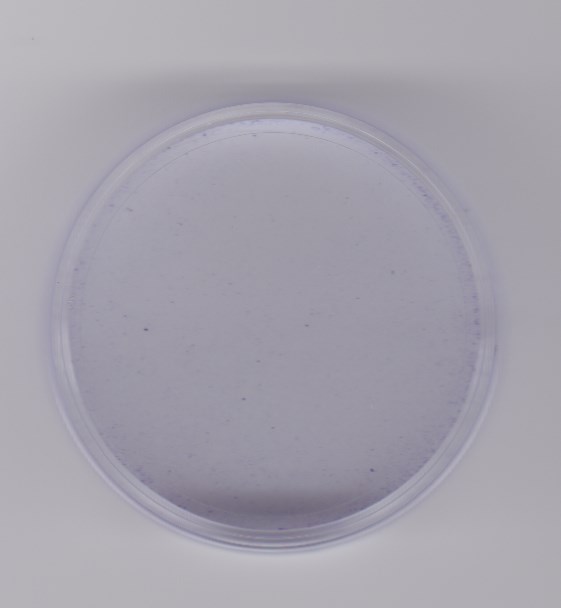

Supplement: Supplementary file 5 — Supplementary Information 5 A dataset of crystal violet staining experiment including raw images, software code and analysis results. [file 41598_2019_44167_MOESM5_ESM.zip › images/d07_v_5.jpg]

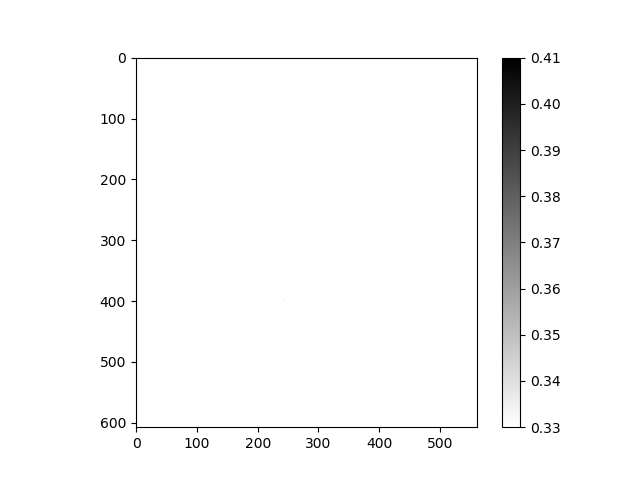

Supplement: Supplementary file 5 — Supplementary Information 5 A dataset of crystal violet staining experiment including raw images, software code and analysis results. [file 41598_2019_44167_MOESM5_ESM.zip › images/d07_v_5.jpg.1.png]

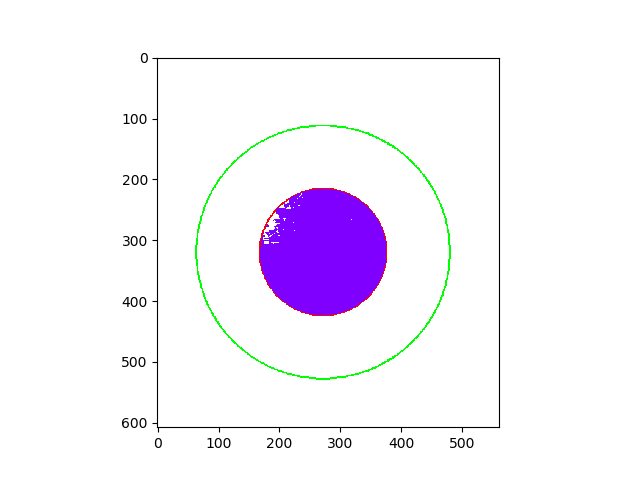

Supplement: Supplementary file 5 — Supplementary Information 5 A dataset of crystal violet staining experiment including raw images, software code and analysis results. [file 41598_2019_44167_MOESM5_ESM.zip › images/d07_v_5.jpg.2.png]

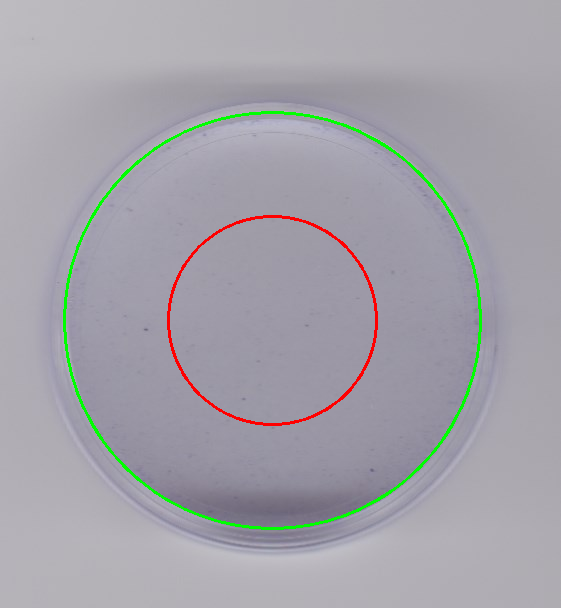

Supplement: Supplementary file 5 — Supplementary Information 5 A dataset of crystal violet staining experiment including raw images, software code and analysis results. [file 41598_2019_44167_MOESM5_ESM.zip › images/d07_v_5.jpg.tiff]

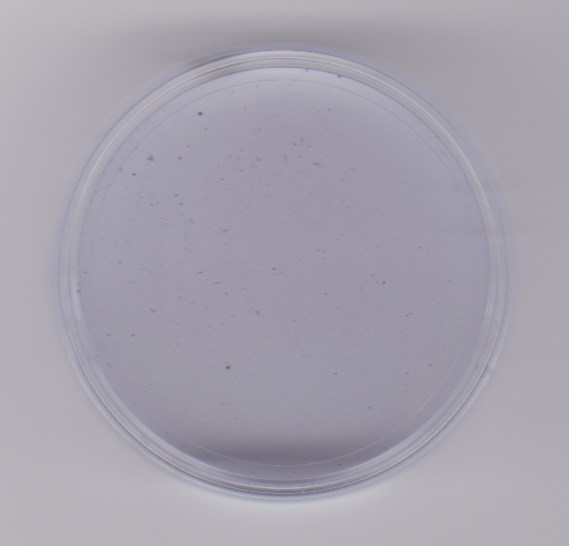

Supplement: Supplementary file 5 — Supplementary Information 5 A dataset of crystal violet staining experiment including raw images, software code and analysis results. [file 41598_2019_44167_MOESM5_ESM.zip › images/d14_p_1.jpg]

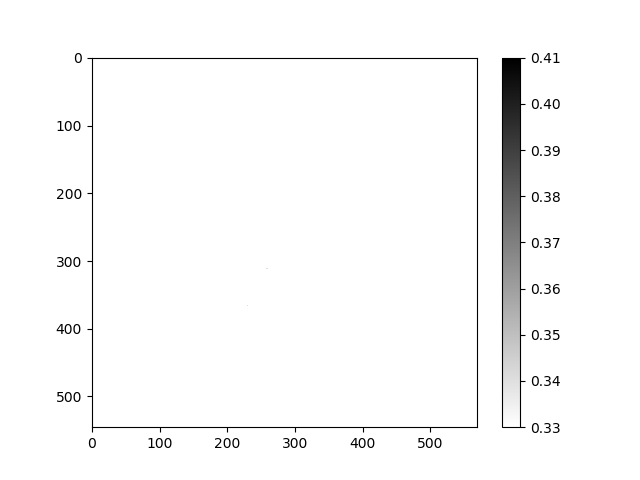

Supplement: Supplementary file 5 — Supplementary Information 5 A dataset of crystal violet staining experiment including raw images, software code and analysis results. [file 41598_2019_44167_MOESM5_ESM.zip › images/d14_p_1.jpg.1.png]

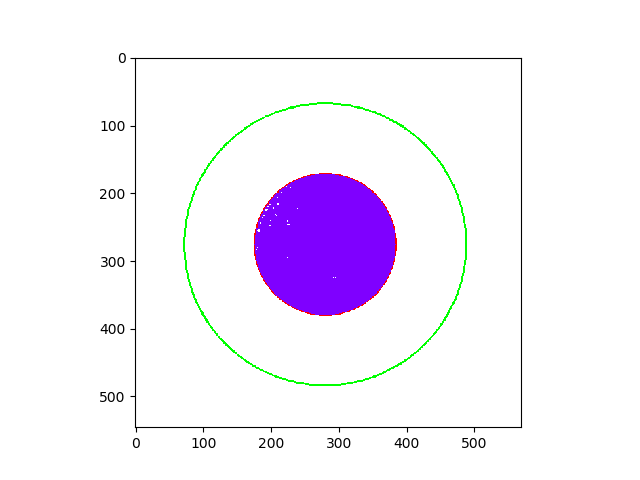

Supplement: Supplementary file 5 — Supplementary Information 5 A dataset of crystal violet staining experiment including raw images, software code and analysis results. [file 41598_2019_44167_MOESM5_ESM.zip › images/d14_p_1.jpg.2.png]

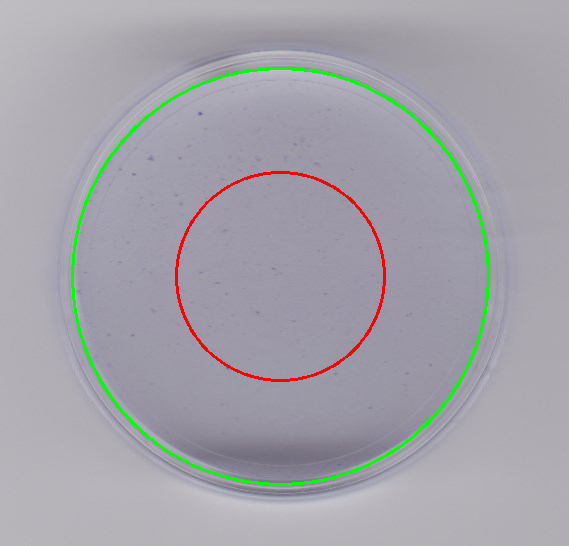

Supplement: Supplementary file 5 — Supplementary Information 5 A dataset of crystal violet staining experiment including raw images, software code and analysis results. [file 41598_2019_44167_MOESM5_ESM.zip › images/d14_p_1.jpg.tiff]

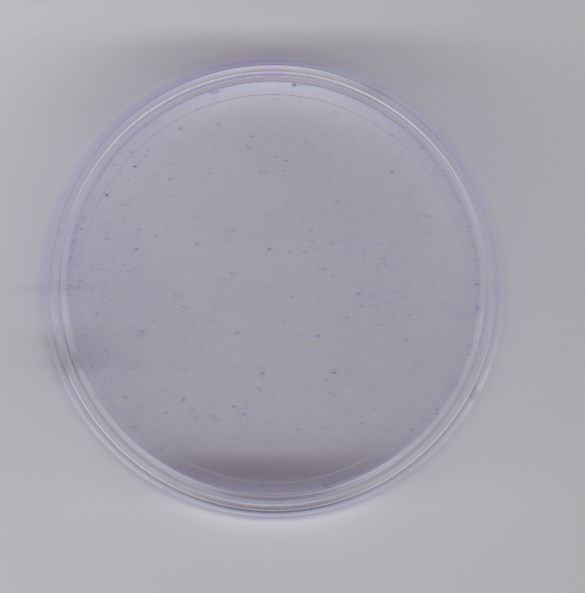

Supplement: Supplementary file 5 — Supplementary Information 5 A dataset of crystal violet staining experiment including raw images, software code and analysis results. [file 41598_2019_44167_MOESM5_ESM.zip › images/d14_p_2.jpg]

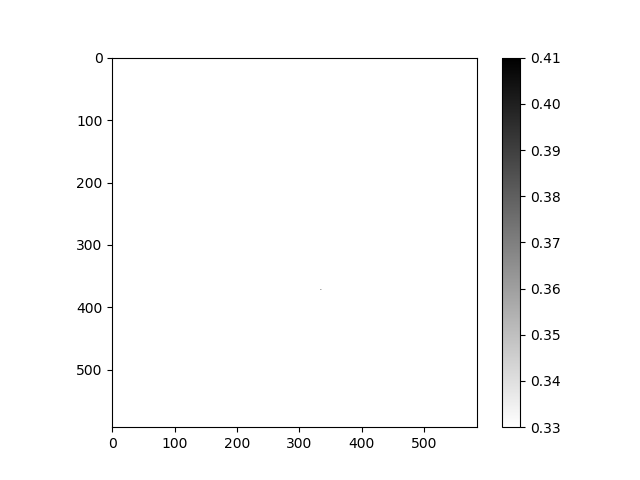

Supplement: Supplementary file 5 — Supplementary Information 5 A dataset of crystal violet staining experiment including raw images, software code and analysis results. [file 41598_2019_44167_MOESM5_ESM.zip › images/d14_p_2.jpg.1.png]

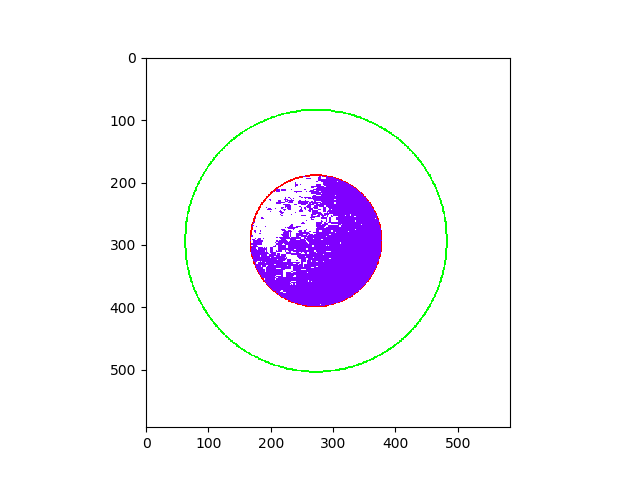

Supplement: Supplementary file 5 — Supplementary Information 5 A dataset of crystal violet staining experiment including raw images, software code and analysis results. [file 41598_2019_44167_MOESM5_ESM.zip › images/d14_p_2.jpg.2.png]

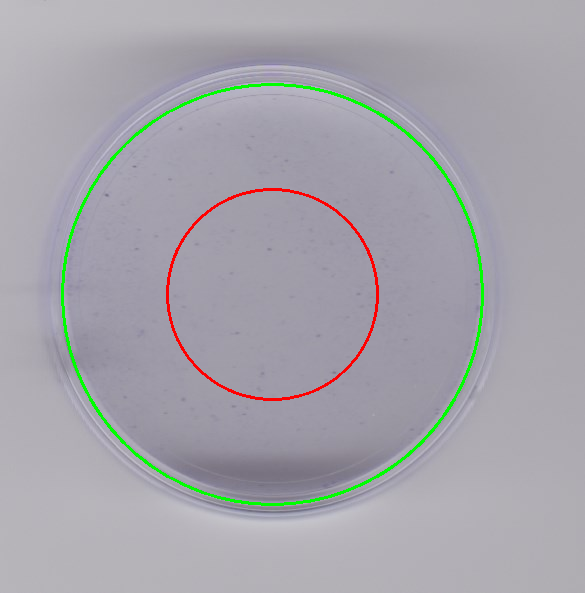

Supplement: Supplementary file 5 — Supplementary Information 5 A dataset of crystal violet staining experiment including raw images, software code and analysis results. [file 41598_2019_44167_MOESM5_ESM.zip › images/d14_p_2.jpg.tiff]

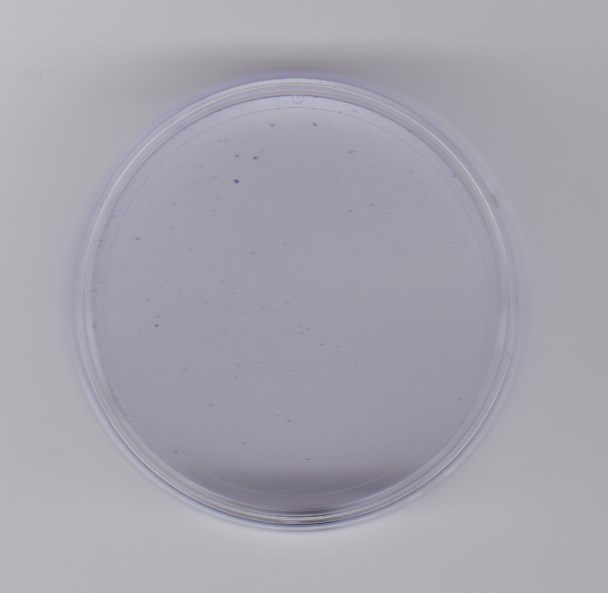

Supplement: Supplementary file 5 — Supplementary Information 5 A dataset of crystal violet staining experiment including raw images, software code and analysis results. [file 41598_2019_44167_MOESM5_ESM.zip › images/d14_p_3.jpg]

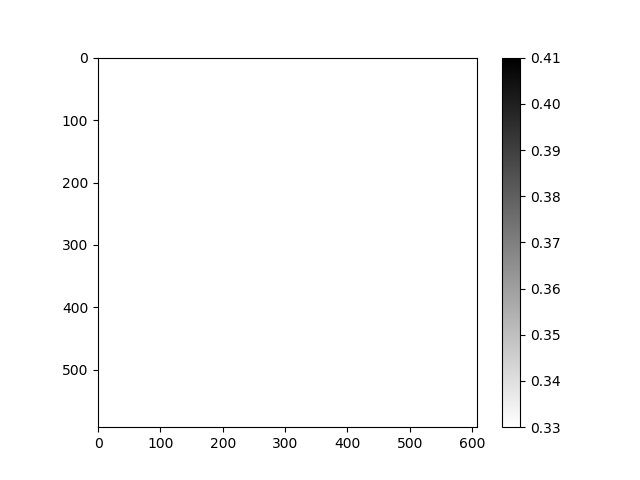

Supplement: Supplementary file 5 — Supplementary Information 5 A dataset of crystal violet staining experiment including raw images, software code and analysis results. [file 41598_2019_44167_MOESM5_ESM.zip › images/d14_p_3.jpg.1.png]

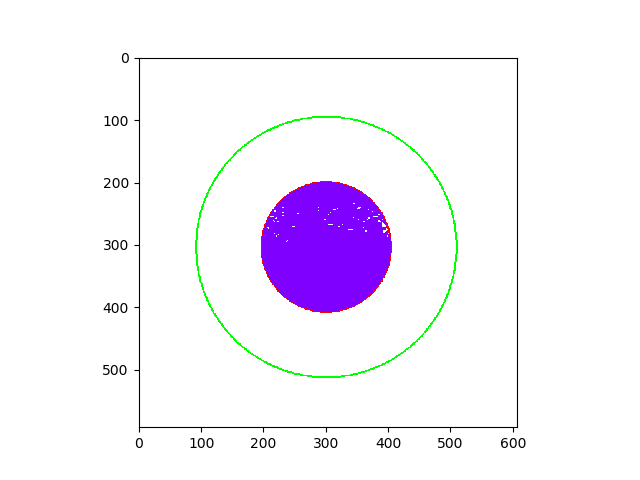

Supplement: Supplementary file 5 — Supplementary Information 5 A dataset of crystal violet staining experiment including raw images, software code and analysis results. [file 41598_2019_44167_MOESM5_ESM.zip › images/d14_p_3.jpg.2.png]

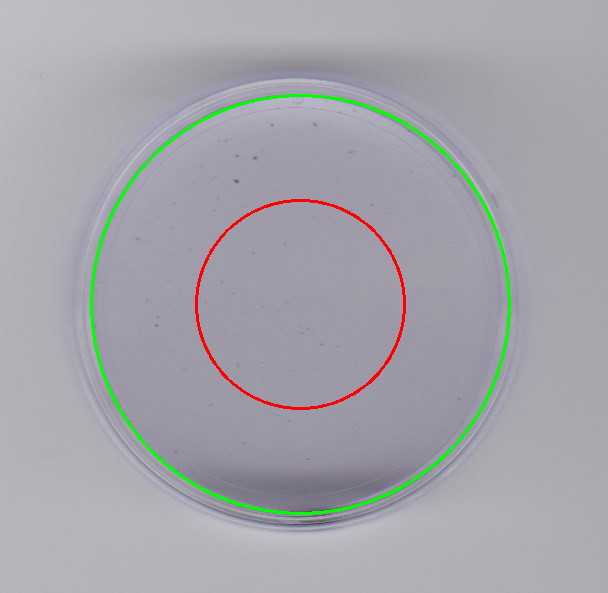

Supplement: Supplementary file 5 — Supplementary Information 5 A dataset of crystal violet staining experiment including raw images, software code and analysis results. [file 41598_2019_44167_MOESM5_ESM.zip › images/d14_p_3.jpg.tiff]

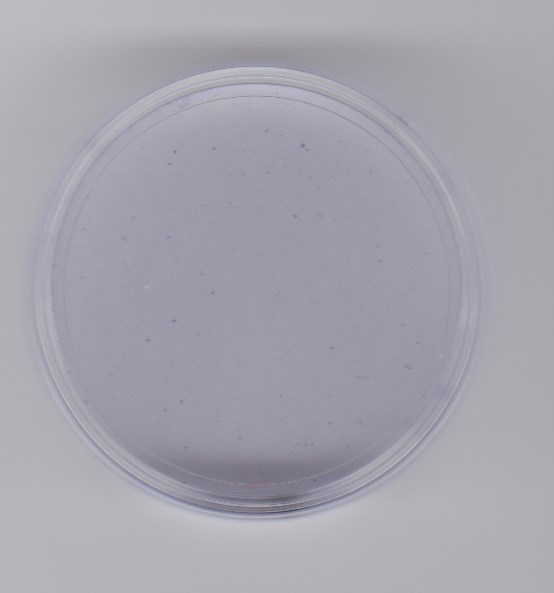

Supplement: Supplementary file 5 — Supplementary Information 5 A dataset of crystal violet staining experiment including raw images, software code and analysis results. [file 41598_2019_44167_MOESM5_ESM.zip › images/d14_p_4.jpg]

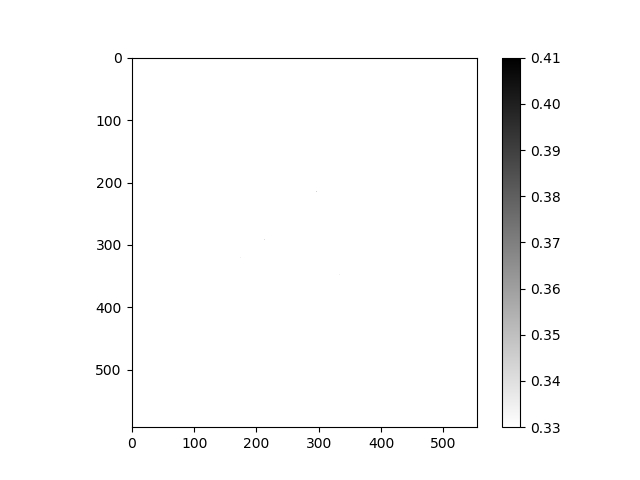

Supplement: Supplementary file 5 — Supplementary Information 5 A dataset of crystal violet staining experiment including raw images, software code and analysis results. [file 41598_2019_44167_MOESM5_ESM.zip › images/d14_p_4.jpg.1.png]

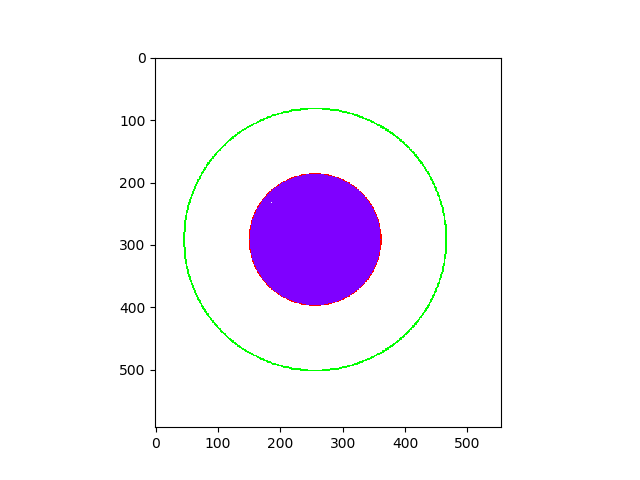

Supplement: Supplementary file 5 — Supplementary Information 5 A dataset of crystal violet staining experiment including raw images, software code and analysis results. [file 41598_2019_44167_MOESM5_ESM.zip › images/d14_p_4.jpg.2.png]

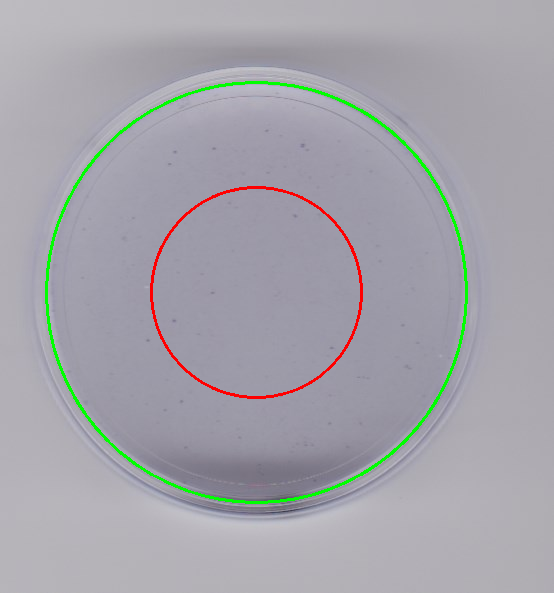

Supplement: Supplementary file 5 — Supplementary Information 5 A dataset of crystal violet staining experiment including raw images, software code and analysis results. [file 41598_2019_44167_MOESM5_ESM.zip › images/d14_p_4.jpg.tiff]

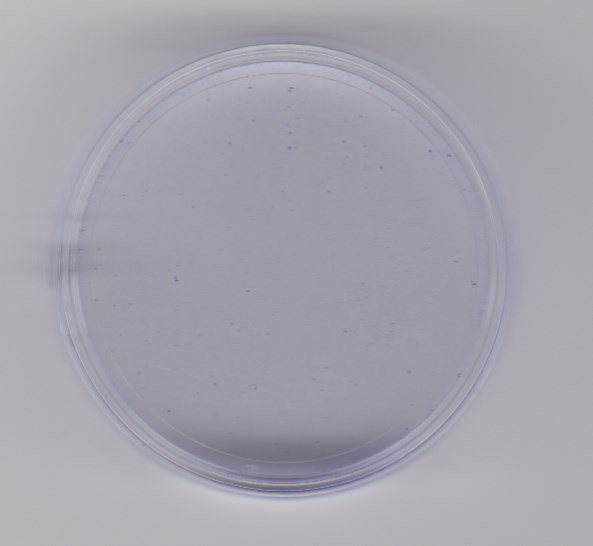

Supplement: Supplementary file 5 — Supplementary Information 5 A dataset of crystal violet staining experiment including raw images, software code and analysis results. [file 41598_2019_44167_MOESM5_ESM.zip › images/d14_p_5.jpg]

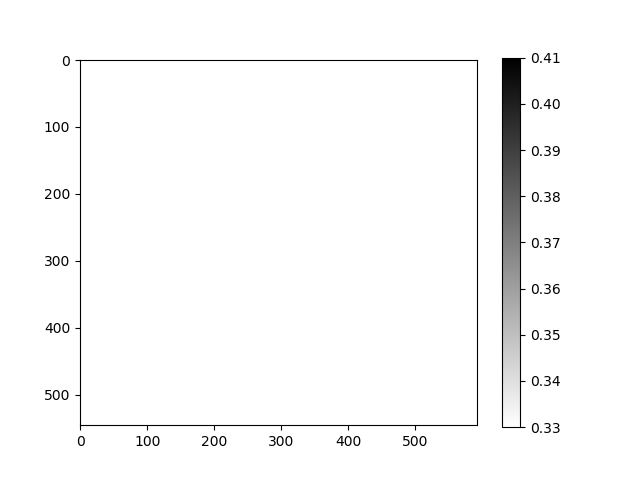

Supplement: Supplementary file 5 — Supplementary Information 5 A dataset of crystal violet staining experiment including raw images, software code and analysis results. [file 41598_2019_44167_MOESM5_ESM.zip › images/d14_p_5.jpg.1.png]

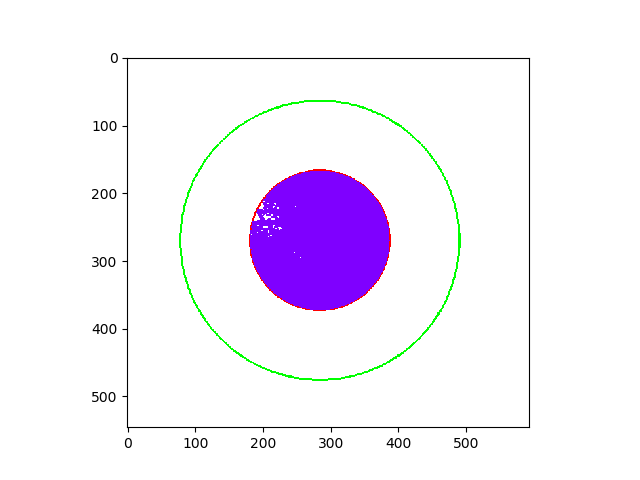

Supplement: Supplementary file 5 — Supplementary Information 5 A dataset of crystal violet staining experiment including raw images, software code and analysis results. [file 41598_2019_44167_MOESM5_ESM.zip › images/d14_p_5.jpg.2.png]

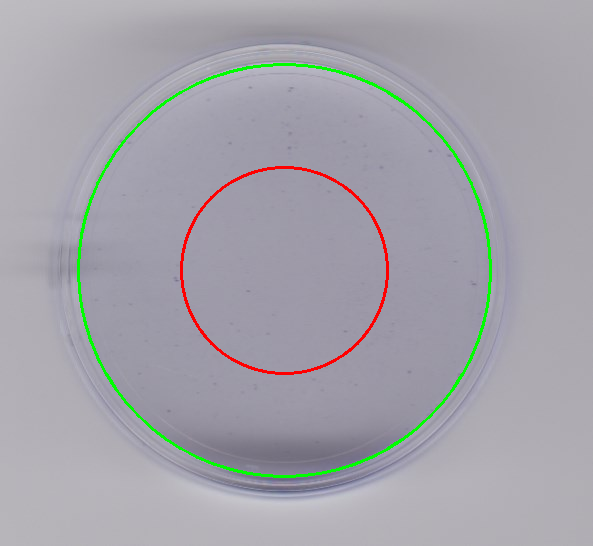

Supplement: Supplementary file 5 — Supplementary Information 5 A dataset of crystal violet staining experiment including raw images, software code and analysis results. [file 41598_2019_44167_MOESM5_ESM.zip › images/d14_p_5.jpg.tiff]

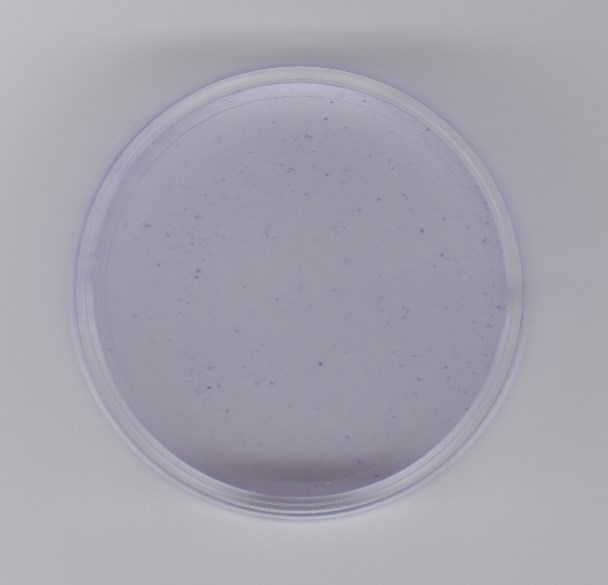

Supplement: Supplementary file 5 — Supplementary Information 5 A dataset of crystal violet staining experiment including raw images, software code and analysis results. [file 41598_2019_44167_MOESM5_ESM.zip › images/d14_v_1.jpg]

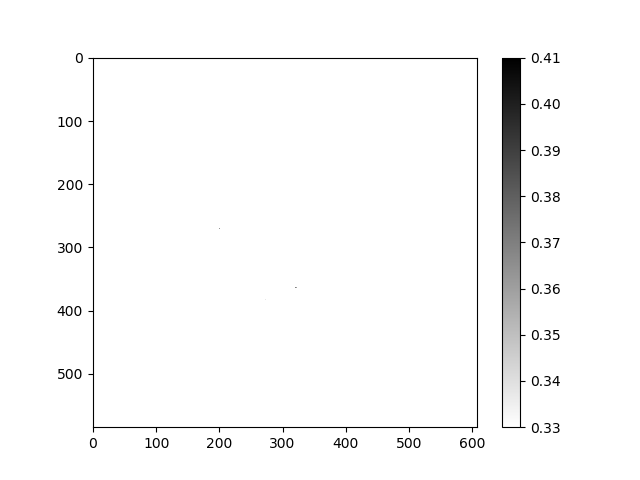

Supplement: Supplementary file 5 — Supplementary Information 5 A dataset of crystal violet staining experiment including raw images, software code and analysis results. [file 41598_2019_44167_MOESM5_ESM.zip › images/d14_v_1.jpg.1.png]

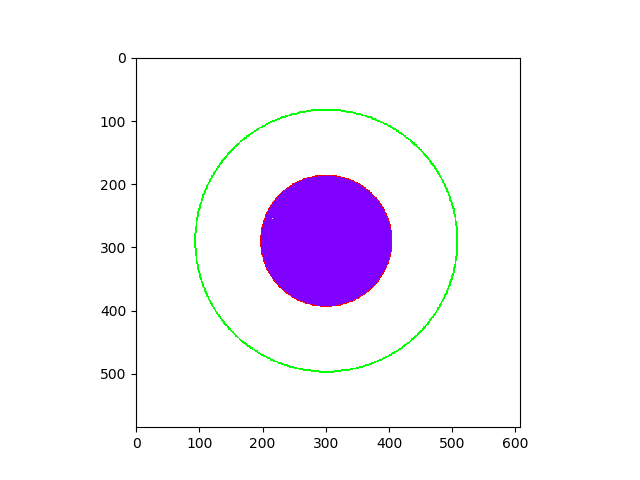

Supplement: Supplementary file 5 — Supplementary Information 5 A dataset of crystal violet staining experiment including raw images, software code and analysis results. [file 41598_2019_44167_MOESM5_ESM.zip › images/d14_v_1.jpg.2.png]

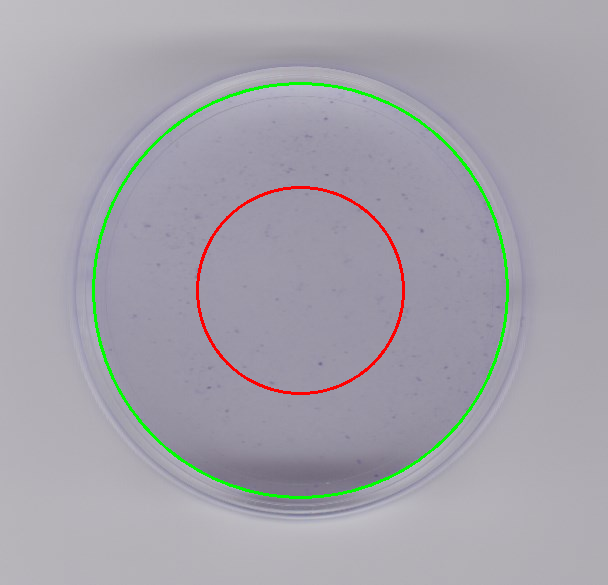

Supplement: Supplementary file 5 — Supplementary Information 5 A dataset of crystal violet staining experiment including raw images, software code and analysis results. [file 41598_2019_44167_MOESM5_ESM.zip › images/d14_v_1.jpg.tiff]

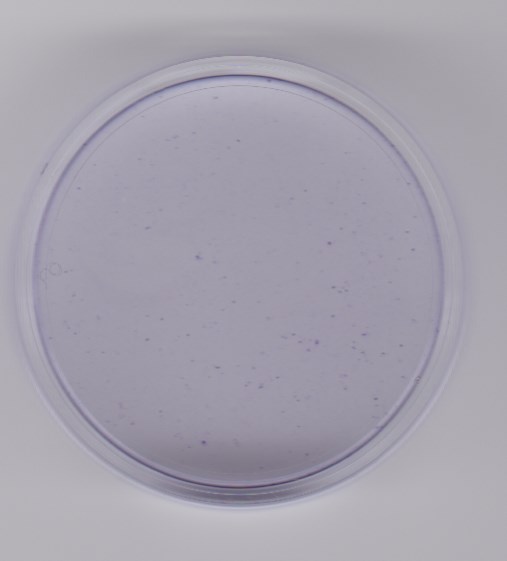

Supplement: Supplementary file 5 — Supplementary Information 5 A dataset of crystal violet staining experiment including raw images, software code and analysis results. [file 41598_2019_44167_MOESM5_ESM.zip › images/d14_v_2.jpg]

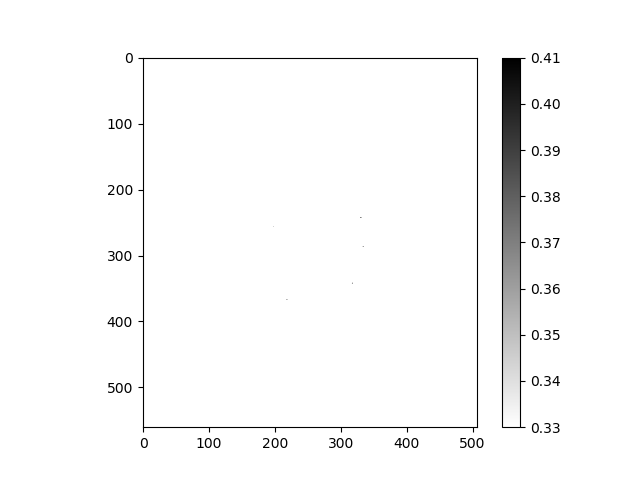

Supplement: Supplementary file 5 — Supplementary Information 5 A dataset of crystal violet staining experiment including raw images, software code and analysis results. [file 41598_2019_44167_MOESM5_ESM.zip › images/d14_v_2.jpg.1.png]

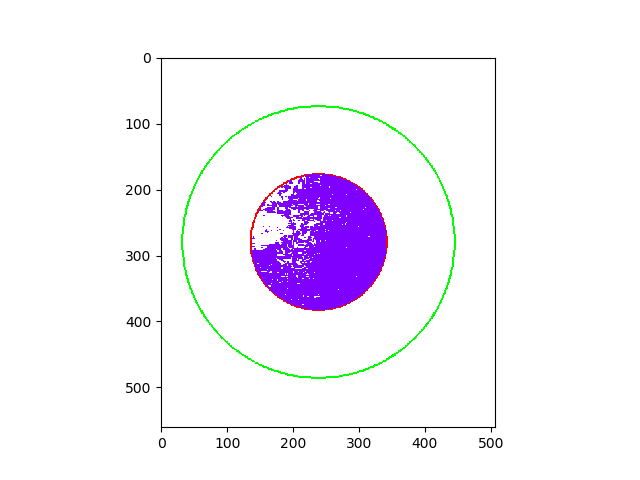

Supplement: Supplementary file 5 — Supplementary Information 5 A dataset of crystal violet staining experiment including raw images, software code and analysis results. [file 41598_2019_44167_MOESM5_ESM.zip › images/d14_v_2.jpg.2.png]

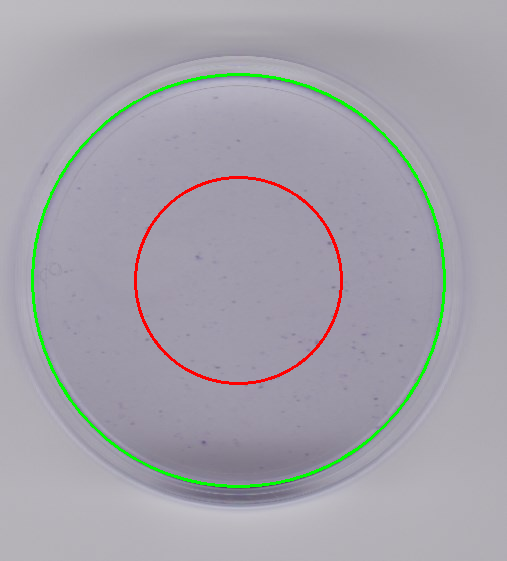

Supplement: Supplementary file 5 — Supplementary Information 5 A dataset of crystal violet staining experiment including raw images, software code and analysis results. [file 41598_2019_44167_MOESM5_ESM.zip › images/d14_v_2.jpg.tiff]

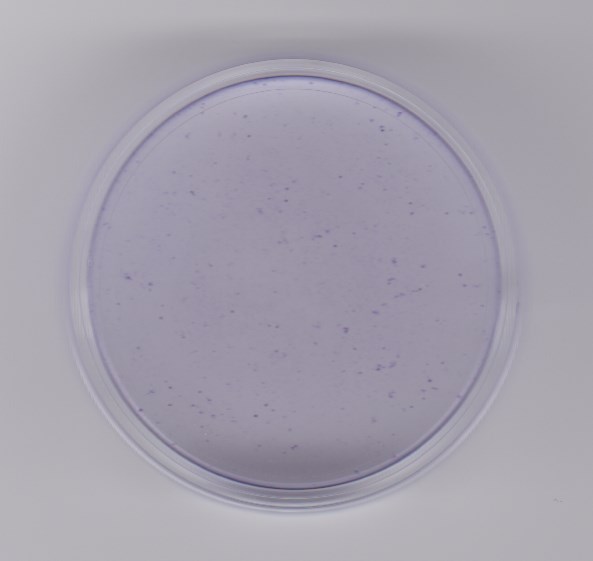

Supplement: Supplementary file 5 — Supplementary Information 5 A dataset of crystal violet staining experiment including raw images, software code and analysis results. [file 41598_2019_44167_MOESM5_ESM.zip › images/d14_v_3.jpg]

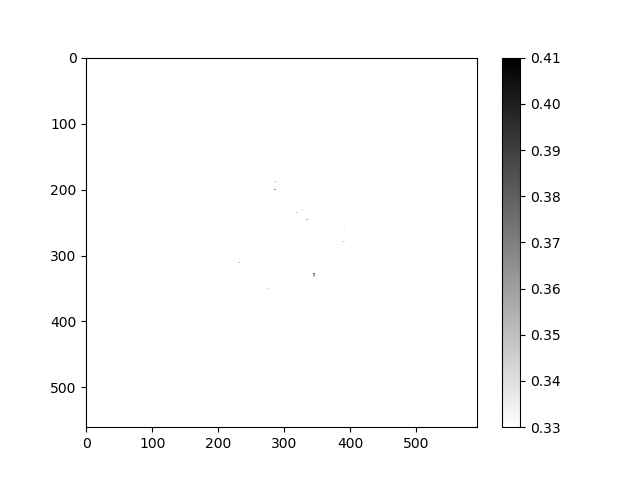

Supplement: Supplementary file 5 — Supplementary Information 5 A dataset of crystal violet staining experiment including raw images, software code and analysis results. [file 41598_2019_44167_MOESM5_ESM.zip › images/d14_v_3.jpg.1.png]

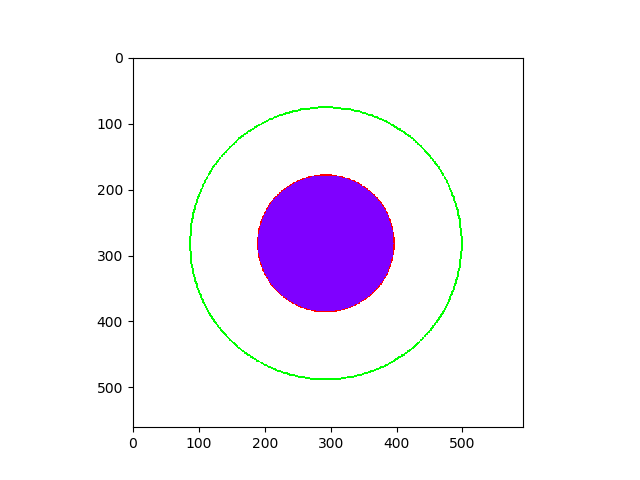

Supplement: Supplementary file 5 — Supplementary Information 5 A dataset of crystal violet staining experiment including raw images, software code and analysis results. [file 41598_2019_44167_MOESM5_ESM.zip › images/d14_v_3.jpg.2.png]

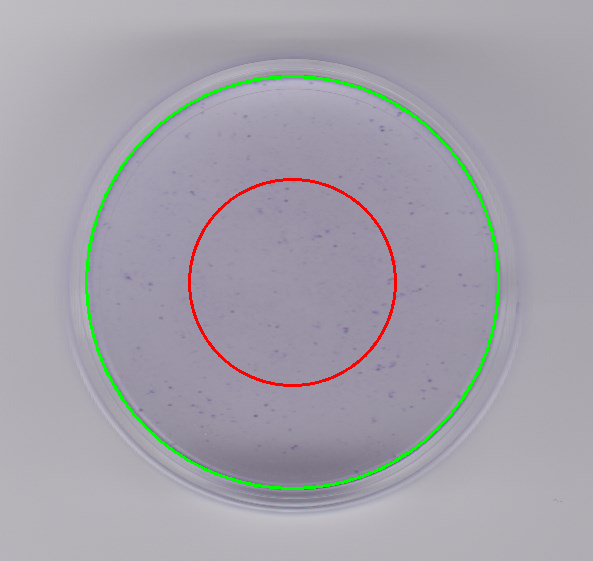

Supplement: Supplementary file 5 — Supplementary Information 5 A dataset of crystal violet staining experiment including raw images, software code and analysis results. [file 41598_2019_44167_MOESM5_ESM.zip › images/d14_v_3.jpg.tiff]

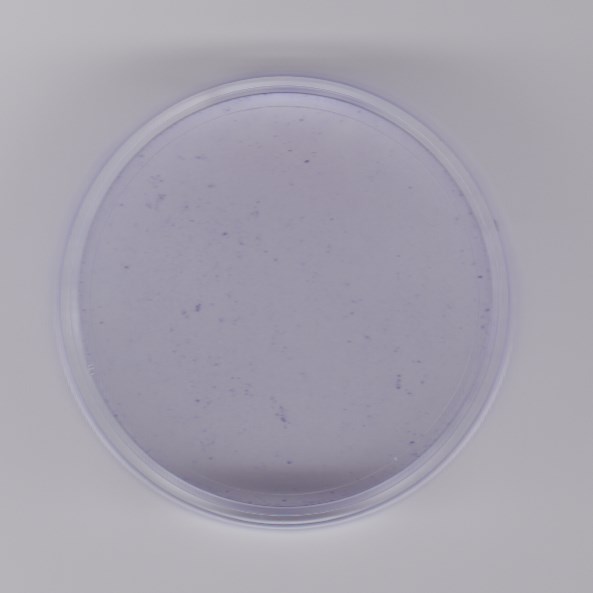

Supplement: Supplementary file 5 — Supplementary Information 5 A dataset of crystal violet staining experiment including raw images, software code and analysis results. [file 41598_2019_44167_MOESM5_ESM.zip › images/d14_v_4.jpg]

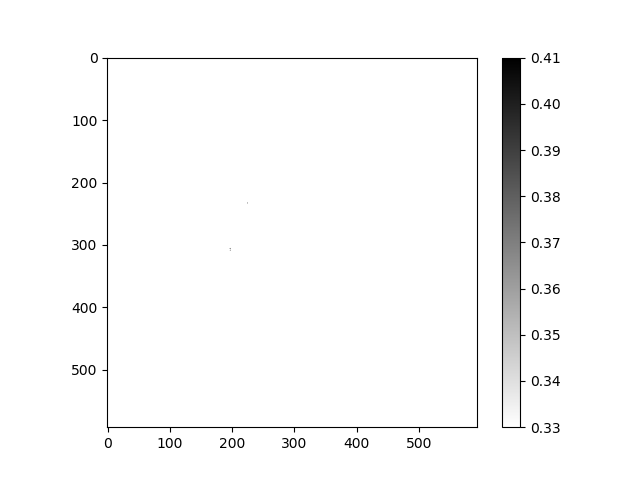

Supplement: Supplementary file 5 — Supplementary Information 5 A dataset of crystal violet staining experiment including raw images, software code and analysis results. [file 41598_2019_44167_MOESM5_ESM.zip › images/d14_v_4.jpg.1.png]

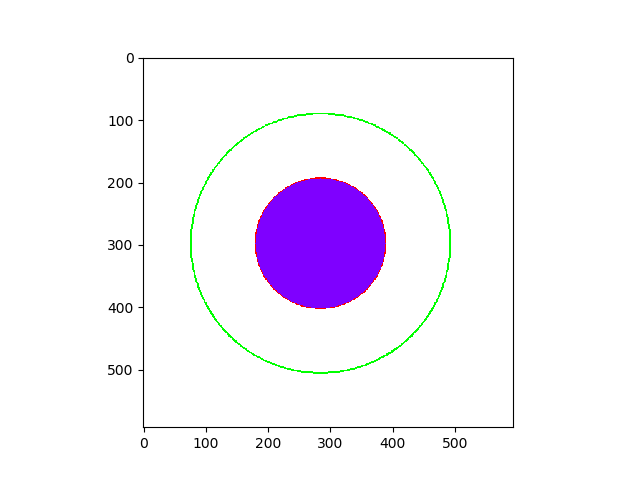

Supplement: Supplementary file 5 — Supplementary Information 5 A dataset of crystal violet staining experiment including raw images, software code and analysis results. [file 41598_2019_44167_MOESM5_ESM.zip › images/d14_v_4.jpg.2.png]

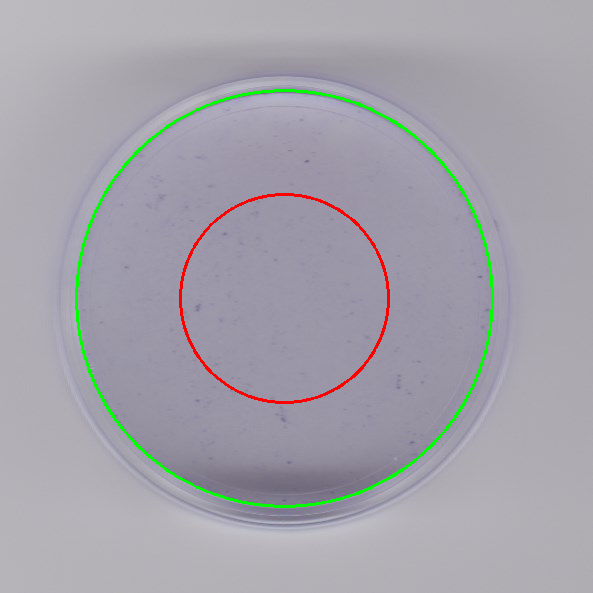

Supplement: Supplementary file 5 — Supplementary Information 5 A dataset of crystal violet staining experiment including raw images, software code and analysis results. [file 41598_2019_44167_MOESM5_ESM.zip › images/d14_v_4.jpg.tiff]

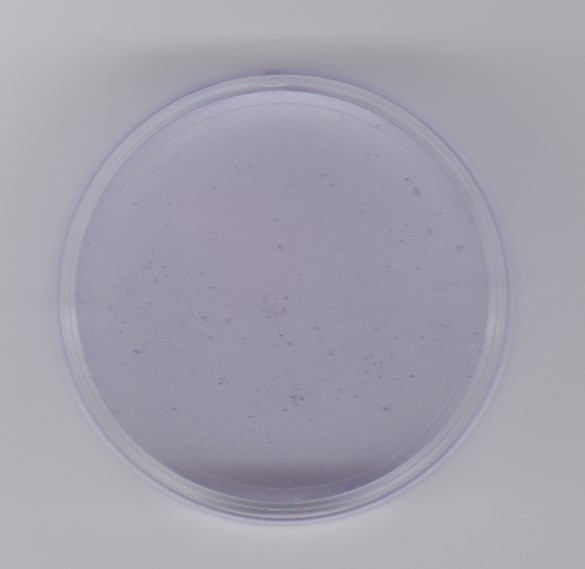

Supplement: Supplementary file 5 — Supplementary Information 5 A dataset of crystal violet staining experiment including raw images, software code and analysis results. [file 41598_2019_44167_MOESM5_ESM.zip › images/d14_v_5.jpg]

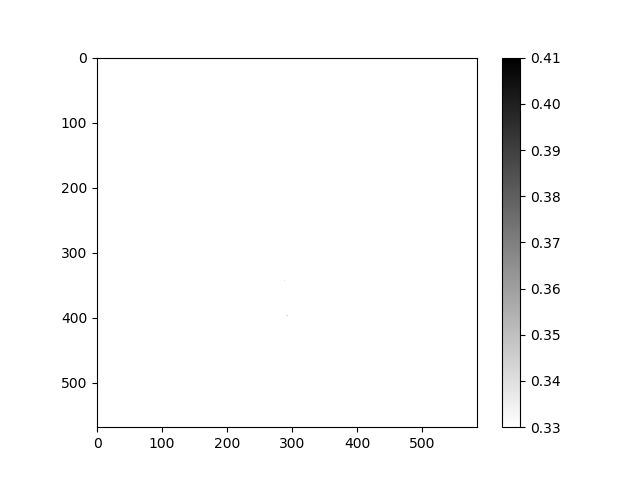

Supplement: Supplementary file 5 — Supplementary Information 5 A dataset of crystal violet staining experiment including raw images, software code and analysis results. [file 41598_2019_44167_MOESM5_ESM.zip › images/d14_v_5.jpg.1.png]

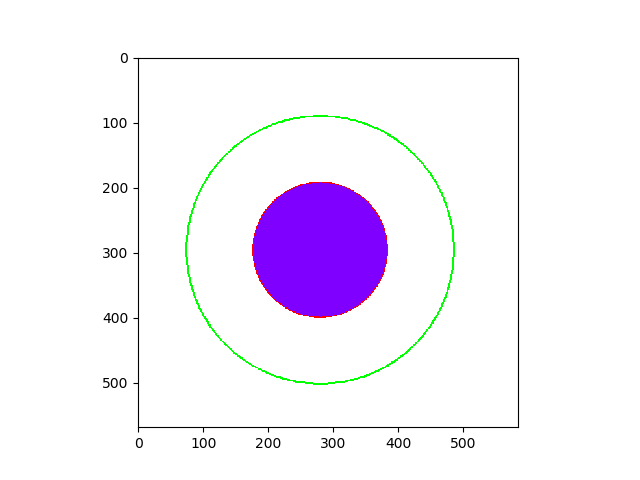

Supplement: Supplementary file 5 — Supplementary Information 5 A dataset of crystal violet staining experiment including raw images, software code and analysis results. [file 41598_2019_44167_MOESM5_ESM.zip › images/d14_v_5.jpg.2.png]

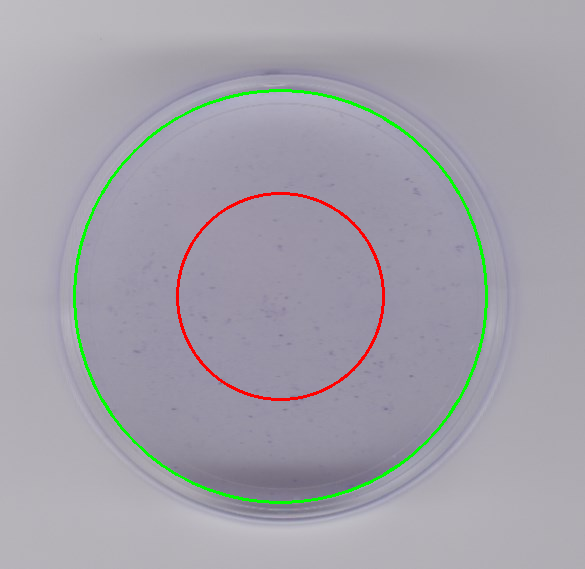

Supplement: Supplementary file 5 — Supplementary Information 5 A dataset of crystal violet staining experiment including raw images, software code and analysis results. [file 41598_2019_44167_MOESM5_ESM.zip › images/d14_v_5.jpg.tiff]

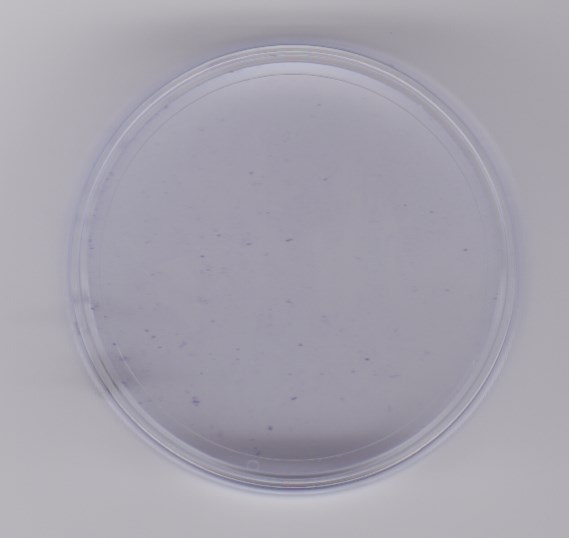

Supplement: Supplementary file 5 — Supplementary Information 5 A dataset of crystal violet staining experiment including raw images, software code and analysis results. [file 41598_2019_44167_MOESM5_ESM.zip › images/d21_p_1.jpg]

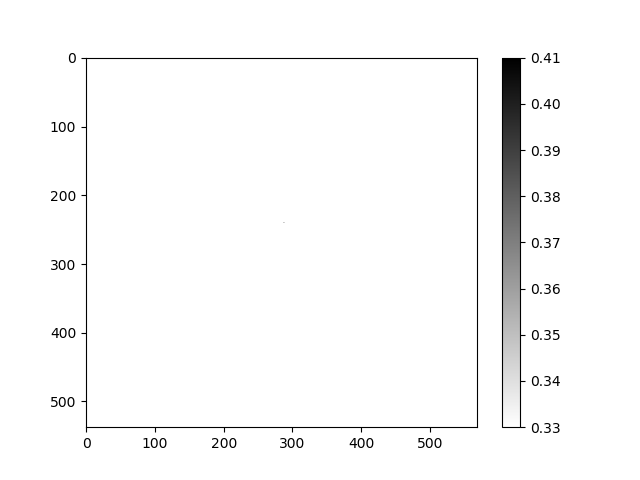

Supplement: Supplementary file 5 — Supplementary Information 5 A dataset of crystal violet staining experiment including raw images, software code and analysis results. [file 41598_2019_44167_MOESM5_ESM.zip › images/d21_p_1.jpg.1.png]

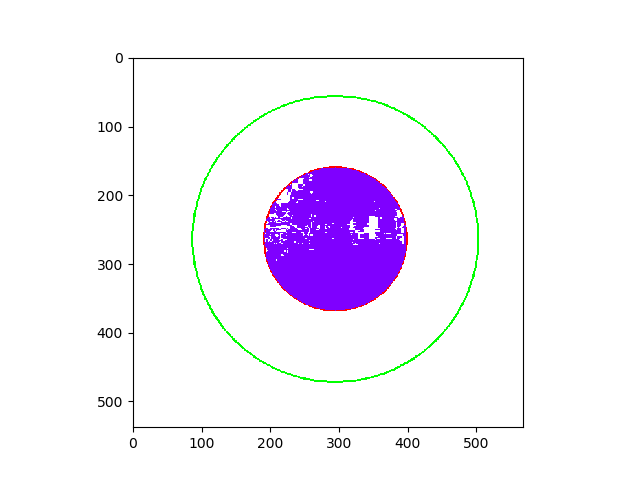

Supplement: Supplementary file 5 — Supplementary Information 5 A dataset of crystal violet staining experiment including raw images, software code and analysis results. [file 41598_2019_44167_MOESM5_ESM.zip › images/d21_p_1.jpg.2.png]

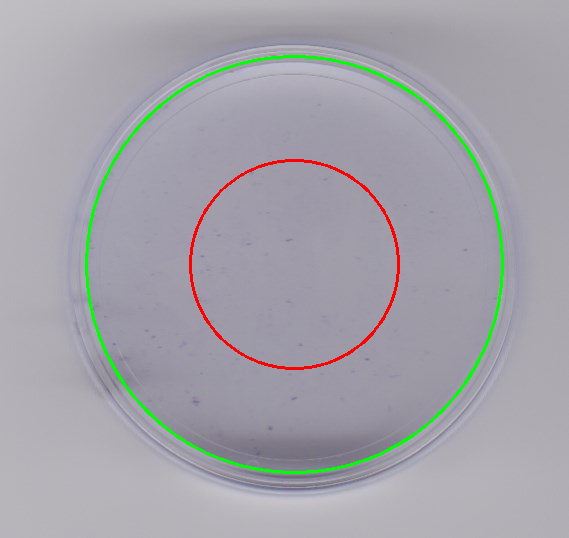

Supplement: Supplementary file 5 — Supplementary Information 5 A dataset of crystal violet staining experiment including raw images, software code and analysis results. [file 41598_2019_44167_MOESM5_ESM.zip › images/d21_p_1.jpg.tiff]

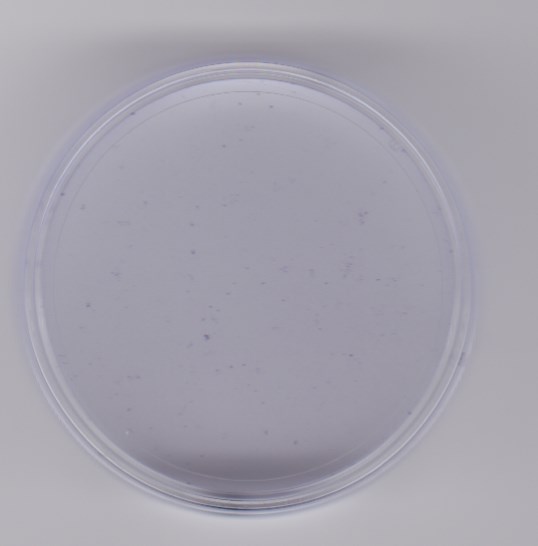

Supplement: Supplementary file 5 — Supplementary Information 5 A dataset of crystal violet staining experiment including raw images, software code and analysis results. [file 41598_2019_44167_MOESM5_ESM.zip › images/d21_p_2.jpg]

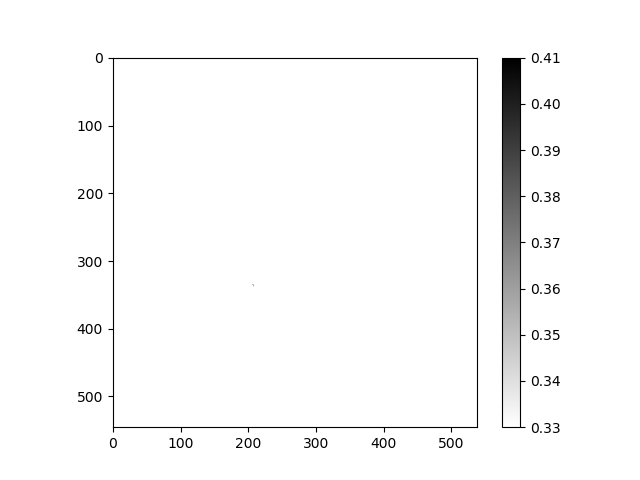

Supplement: Supplementary file 5 — Supplementary Information 5 A dataset of crystal violet staining experiment including raw images, software code and analysis results. [file 41598_2019_44167_MOESM5_ESM.zip › images/d21_p_2.jpg.1.png]

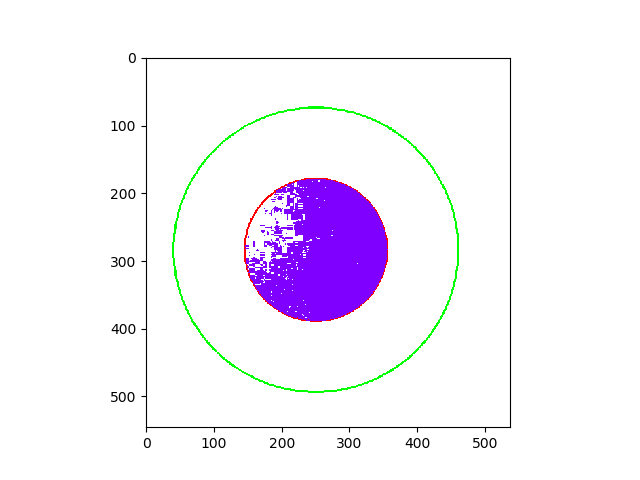

Supplement: Supplementary file 5 — Supplementary Information 5 A dataset of crystal violet staining experiment including raw images, software code and analysis results. [file 41598_2019_44167_MOESM5_ESM.zip › images/d21_p_2.jpg.2.png]

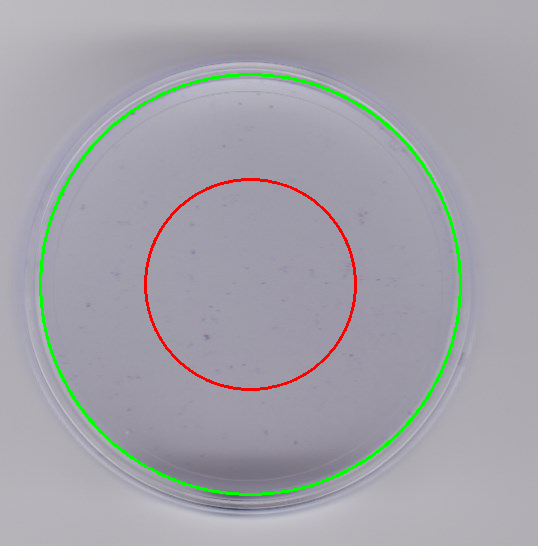

Supplement: Supplementary file 5 — Supplementary Information 5 A dataset of crystal violet staining experiment including raw images, software code and analysis results. [file 41598_2019_44167_MOESM5_ESM.zip › images/d21_p_2.jpg.tiff]

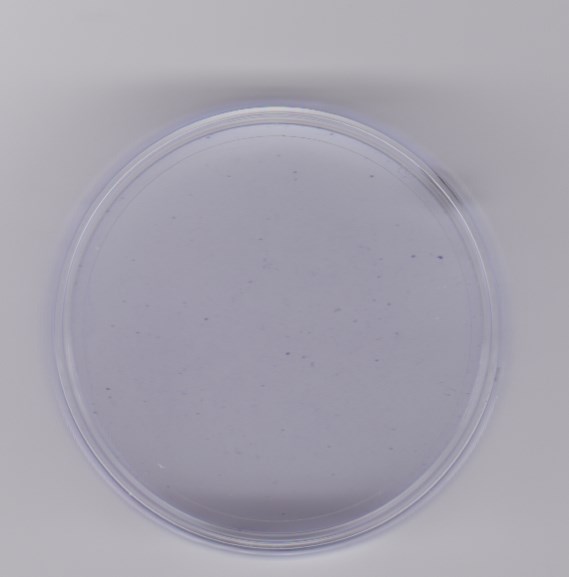

Supplement: Supplementary file 5 — Supplementary Information 5 A dataset of crystal violet staining experiment including raw images, software code and analysis results. [file 41598_2019_44167_MOESM5_ESM.zip › images/d21_p_3.jpg]

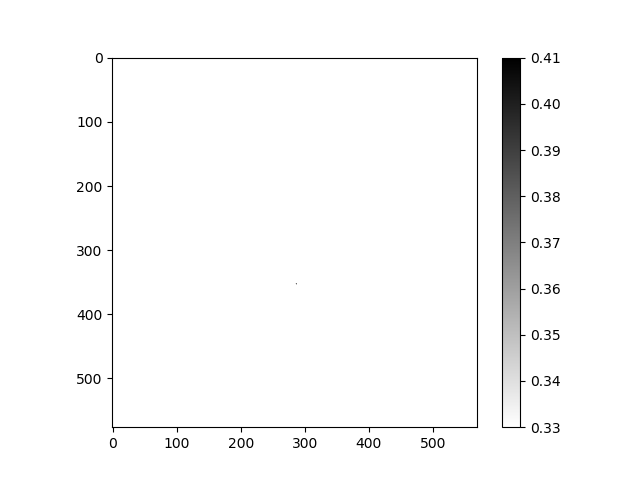

Supplement: Supplementary file 5 — Supplementary Information 5 A dataset of crystal violet staining experiment including raw images, software code and analysis results. [file 41598_2019_44167_MOESM5_ESM.zip › images/d21_p_3.jpg.1.png]

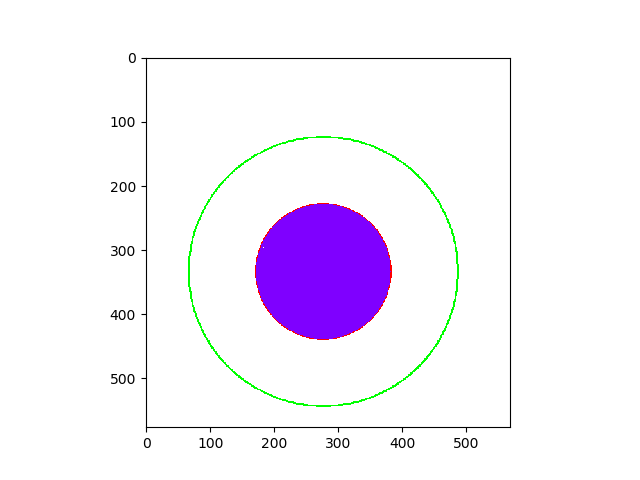

Supplement: Supplementary file 5 — Supplementary Information 5 A dataset of crystal violet staining experiment including raw images, software code and analysis results. [file 41598_2019_44167_MOESM5_ESM.zip › images/d21_p_3.jpg.2.png]

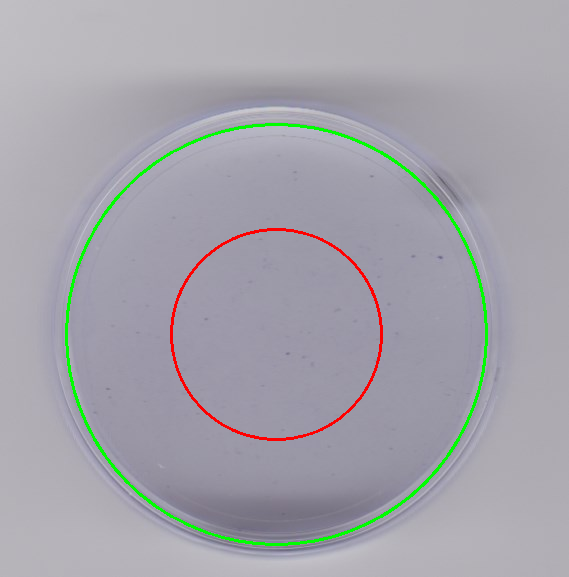

Supplement: Supplementary file 5 — Supplementary Information 5 A dataset of crystal violet staining experiment including raw images, software code and analysis results. [file 41598_2019_44167_MOESM5_ESM.zip › images/d21_p_3.jpg.tiff]

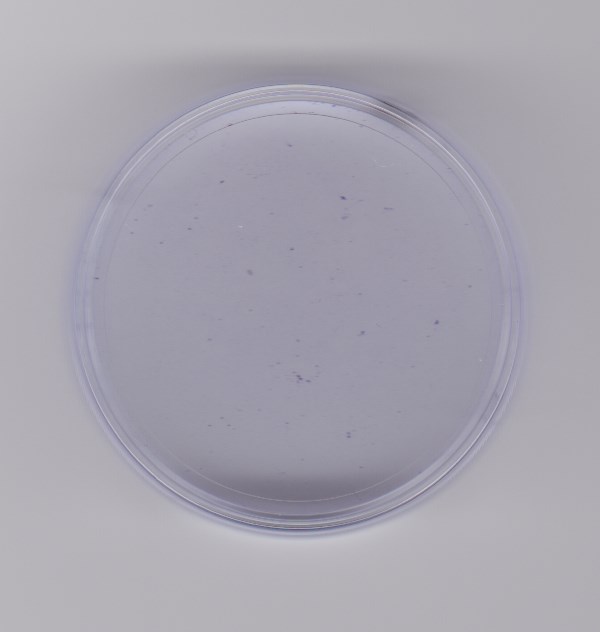

Supplement: Supplementary file 5 — Supplementary Information 5 A dataset of crystal violet staining experiment including raw images, software code and analysis results. [file 41598_2019_44167_MOESM5_ESM.zip › images/d21_p_4.jpg]

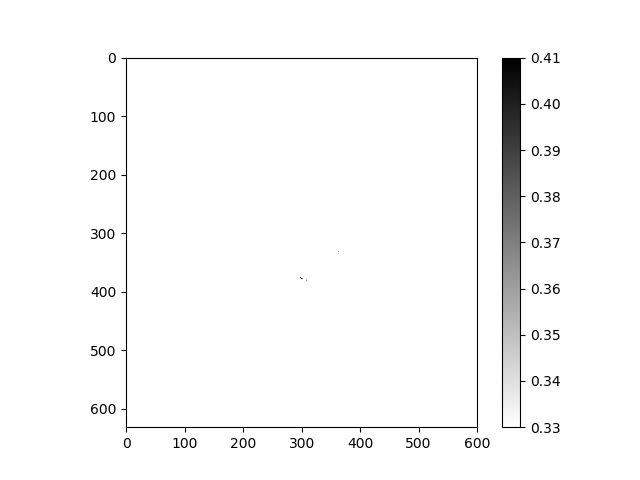

Supplement: Supplementary file 5 — Supplementary Information 5 A dataset of crystal violet staining experiment including raw images, software code and analysis results. [file 41598_2019_44167_MOESM5_ESM.zip › images/d21_p_4.jpg.1.png]

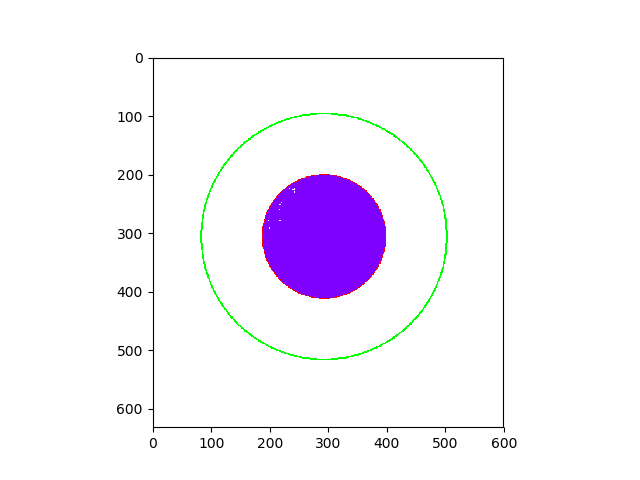

Supplement: Supplementary file 5 — Supplementary Information 5 A dataset of crystal violet staining experiment including raw images, software code and analysis results. [file 41598_2019_44167_MOESM5_ESM.zip › images/d21_p_4.jpg.2.png]

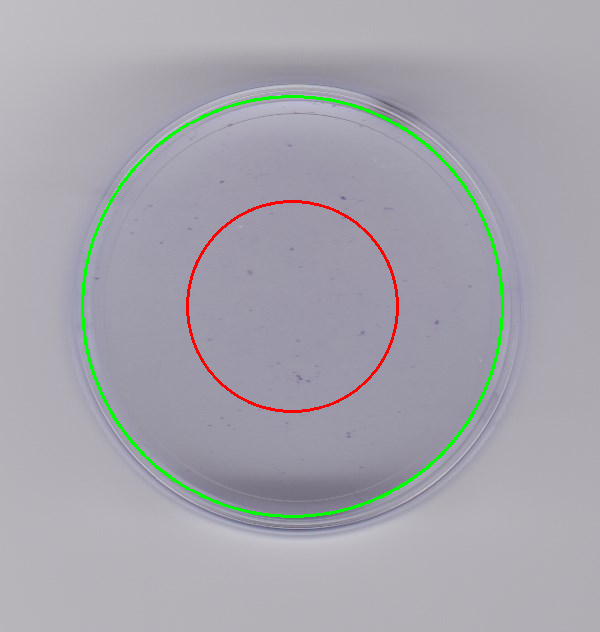

Supplement: Supplementary file 5 — Supplementary Information 5 A dataset of crystal violet staining experiment including raw images, software code and analysis results. [file 41598_2019_44167_MOESM5_ESM.zip › images/d21_p_4.jpg.tiff]

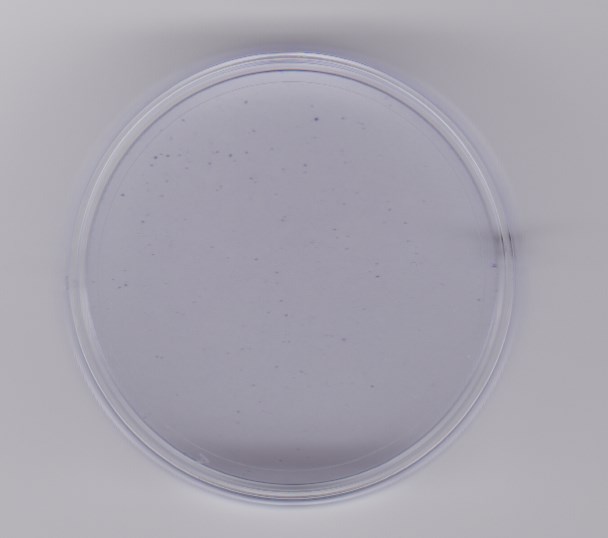

Supplement: Supplementary file 5 — Supplementary Information 5 A dataset of crystal violet staining experiment including raw images, software code and analysis results. [file 41598_2019_44167_MOESM5_ESM.zip › images/d21_p_5.jpg]

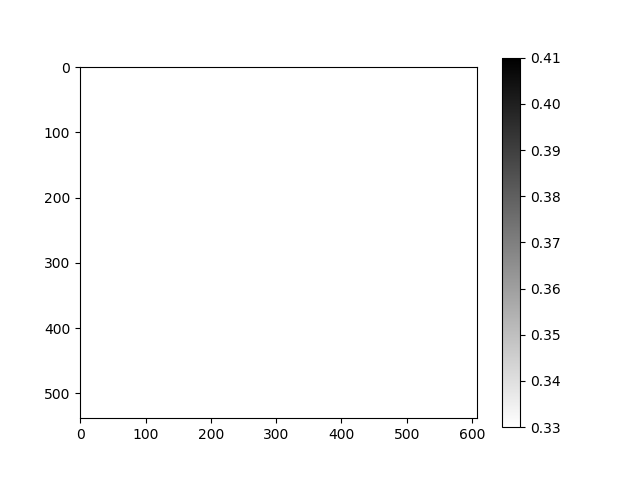

Supplement: Supplementary file 5 — Supplementary Information 5 A dataset of crystal violet staining experiment including raw images, software code and analysis results. [file 41598_2019_44167_MOESM5_ESM.zip › images/d21_p_5.jpg.1.png]

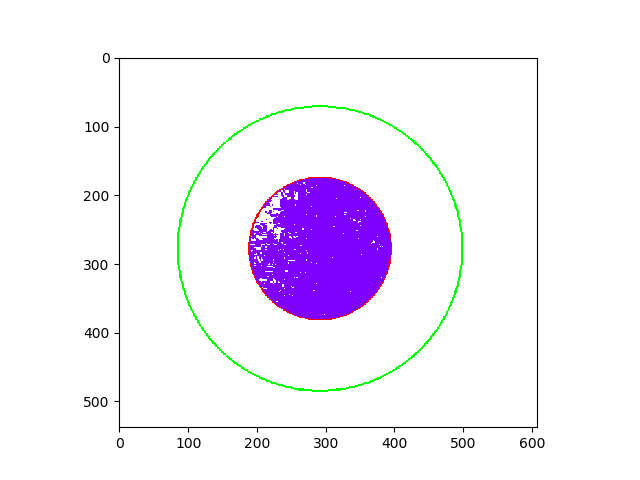

Supplement: Supplementary file 5 — Supplementary Information 5 A dataset of crystal violet staining experiment including raw images, software code and analysis results. [file 41598_2019_44167_MOESM5_ESM.zip › images/d21_p_5.jpg.2.png]

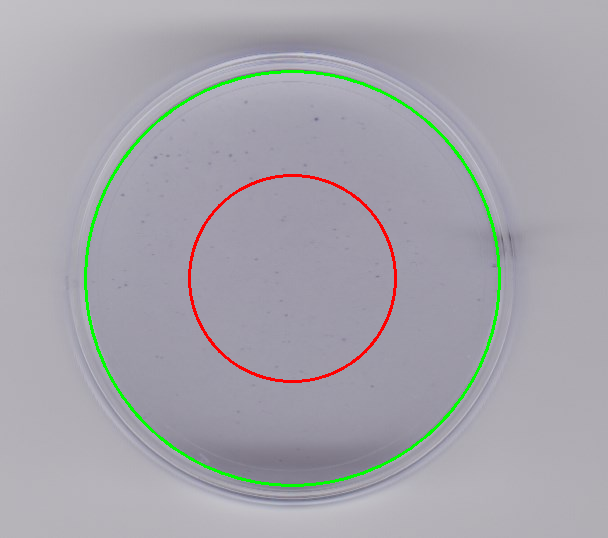

Supplement: Supplementary file 5 — Supplementary Information 5 A dataset of crystal violet staining experiment including raw images, software code and analysis results. [file 41598_2019_44167_MOESM5_ESM.zip › images/d21_p_5.jpg.tiff]

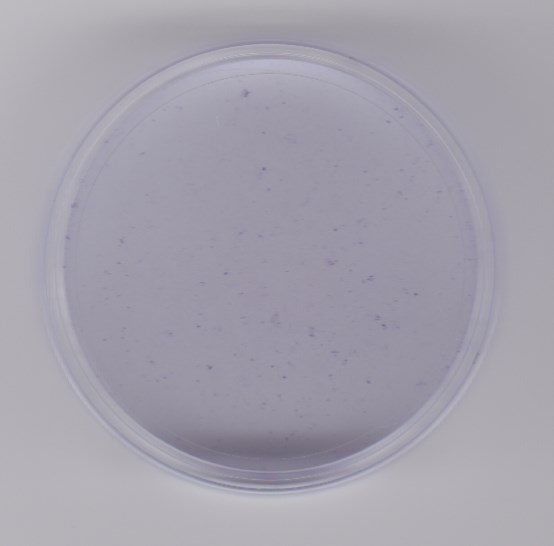

Supplement: Supplementary file 5 — Supplementary Information 5 A dataset of crystal violet staining experiment including raw images, software code and analysis results. [file 41598_2019_44167_MOESM5_ESM.zip › images/d21_v_1.jpg]

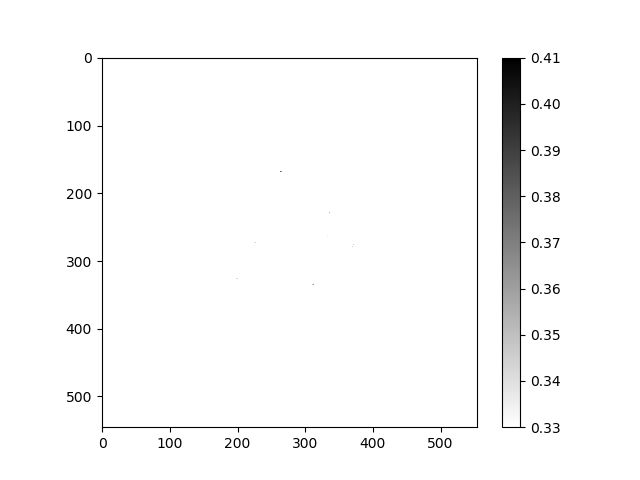

Supplement: Supplementary file 5 — Supplementary Information 5 A dataset of crystal violet staining experiment including raw images, software code and analysis results. [file 41598_2019_44167_MOESM5_ESM.zip › images/d21_v_1.jpg.1.png]

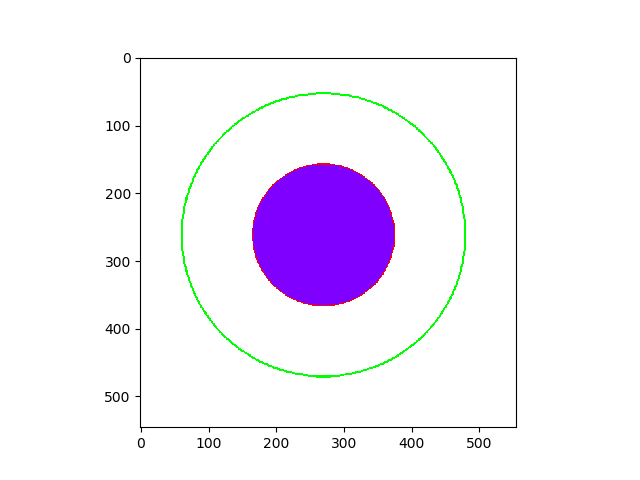

Supplement: Supplementary file 5 — Supplementary Information 5 A dataset of crystal violet staining experiment including raw images, software code and analysis results. [file 41598_2019_44167_MOESM5_ESM.zip › images/d21_v_1.jpg.2.png]

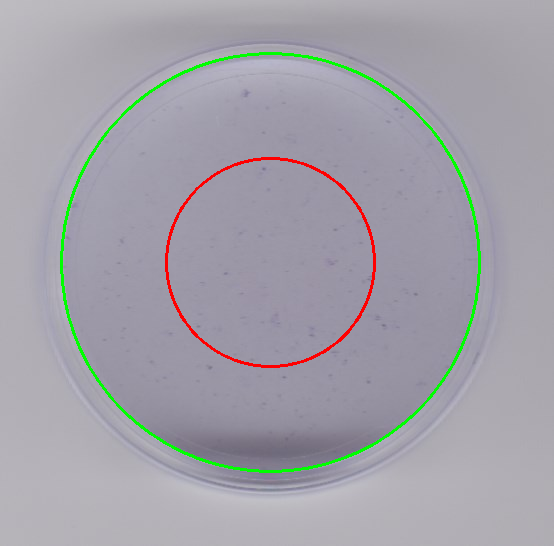

Supplement: Supplementary file 5 — Supplementary Information 5 A dataset of crystal violet staining experiment including raw images, software code and analysis results. [file 41598_2019_44167_MOESM5_ESM.zip › images/d21_v_1.jpg.tiff]

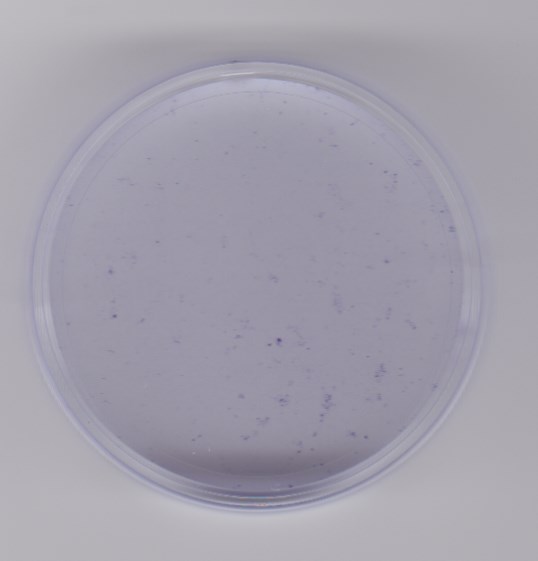

Supplement: Supplementary file 5 — Supplementary Information 5 A dataset of crystal violet staining experiment including raw images, software code and analysis results. [file 41598_2019_44167_MOESM5_ESM.zip › images/d21_v_2.jpg]

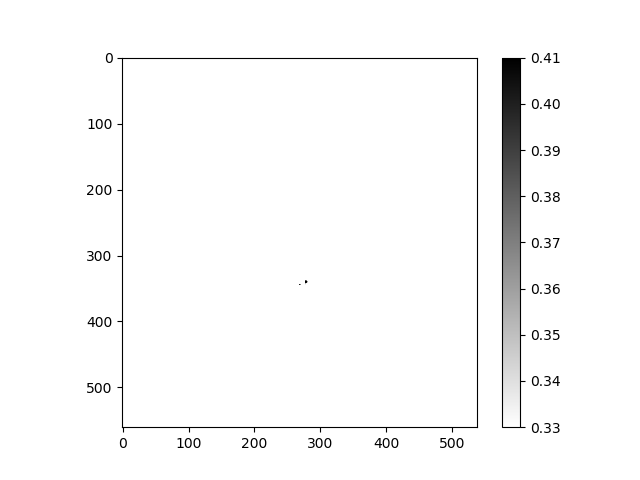

Supplement: Supplementary file 5 — Supplementary Information 5 A dataset of crystal violet staining experiment including raw images, software code and analysis results. [file 41598_2019_44167_MOESM5_ESM.zip › images/d21_v_2.jpg.1.png]

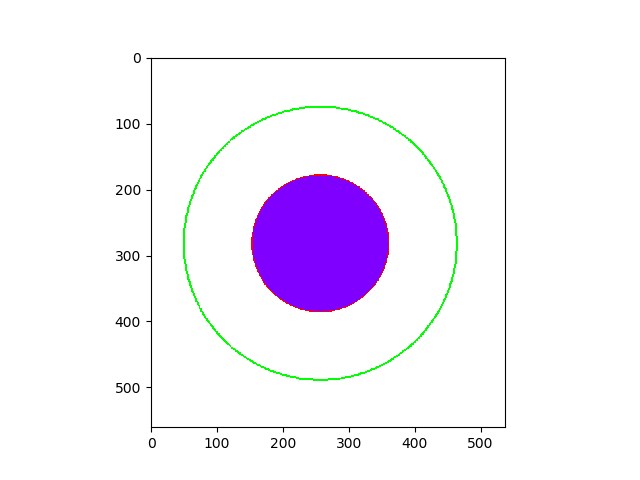

Supplement: Supplementary file 5 — Supplementary Information 5 A dataset of crystal violet staining experiment including raw images, software code and analysis results. [file 41598_2019_44167_MOESM5_ESM.zip › images/d21_v_2.jpg.2.png]

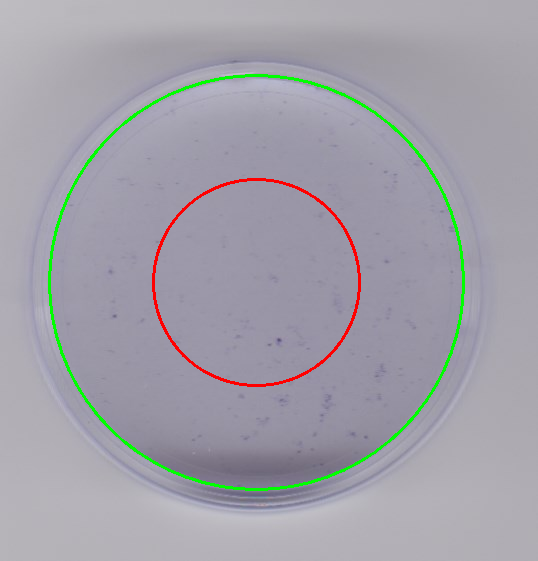

Supplement: Supplementary file 5 — Supplementary Information 5 A dataset of crystal violet staining experiment including raw images, software code and analysis results. [file 41598_2019_44167_MOESM5_ESM.zip › images/d21_v_2.jpg.tiff]

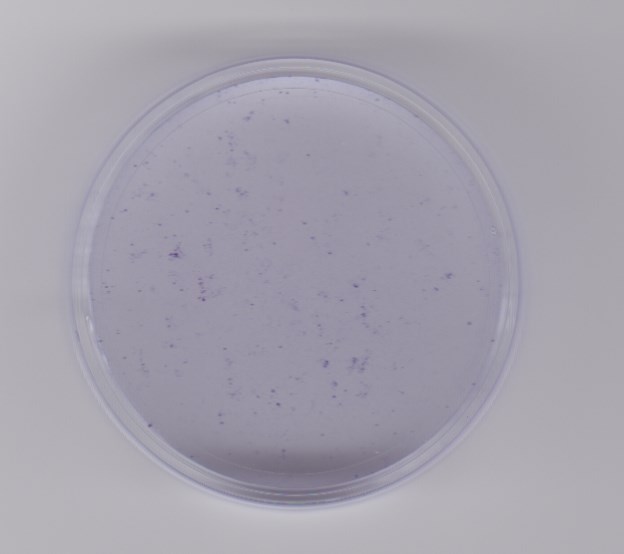

Supplement: Supplementary file 5 — Supplementary Information 5 A dataset of crystal violet staining experiment including raw images, software code and analysis results. [file 41598_2019_44167_MOESM5_ESM.zip › images/d21_v_3.jpg]

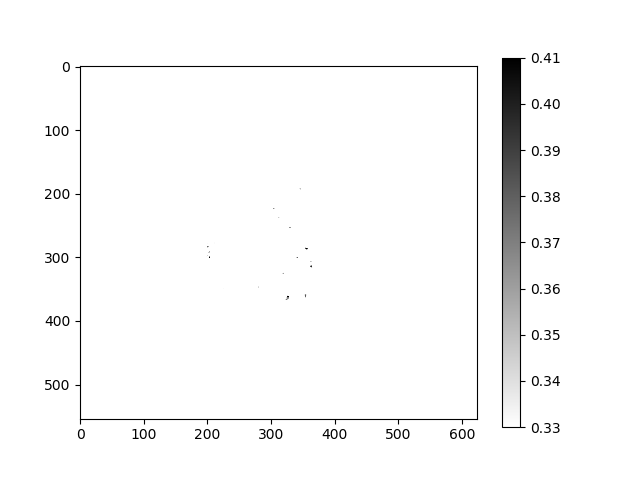

Supplement: Supplementary file 5 — Supplementary Information 5 A dataset of crystal violet staining experiment including raw images, software code and analysis results. [file 41598_2019_44167_MOESM5_ESM.zip › images/d21_v_3.jpg.1.png]

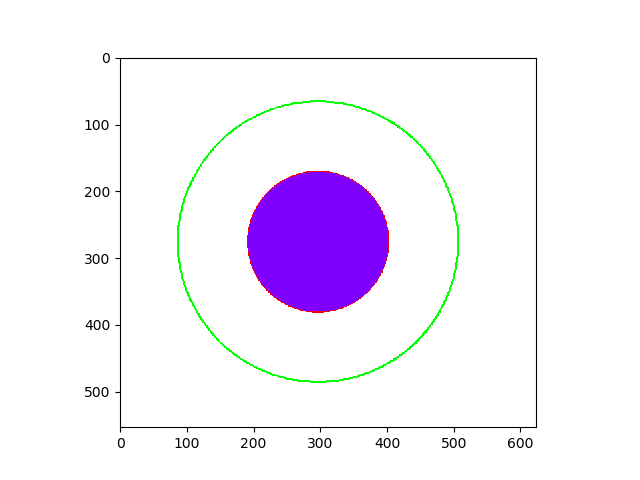

Supplement: Supplementary file 5 — Supplementary Information 5 A dataset of crystal violet staining experiment including raw images, software code and analysis results. [file 41598_2019_44167_MOESM5_ESM.zip › images/d21_v_3.jpg.2.png]

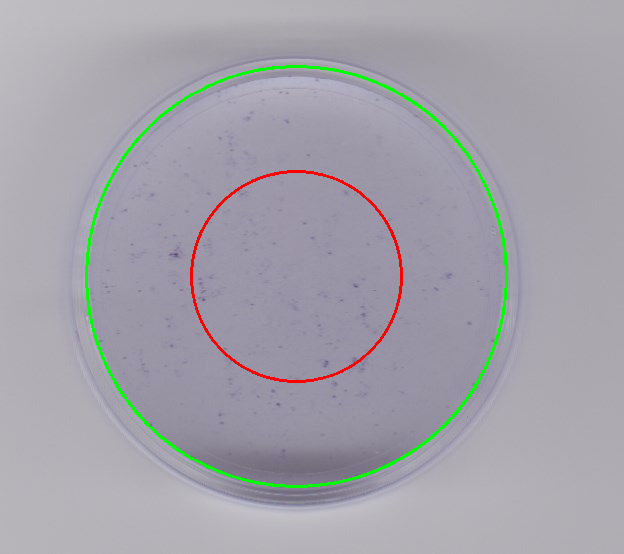

Supplement: Supplementary file 5 — Supplementary Information 5 A dataset of crystal violet staining experiment including raw images, software code and analysis results. [file 41598_2019_44167_MOESM5_ESM.zip › images/d21_v_3.jpg.tiff]

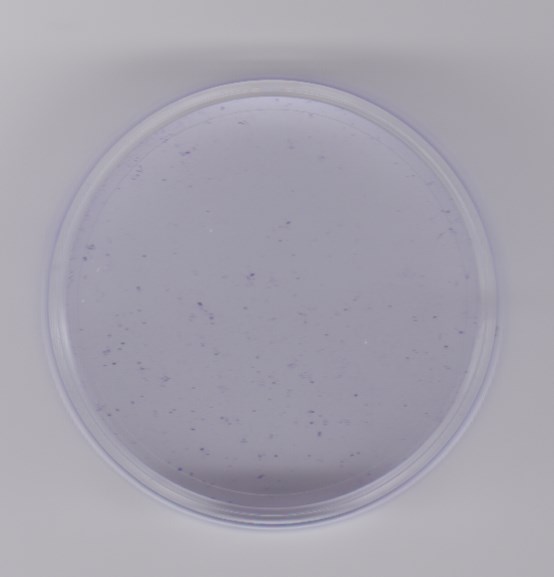

Supplement: Supplementary file 5 — Supplementary Information 5 A dataset of crystal violet staining experiment including raw images, software code and analysis results. [file 41598_2019_44167_MOESM5_ESM.zip › images/d21_v_4.jpg]

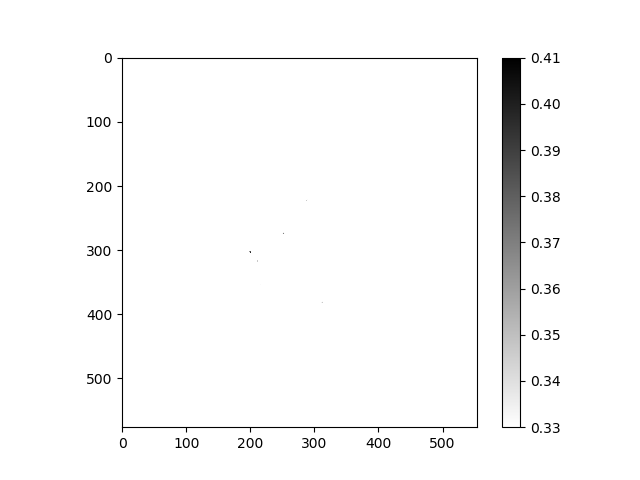

Supplement: Supplementary file 5 — Supplementary Information 5 A dataset of crystal violet staining experiment including raw images, software code and analysis results. [file 41598_2019_44167_MOESM5_ESM.zip › images/d21_v_4.jpg.1.png]

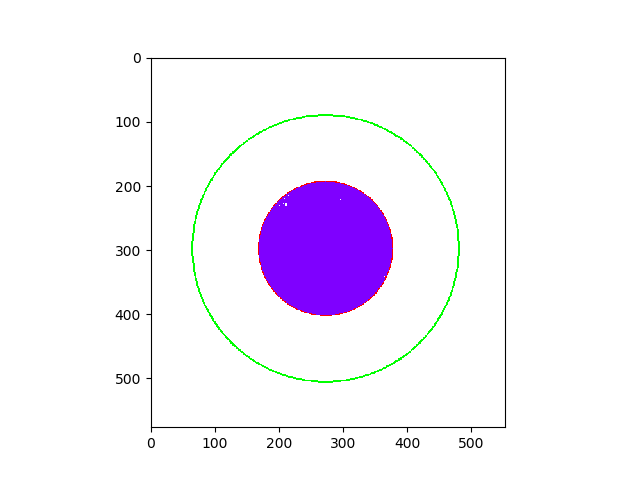

Supplement: Supplementary file 5 — Supplementary Information 5 A dataset of crystal violet staining experiment including raw images, software code and analysis results. [file 41598_2019_44167_MOESM5_ESM.zip › images/d21_v_4.jpg.2.png]

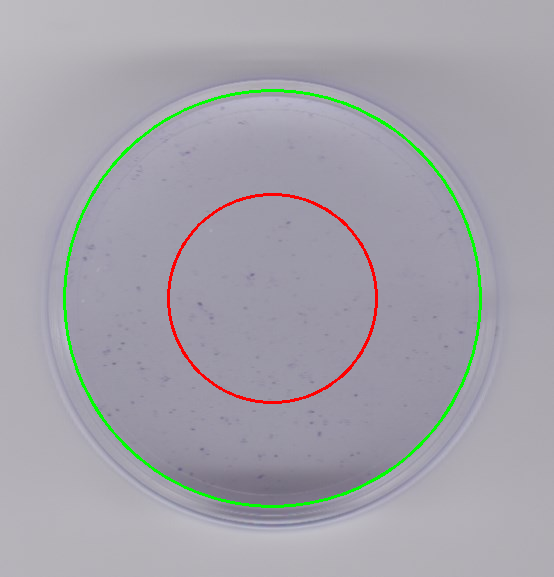

Supplement: Supplementary file 5 — Supplementary Information 5 A dataset of crystal violet staining experiment including raw images, software code and analysis results. [file 41598_2019_44167_MOESM5_ESM.zip › images/d21_v_4.jpg.tiff]

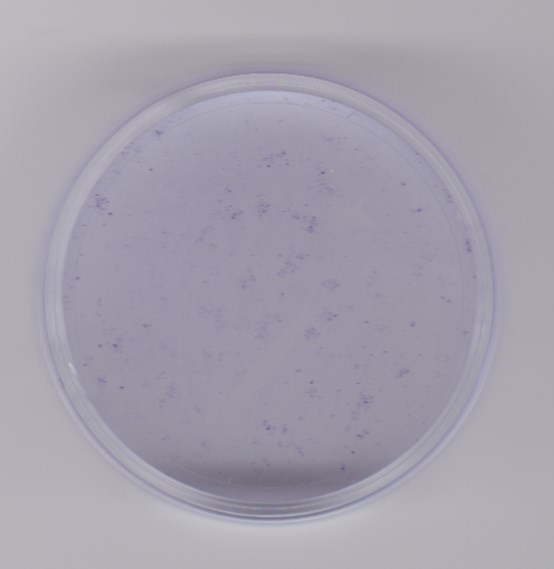

Supplement: Supplementary file 5 — Supplementary Information 5 A dataset of crystal violet staining experiment including raw images, software code and analysis results. [file 41598_2019_44167_MOESM5_ESM.zip › images/d21_v_5.jpg]

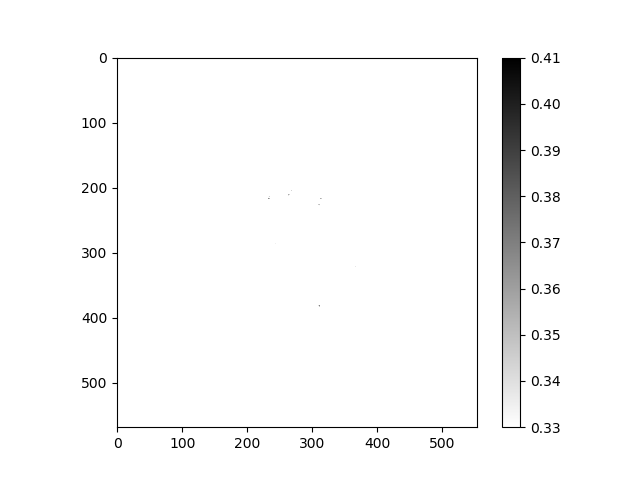

Supplement: Supplementary file 5 — Supplementary Information 5 A dataset of crystal violet staining experiment including raw images, software code and analysis results. [file 41598_2019_44167_MOESM5_ESM.zip › images/d21_v_5.jpg.1.png]

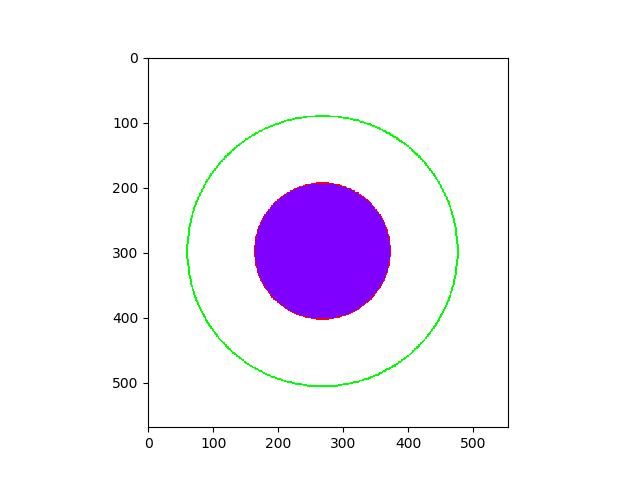

Supplement: Supplementary file 5 — Supplementary Information 5 A dataset of crystal violet staining experiment including raw images, software code and analysis results. [file 41598_2019_44167_MOESM5_ESM.zip › images/d21_v_5.jpg.2.png]

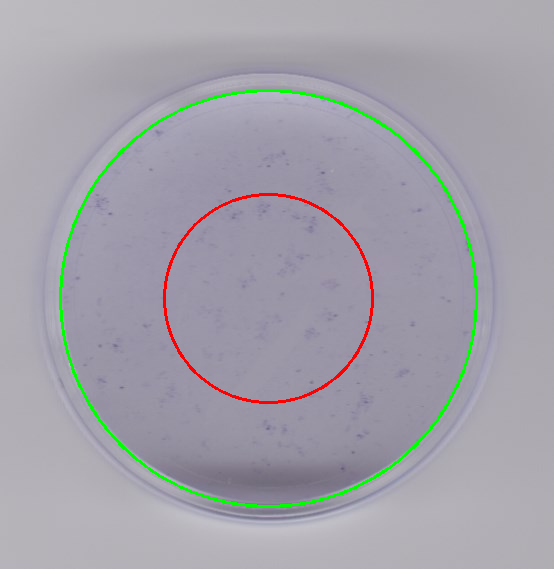

Supplement: Supplementary file 5 — Supplementary Information 5 A dataset of crystal violet staining experiment including raw images, software code and analysis results. [file 41598_2019_44167_MOESM5_ESM.zip › images/d21_v_5.jpg.tiff]

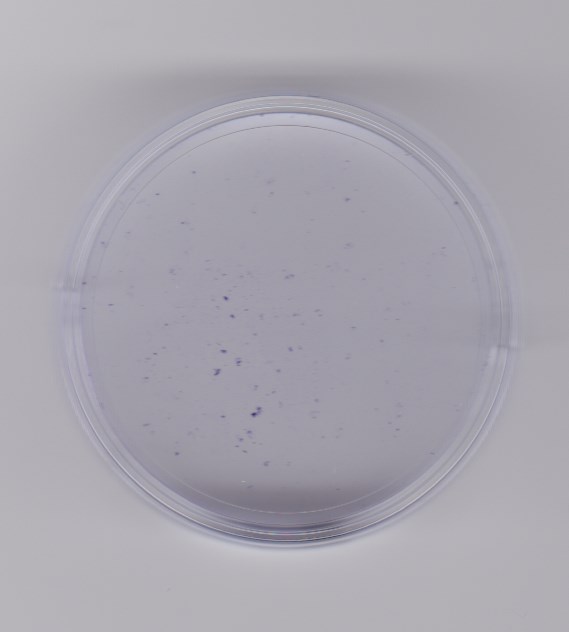

Supplement: Supplementary file 5 — Supplementary Information 5 A dataset of crystal violet staining experiment including raw images, software code and analysis results. [file 41598_2019_44167_MOESM5_ESM.zip › images/d28_p_1.jpg]
