# Supplementary figures and images for: In-situ detection based on the biofilm hydrophilicity for environmental biofilm formation (part 1 of 2)
Source: Sci Rep. 2019 May 30;9:8070. doi: 10.1038/s41598-019-44167-6 (PMC6542837; doi:10.1038/s41598-019-44167-6)

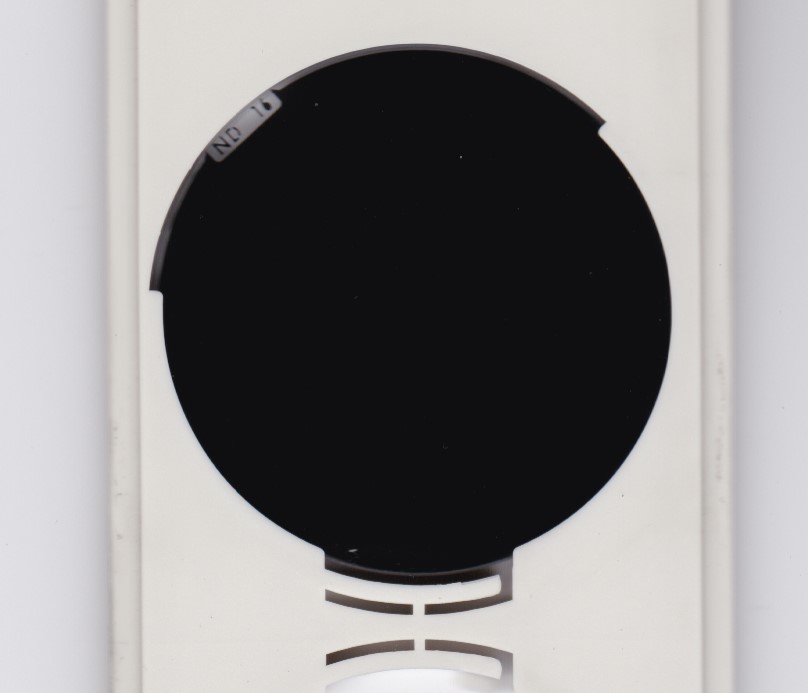

Supplement: Supplementary file 5 — Supplementary Information 5 A dataset of crystal violet staining experiment including raw images, software code and analysis results. [file 41598_2019_44167_MOESM5_ESM.zip › calibration/IMG_20190402_0001.jpg]

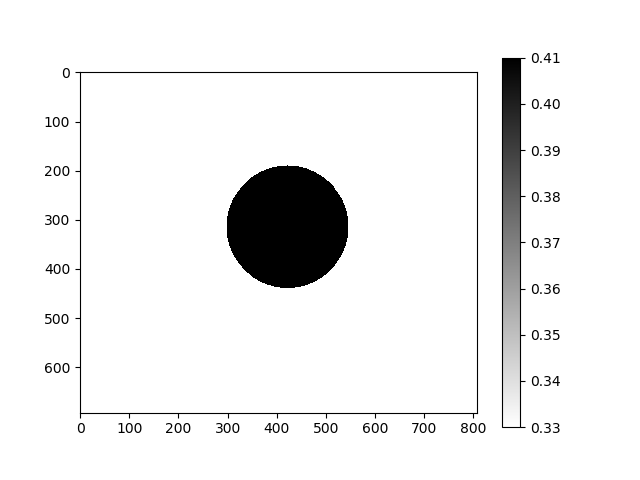

Supplement: Supplementary file 5 — Supplementary Information 5 A dataset of crystal violet staining experiment including raw images, software code and analysis results. [file 41598_2019_44167_MOESM5_ESM.zip › calibration/IMG_20190402_0001.jpg.1.png]

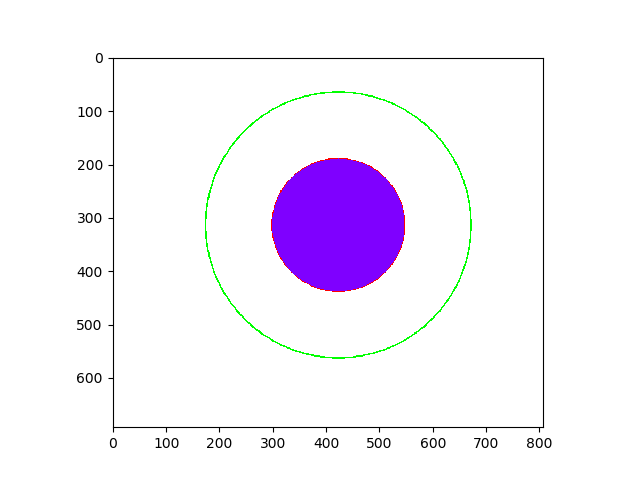

Supplement: Supplementary file 5 — Supplementary Information 5 A dataset of crystal violet staining experiment including raw images, software code and analysis results. [file 41598_2019_44167_MOESM5_ESM.zip › calibration/IMG_20190402_0001.jpg.2.png]

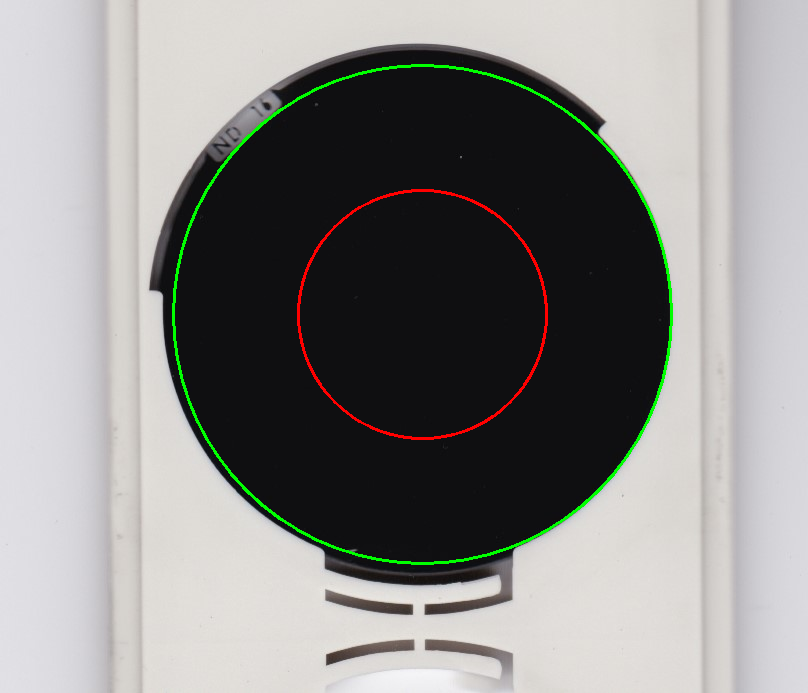

Supplement: Supplementary file 5 — Supplementary Information 5 A dataset of crystal violet staining experiment including raw images, software code and analysis results. [file 41598_2019_44167_MOESM5_ESM.zip › calibration/IMG_20190402_0001.jpg.tiff]

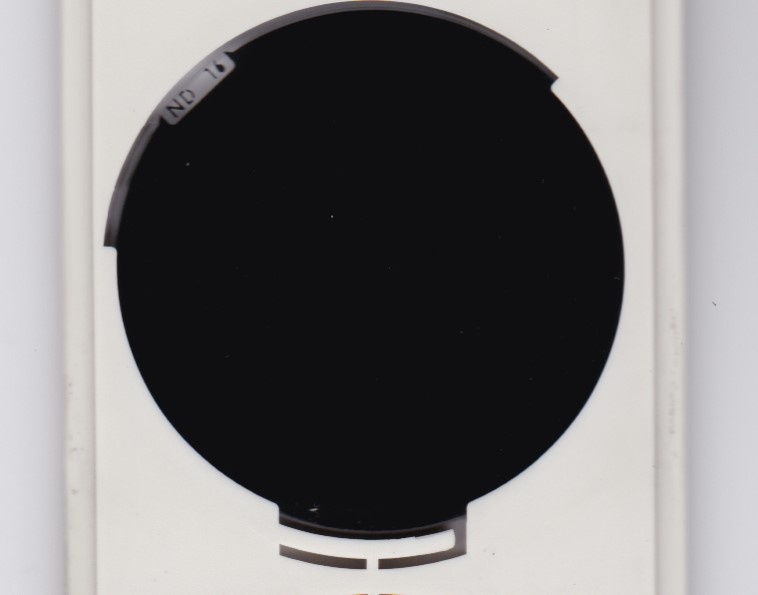

Supplement: Supplementary file 5 — Supplementary Information 5 A dataset of crystal violet staining experiment including raw images, software code and analysis results. [file 41598_2019_44167_MOESM5_ESM.zip › calibration/IMG_20190402_0002.jpg]

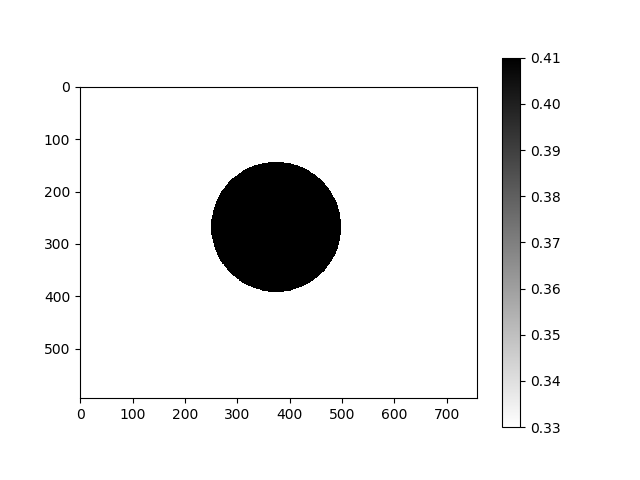

Supplement: Supplementary file 5 — Supplementary Information 5 A dataset of crystal violet staining experiment including raw images, software code and analysis results. [file 41598_2019_44167_MOESM5_ESM.zip › calibration/IMG_20190402_0002.jpg.1.png]

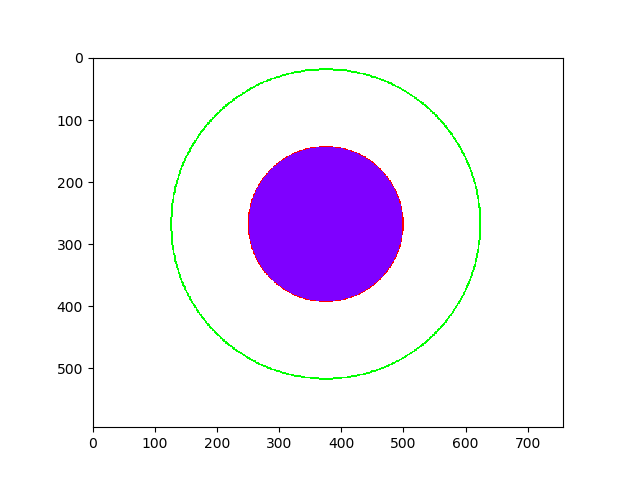

Supplement: Supplementary file 5 — Supplementary Information 5 A dataset of crystal violet staining experiment including raw images, software code and analysis results. [file 41598_2019_44167_MOESM5_ESM.zip › calibration/IMG_20190402_0002.jpg.2.png]

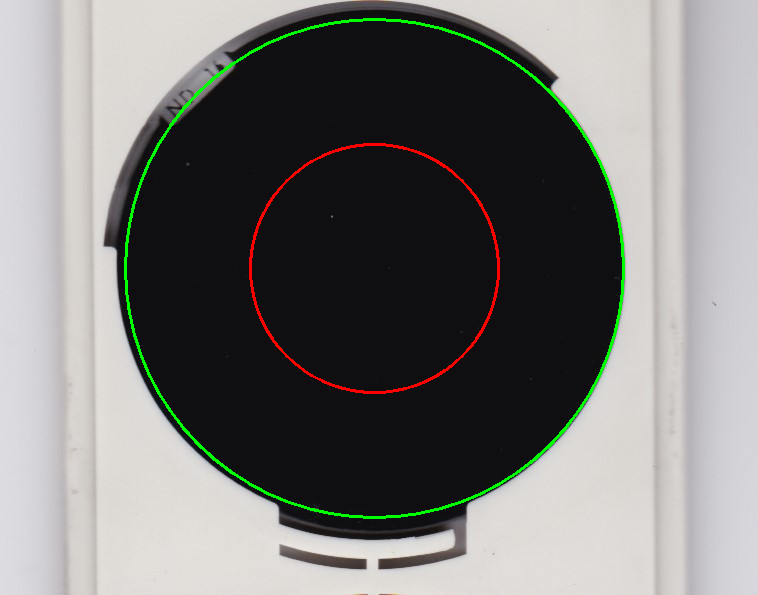

Supplement: Supplementary file 5 — Supplementary Information 5 A dataset of crystal violet staining experiment including raw images, software code and analysis results. [file 41598_2019_44167_MOESM5_ESM.zip › calibration/IMG_20190402_0002.jpg.tiff]

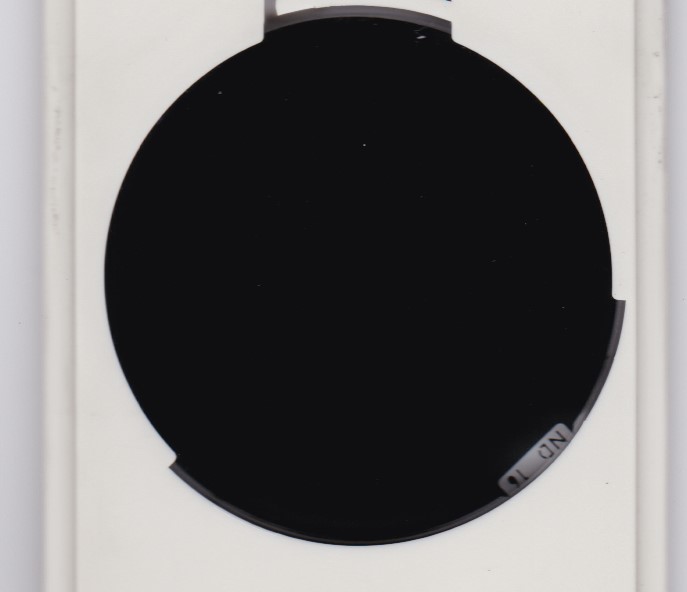

Supplement: Supplementary file 5 — Supplementary Information 5 A dataset of crystal violet staining experiment including raw images, software code and analysis results. [file 41598_2019_44167_MOESM5_ESM.zip › calibration/IMG_20190402_0003.jpg]

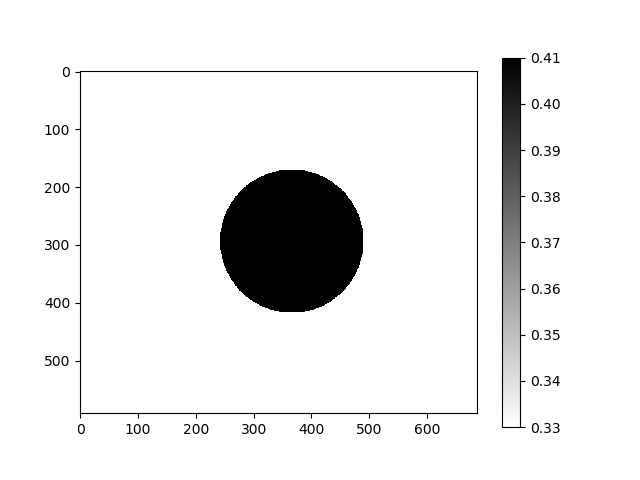

Supplement: Supplementary file 5 — Supplementary Information 5 A dataset of crystal violet staining experiment including raw images, software code and analysis results. [file 41598_2019_44167_MOESM5_ESM.zip › calibration/IMG_20190402_0003.jpg.1.png]

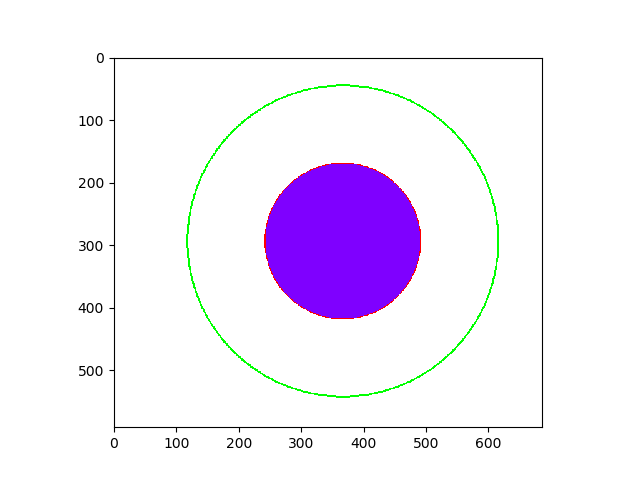

Supplement: Supplementary file 5 — Supplementary Information 5 A dataset of crystal violet staining experiment including raw images, software code and analysis results. [file 41598_2019_44167_MOESM5_ESM.zip › calibration/IMG_20190402_0003.jpg.2.png]

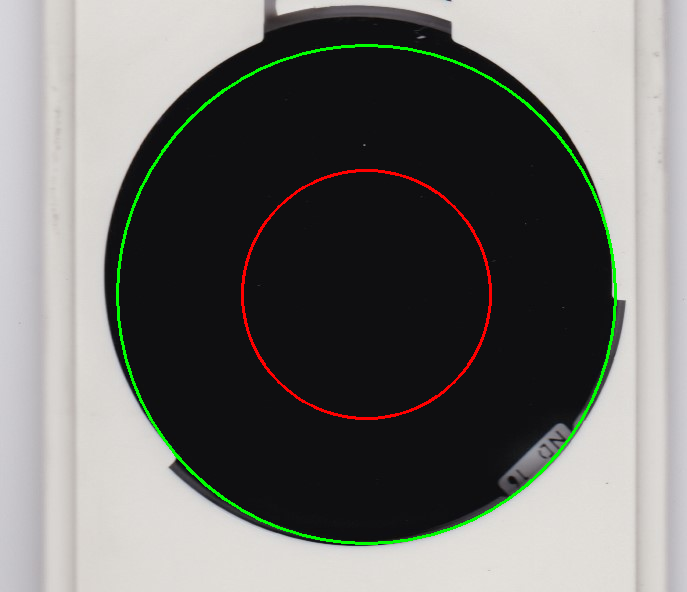

Supplement: Supplementary file 5 — Supplementary Information 5 A dataset of crystal violet staining experiment including raw images, software code and analysis results. [file 41598_2019_44167_MOESM5_ESM.zip › calibration/IMG_20190402_0003.jpg.tiff]

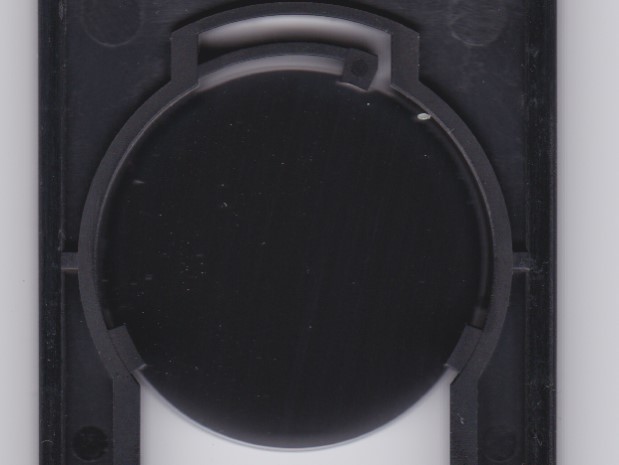

Supplement: Supplementary file 5 — Supplementary Information 5 A dataset of crystal violet staining experiment including raw images, software code and analysis results. [file 41598_2019_44167_MOESM5_ESM.zip › calibration/IMG_20190402_0004.jpg]

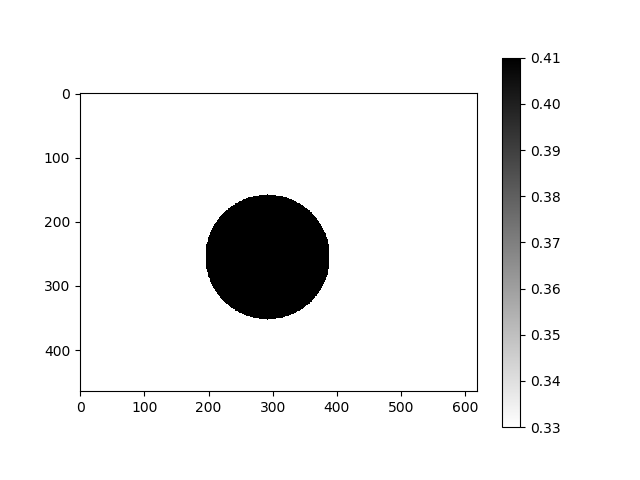

Supplement: Supplementary file 5 — Supplementary Information 5 A dataset of crystal violet staining experiment including raw images, software code and analysis results. [file 41598_2019_44167_MOESM5_ESM.zip › calibration/IMG_20190402_0004.jpg.1.png]

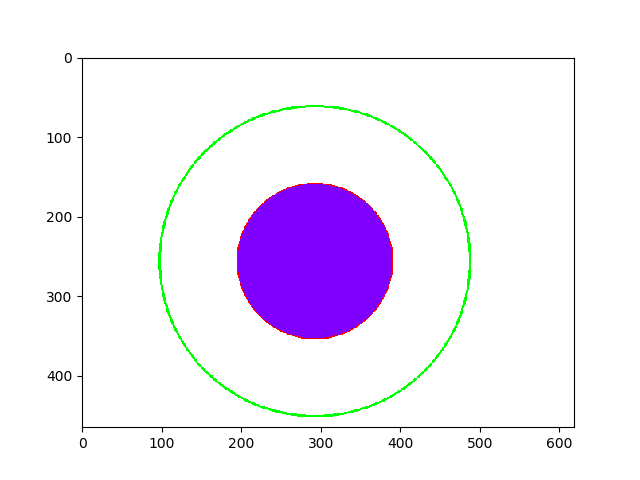

Supplement: Supplementary file 5 — Supplementary Information 5 A dataset of crystal violet staining experiment including raw images, software code and analysis results. [file 41598_2019_44167_MOESM5_ESM.zip › calibration/IMG_20190402_0004.jpg.2.png]

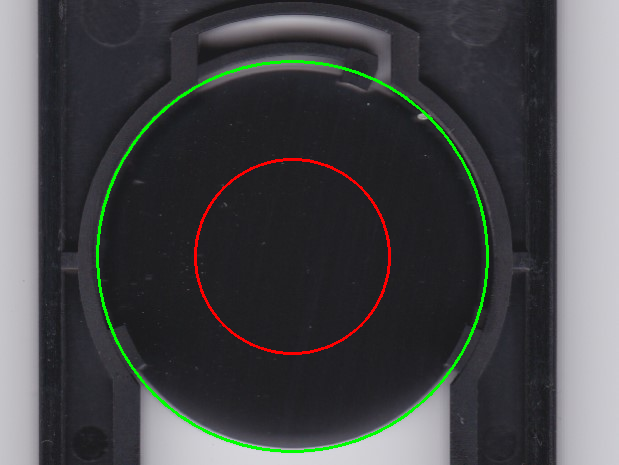

Supplement: Supplementary file 5 — Supplementary Information 5 A dataset of crystal violet staining experiment including raw images, software code and analysis results. [file 41598_2019_44167_MOESM5_ESM.zip › calibration/IMG_20190402_0004.jpg.tiff]

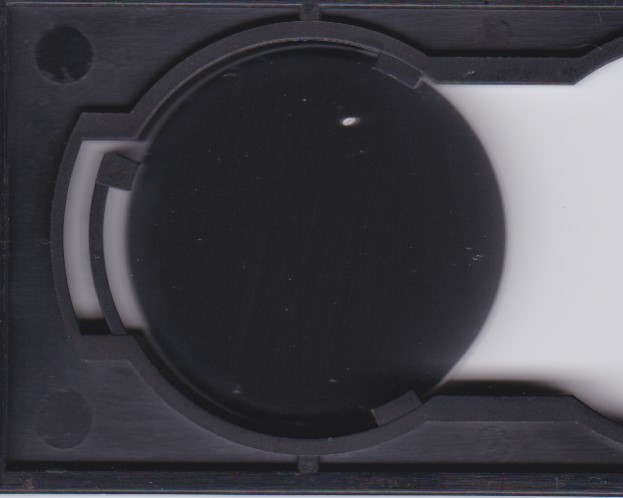

Supplement: Supplementary file 5 — Supplementary Information 5 A dataset of crystal violet staining experiment including raw images, software code and analysis results. [file 41598_2019_44167_MOESM5_ESM.zip › calibration/IMG_20190402_0005.jpg]

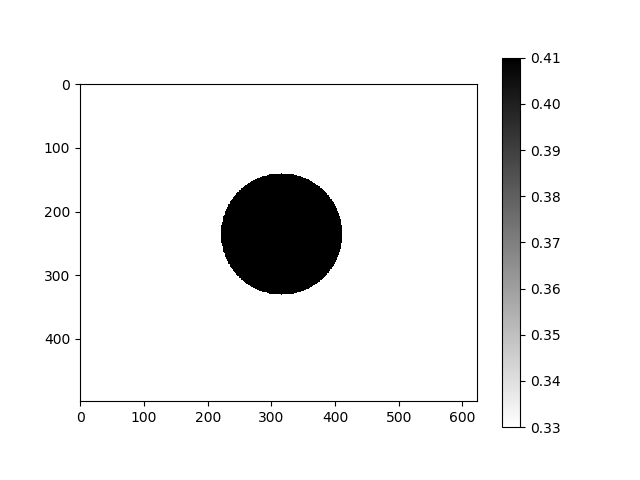

Supplement: Supplementary file 5 — Supplementary Information 5 A dataset of crystal violet staining experiment including raw images, software code and analysis results. [file 41598_2019_44167_MOESM5_ESM.zip › calibration/IMG_20190402_0005.jpg.1.png]

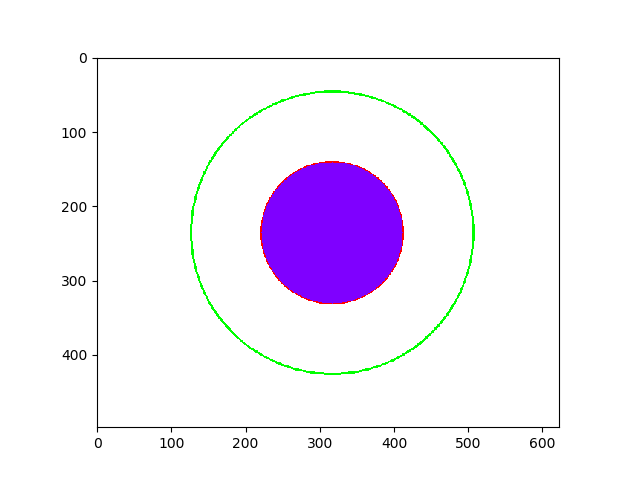

Supplement: Supplementary file 5 — Supplementary Information 5 A dataset of crystal violet staining experiment including raw images, software code and analysis results. [file 41598_2019_44167_MOESM5_ESM.zip › calibration/IMG_20190402_0005.jpg.2.png]

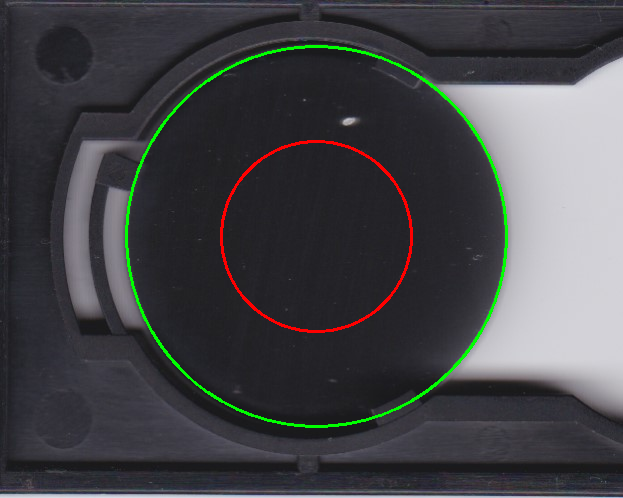

Supplement: Supplementary file 5 — Supplementary Information 5 A dataset of crystal violet staining experiment including raw images, software code and analysis results. [file 41598_2019_44167_MOESM5_ESM.zip › calibration/IMG_20190402_0005.jpg.tiff]

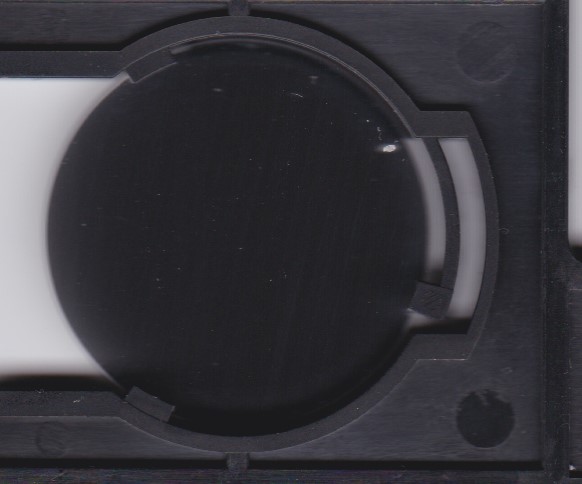

Supplement: Supplementary file 5 — Supplementary Information 5 A dataset of crystal violet staining experiment including raw images, software code and analysis results. [file 41598_2019_44167_MOESM5_ESM.zip › calibration/IMG_20190402_0006.jpg]

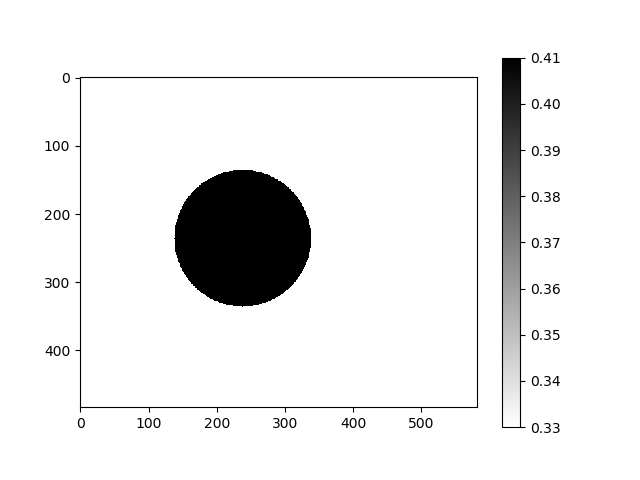

Supplement: Supplementary file 5 — Supplementary Information 5 A dataset of crystal violet staining experiment including raw images, software code and analysis results. [file 41598_2019_44167_MOESM5_ESM.zip › calibration/IMG_20190402_0006.jpg.1.png]

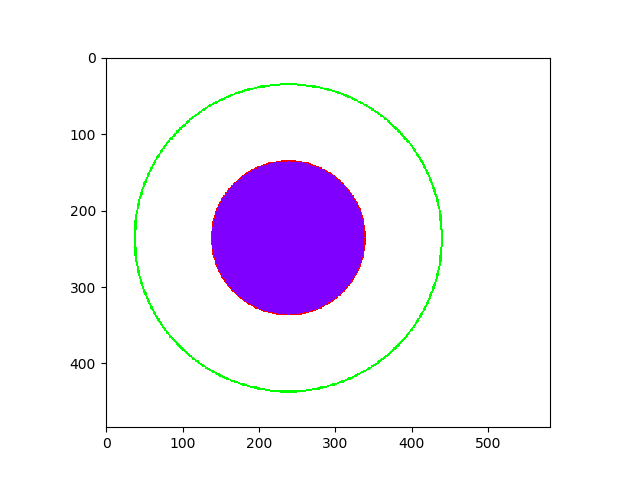

Supplement: Supplementary file 5 — Supplementary Information 5 A dataset of crystal violet staining experiment including raw images, software code and analysis results. [file 41598_2019_44167_MOESM5_ESM.zip › calibration/IMG_20190402_0006.jpg.2.png]

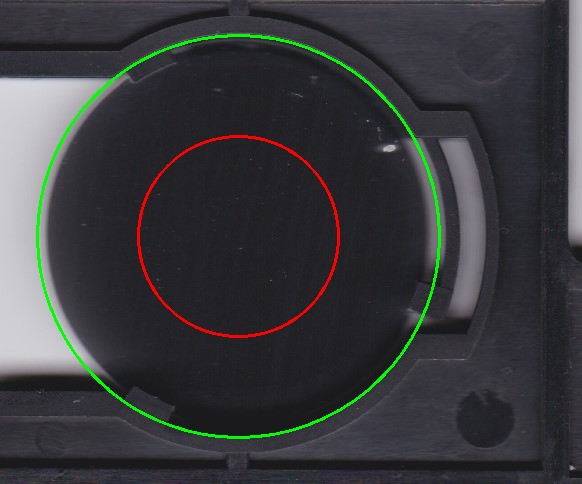

Supplement: Supplementary file 5 — Supplementary Information 5 A dataset of crystal violet staining experiment including raw images, software code and analysis results. [file 41598_2019_44167_MOESM5_ESM.zip › calibration/IMG_20190402_0006.jpg.tiff]

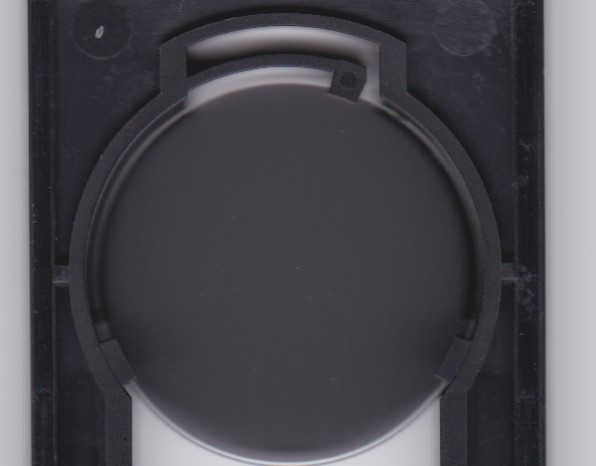

Supplement: Supplementary file 5 — Supplementary Information 5 A dataset of crystal violet staining experiment including raw images, software code and analysis results. [file 41598_2019_44167_MOESM5_ESM.zip › calibration/IMG_20190402_0007.jpg]

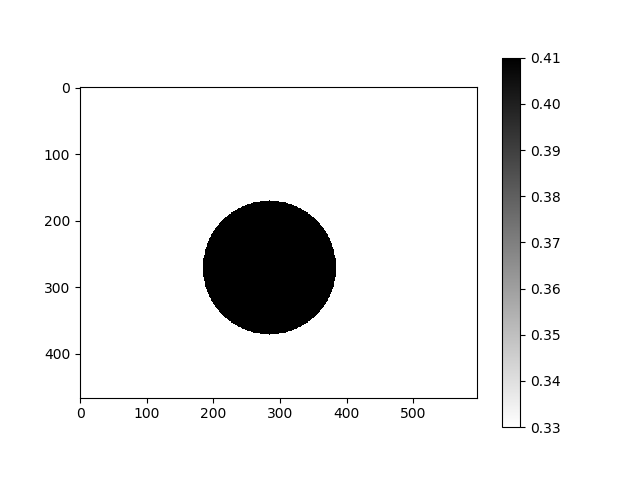

Supplement: Supplementary file 5 — Supplementary Information 5 A dataset of crystal violet staining experiment including raw images, software code and analysis results. [file 41598_2019_44167_MOESM5_ESM.zip › calibration/IMG_20190402_0007.jpg.1.png]

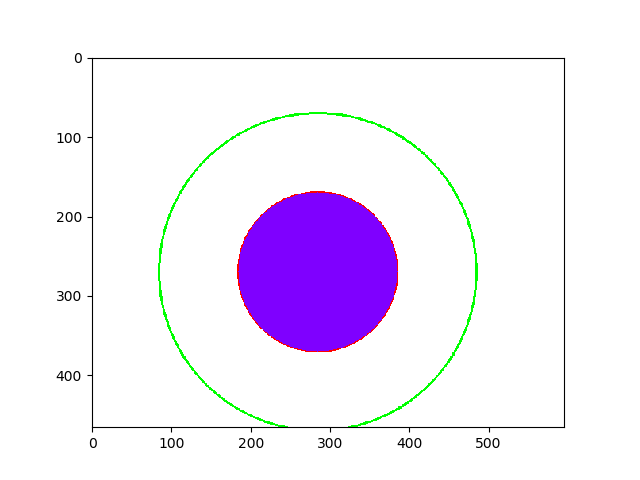

Supplement: Supplementary file 5 — Supplementary Information 5 A dataset of crystal violet staining experiment including raw images, software code and analysis results. [file 41598_2019_44167_MOESM5_ESM.zip › calibration/IMG_20190402_0007.jpg.2.png]

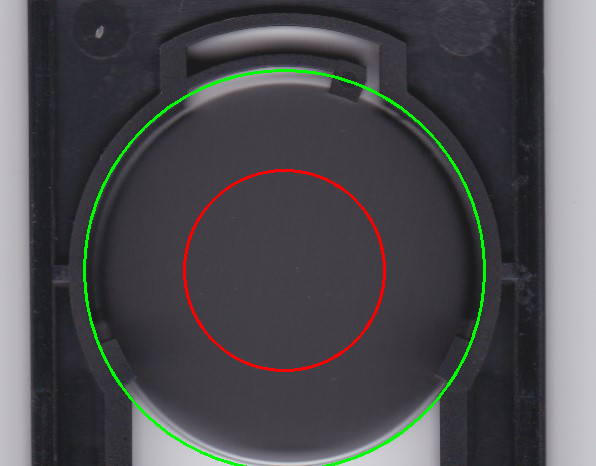

Supplement: Supplementary file 5 — Supplementary Information 5 A dataset of crystal violet staining experiment including raw images, software code and analysis results. [file 41598_2019_44167_MOESM5_ESM.zip › calibration/IMG_20190402_0007.jpg.tiff]

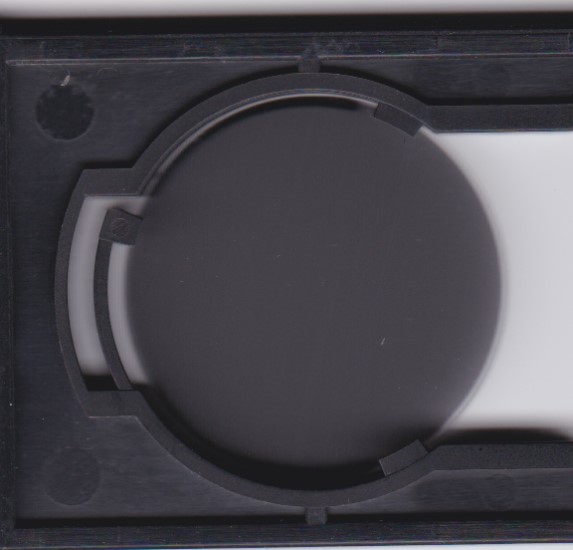

Supplement: Supplementary file 5 — Supplementary Information 5 A dataset of crystal violet staining experiment including raw images, software code and analysis results. [file 41598_2019_44167_MOESM5_ESM.zip › calibration/IMG_20190402_0008.jpg]

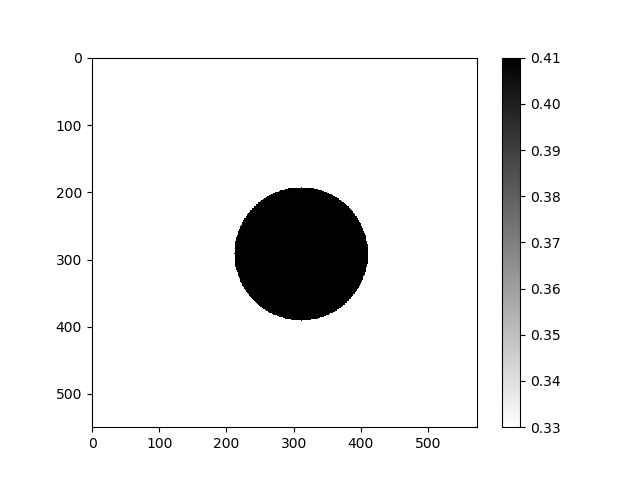

Supplement: Supplementary file 5 — Supplementary Information 5 A dataset of crystal violet staining experiment including raw images, software code and analysis results. [file 41598_2019_44167_MOESM5_ESM.zip › calibration/IMG_20190402_0008.jpg.1.png]

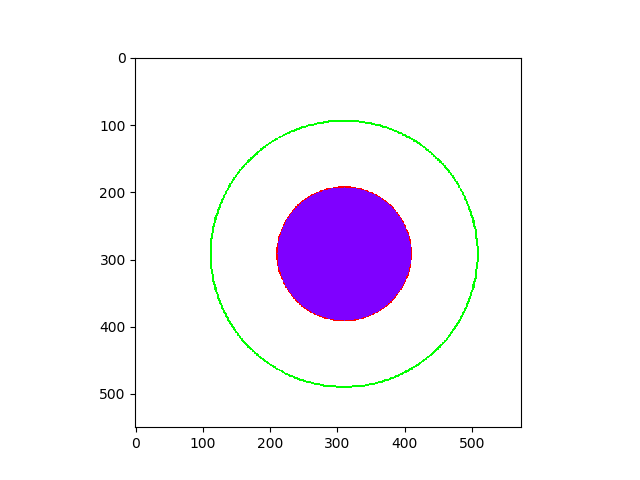

Supplement: Supplementary file 5 — Supplementary Information 5 A dataset of crystal violet staining experiment including raw images, software code and analysis results. [file 41598_2019_44167_MOESM5_ESM.zip › calibration/IMG_20190402_0008.jpg.2.png]

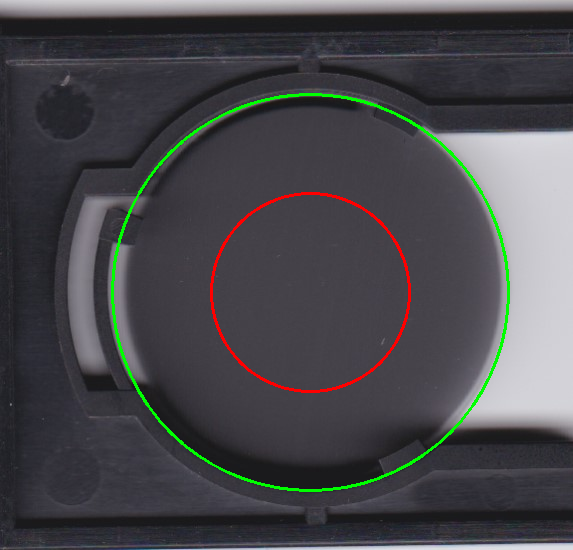

Supplement: Supplementary file 5 — Supplementary Information 5 A dataset of crystal violet staining experiment including raw images, software code and analysis results. [file 41598_2019_44167_MOESM5_ESM.zip › calibration/IMG_20190402_0008.jpg.tiff]

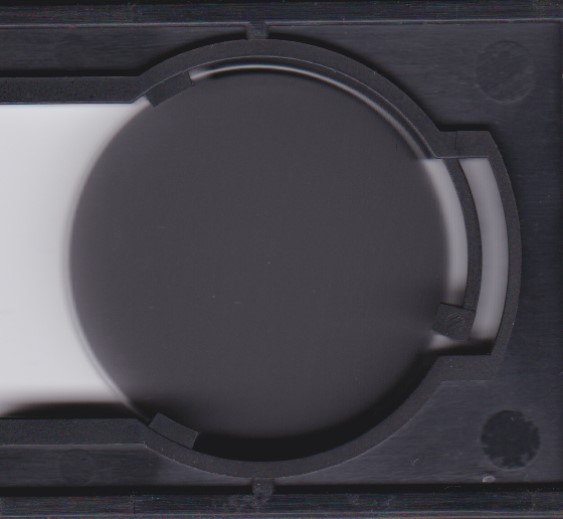

Supplement: Supplementary file 5 — Supplementary Information 5 A dataset of crystal violet staining experiment including raw images, software code and analysis results. [file 41598_2019_44167_MOESM5_ESM.zip › calibration/IMG_20190402_0009.jpg]

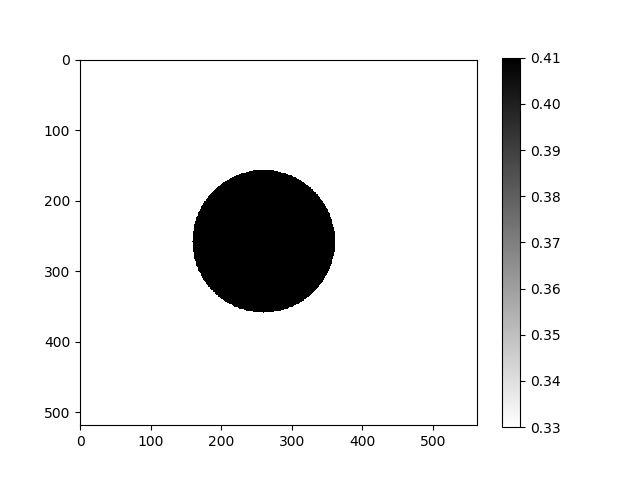

Supplement: Supplementary file 5 — Supplementary Information 5 A dataset of crystal violet staining experiment including raw images, software code and analysis results. [file 41598_2019_44167_MOESM5_ESM.zip › calibration/IMG_20190402_0009.jpg.1.png]

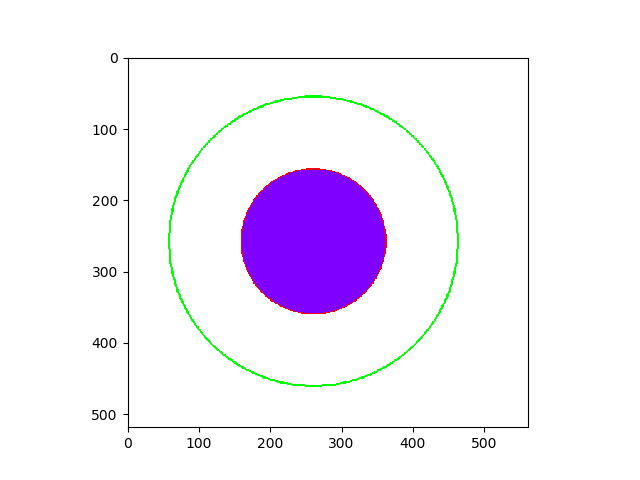

Supplement: Supplementary file 5 — Supplementary Information 5 A dataset of crystal violet staining experiment including raw images, software code and analysis results. [file 41598_2019_44167_MOESM5_ESM.zip › calibration/IMG_20190402_0009.jpg.2.png]

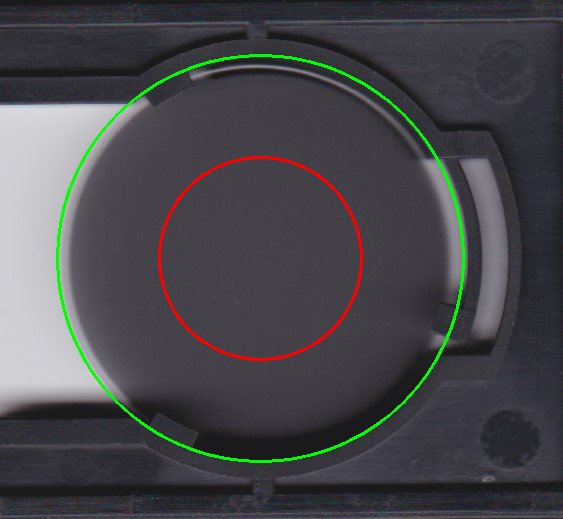

Supplement: Supplementary file 5 — Supplementary Information 5 A dataset of crystal violet staining experiment including raw images, software code and analysis results. [file 41598_2019_44167_MOESM5_ESM.zip › calibration/IMG_20190402_0009.jpg.tiff]

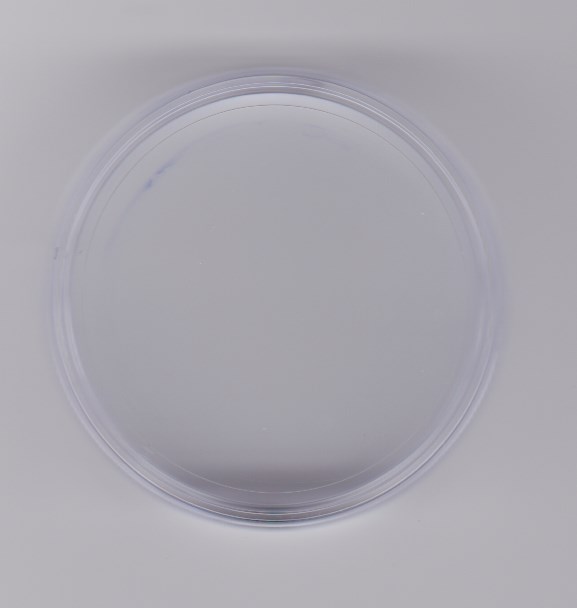

Supplement: Supplementary file 5 — Supplementary Information 5 A dataset of crystal violet staining experiment including raw images, software code and analysis results. [file 41598_2019_44167_MOESM5_ESM.zip › images/d00_p_1.jpg]

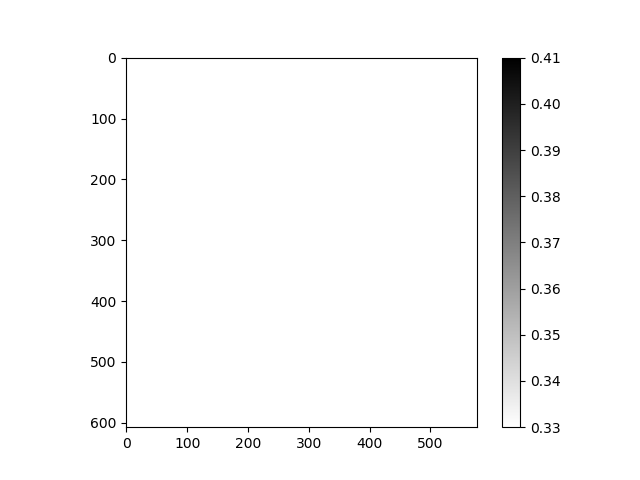

Supplement: Supplementary file 5 — Supplementary Information 5 A dataset of crystal violet staining experiment including raw images, software code and analysis results. [file 41598_2019_44167_MOESM5_ESM.zip › images/d00_p_1.jpg.1.png]

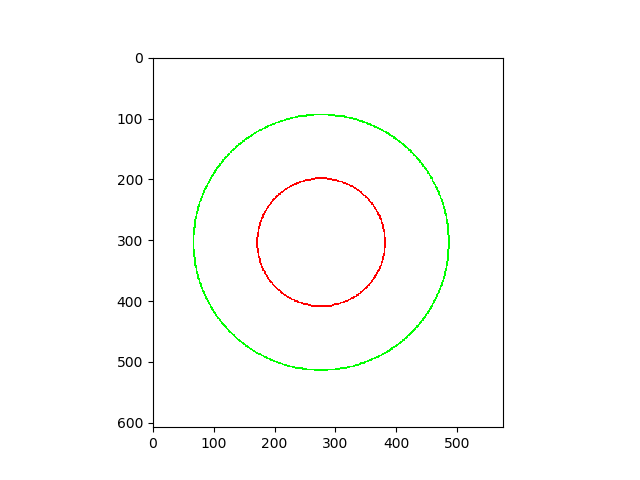

Supplement: Supplementary file 5 — Supplementary Information 5 A dataset of crystal violet staining experiment including raw images, software code and analysis results. [file 41598_2019_44167_MOESM5_ESM.zip › images/d00_p_1.jpg.2.png]

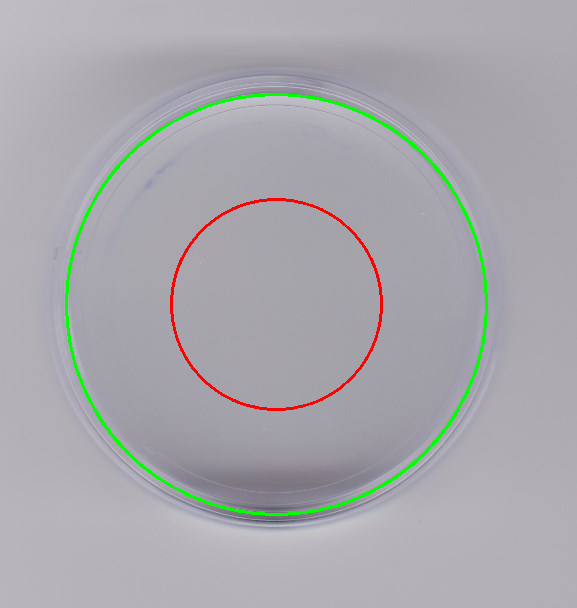

Supplement: Supplementary file 5 — Supplementary Information 5 A dataset of crystal violet staining experiment including raw images, software code and analysis results. [file 41598_2019_44167_MOESM5_ESM.zip › images/d00_p_1.jpg.tiff]

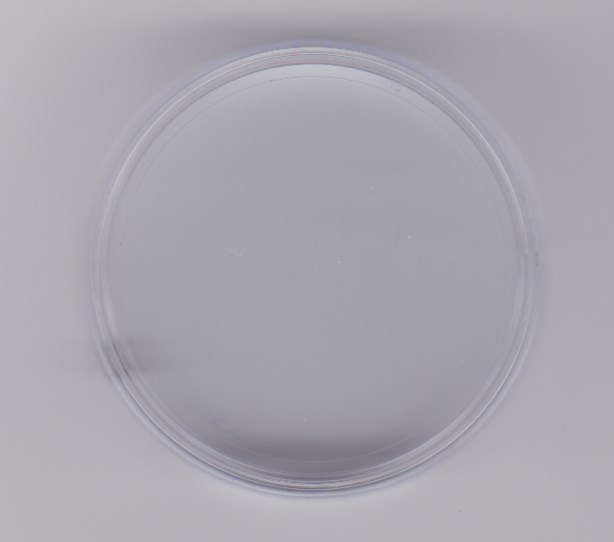

Supplement: Supplementary file 5 — Supplementary Information 5 A dataset of crystal violet staining experiment including raw images, software code and analysis results. [file 41598_2019_44167_MOESM5_ESM.zip › images/d00_p_2.jpg]

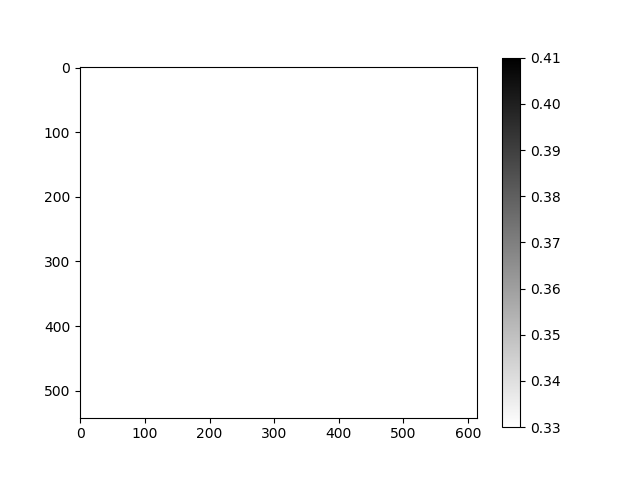

Supplement: Supplementary file 5 — Supplementary Information 5 A dataset of crystal violet staining experiment including raw images, software code and analysis results. [file 41598_2019_44167_MOESM5_ESM.zip › images/d00_p_2.jpg.1.png]

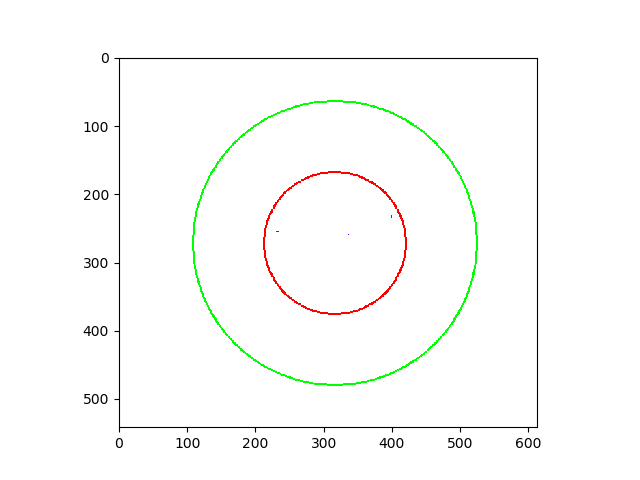

Supplement: Supplementary file 5 — Supplementary Information 5 A dataset of crystal violet staining experiment including raw images, software code and analysis results. [file 41598_2019_44167_MOESM5_ESM.zip › images/d00_p_2.jpg.2.png]

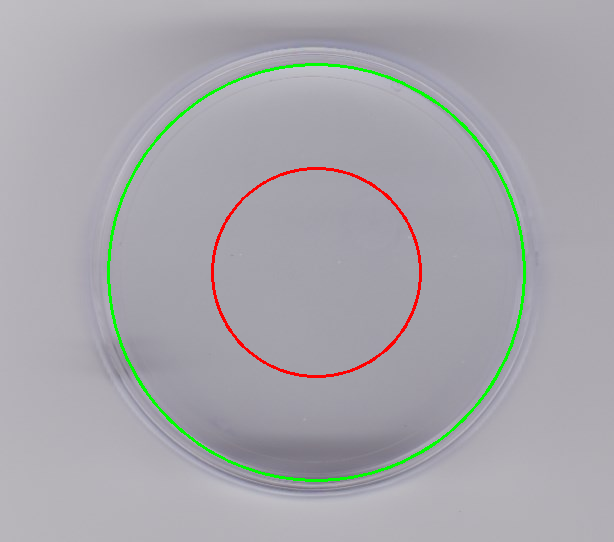

Supplement: Supplementary file 5 — Supplementary Information 5 A dataset of crystal violet staining experiment including raw images, software code and analysis results. [file 41598_2019_44167_MOESM5_ESM.zip › images/d00_p_2.jpg.tiff]

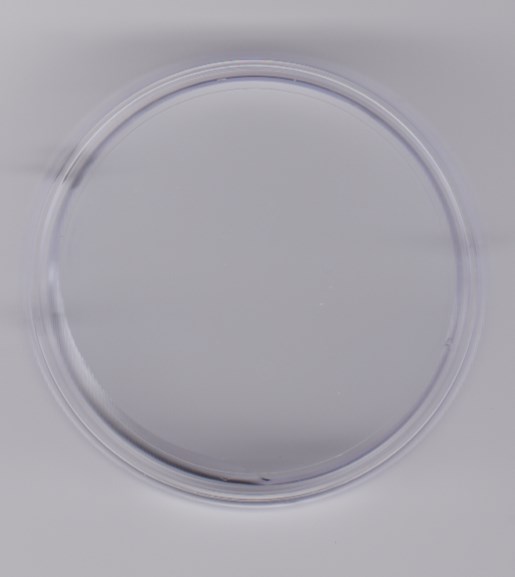

Supplement: Supplementary file 5 — Supplementary Information 5 A dataset of crystal violet staining experiment including raw images, software code and analysis results. [file 41598_2019_44167_MOESM5_ESM.zip › images/d00_p_3.jpg]

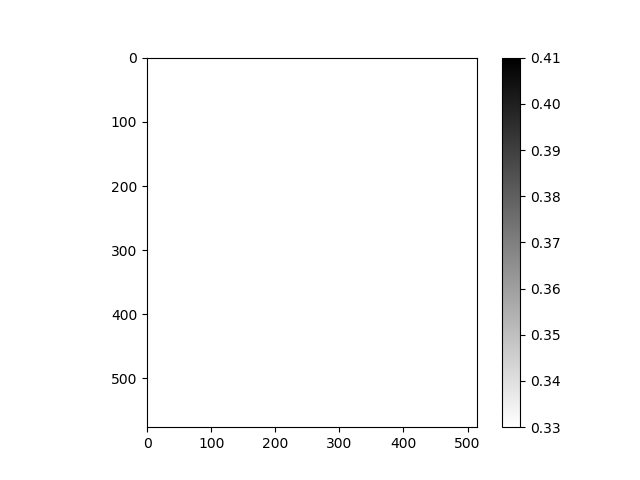

Supplement: Supplementary file 5 — Supplementary Information 5 A dataset of crystal violet staining experiment including raw images, software code and analysis results. [file 41598_2019_44167_MOESM5_ESM.zip › images/d00_p_3.jpg.1.png]

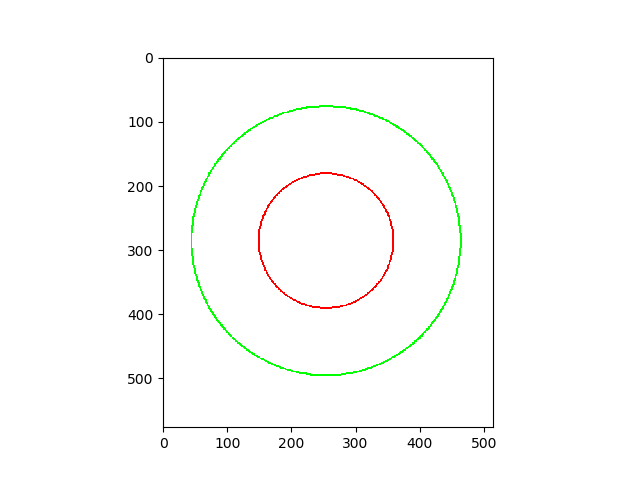

Supplement: Supplementary file 5 — Supplementary Information 5 A dataset of crystal violet staining experiment including raw images, software code and analysis results. [file 41598_2019_44167_MOESM5_ESM.zip › images/d00_p_3.jpg.2.png]

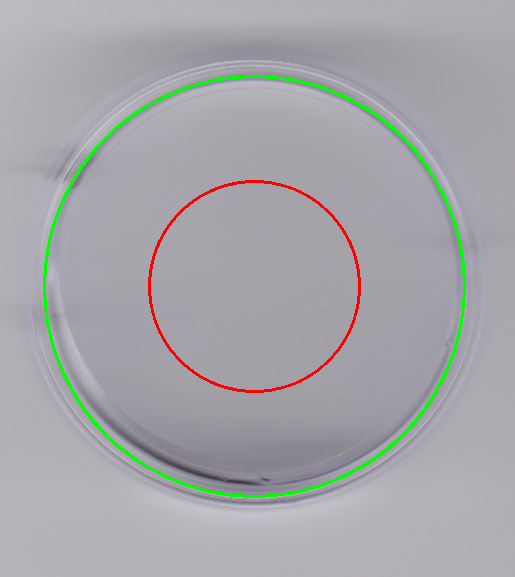

Supplement: Supplementary file 5 — Supplementary Information 5 A dataset of crystal violet staining experiment including raw images, software code and analysis results. [file 41598_2019_44167_MOESM5_ESM.zip › images/d00_p_3.jpg.tiff]

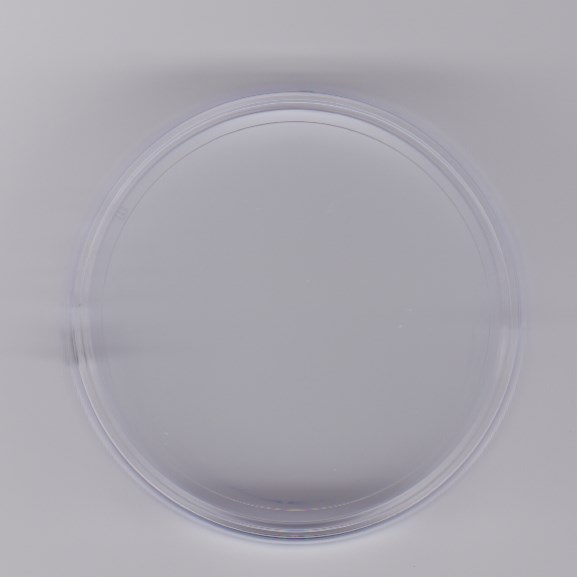

Supplement: Supplementary file 5 — Supplementary Information 5 A dataset of crystal violet staining experiment including raw images, software code and analysis results. [file 41598_2019_44167_MOESM5_ESM.zip › images/d00_p_4.jpg]

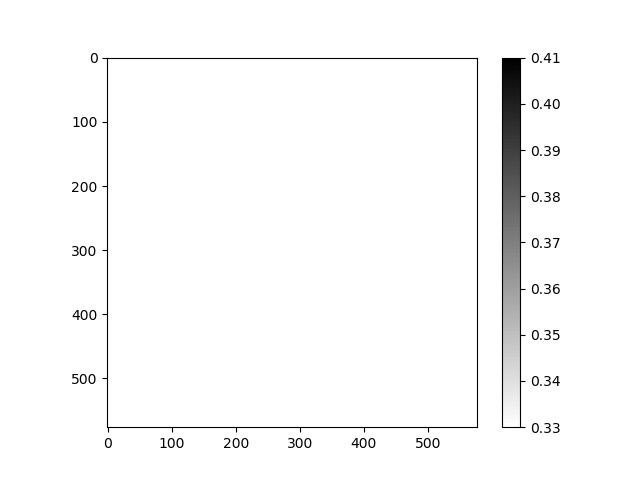

Supplement: Supplementary file 5 — Supplementary Information 5 A dataset of crystal violet staining experiment including raw images, software code and analysis results. [file 41598_2019_44167_MOESM5_ESM.zip › images/d00_p_4.jpg.1.png]

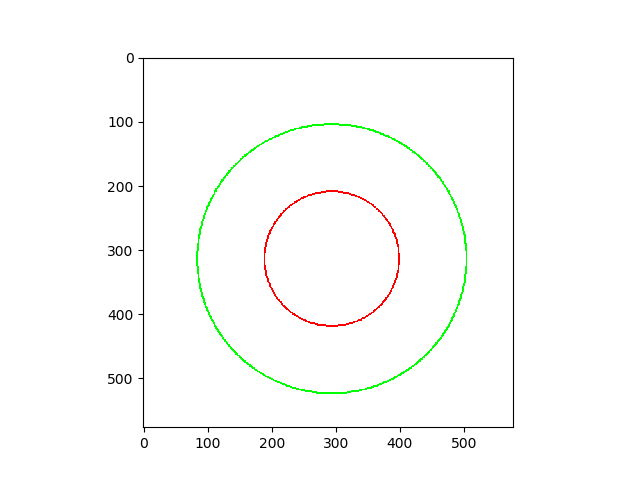

Supplement: Supplementary file 5 — Supplementary Information 5 A dataset of crystal violet staining experiment including raw images, software code and analysis results. [file 41598_2019_44167_MOESM5_ESM.zip › images/d00_p_4.jpg.2.png]

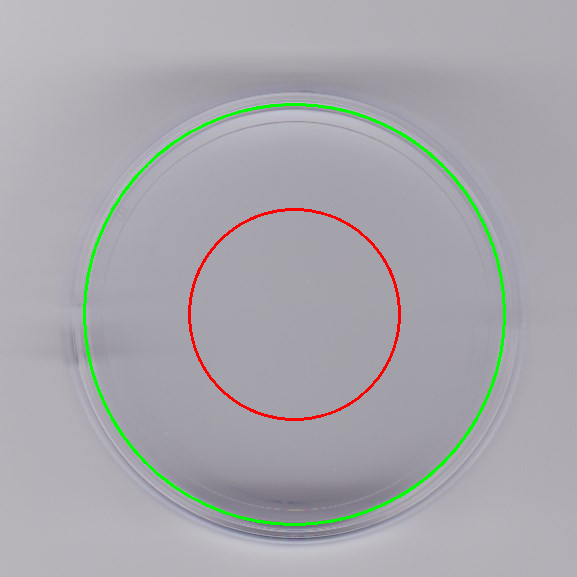

Supplement: Supplementary file 5 — Supplementary Information 5 A dataset of crystal violet staining experiment including raw images, software code and analysis results. [file 41598_2019_44167_MOESM5_ESM.zip › images/d00_p_4.jpg.tiff]

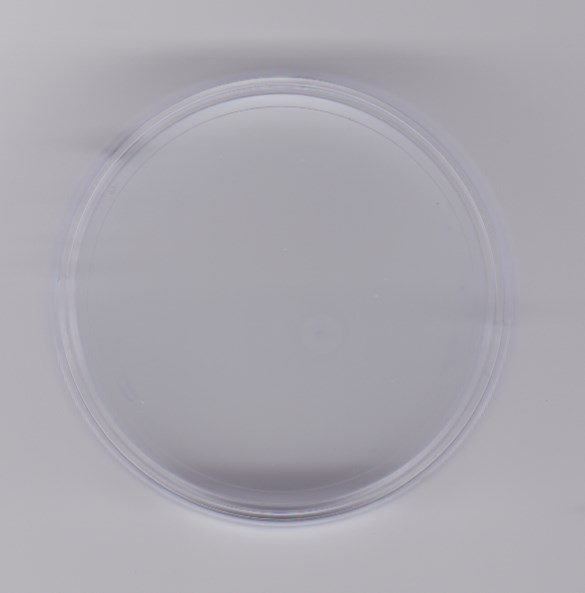

Supplement: Supplementary file 5 — Supplementary Information 5 A dataset of crystal violet staining experiment including raw images, software code and analysis results. [file 41598_2019_44167_MOESM5_ESM.zip › images/d00_p_5.jpg]

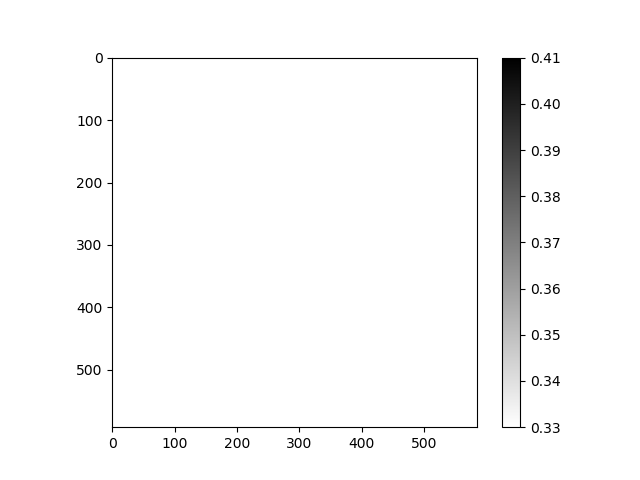

Supplement: Supplementary file 5 — Supplementary Information 5 A dataset of crystal violet staining experiment including raw images, software code and analysis results. [file 41598_2019_44167_MOESM5_ESM.zip › images/d00_p_5.jpg.1.png]

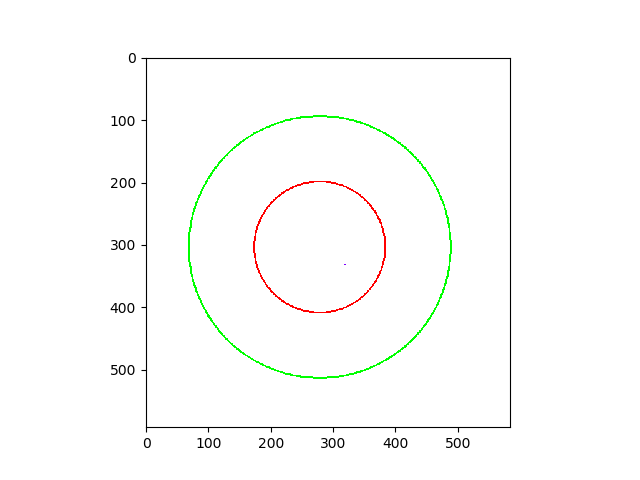

Supplement: Supplementary file 5 — Supplementary Information 5 A dataset of crystal violet staining experiment including raw images, software code and analysis results. [file 41598_2019_44167_MOESM5_ESM.zip › images/d00_p_5.jpg.2.png]

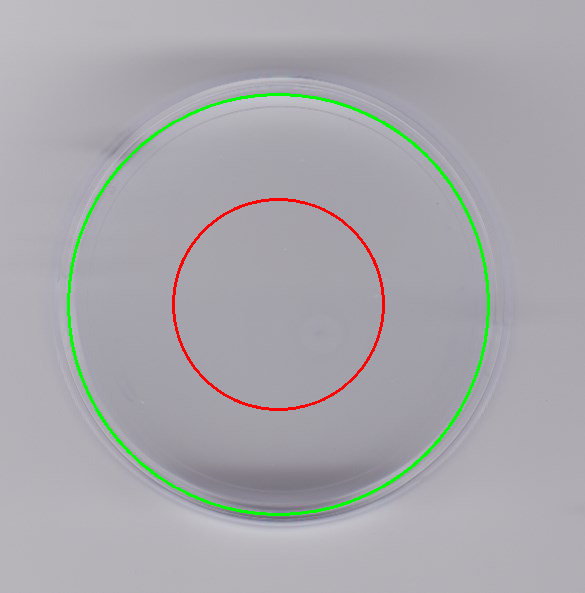

Supplement: Supplementary file 5 — Supplementary Information 5 A dataset of crystal violet staining experiment including raw images, software code and analysis results. [file 41598_2019_44167_MOESM5_ESM.zip › images/d00_p_5.jpg.tiff]

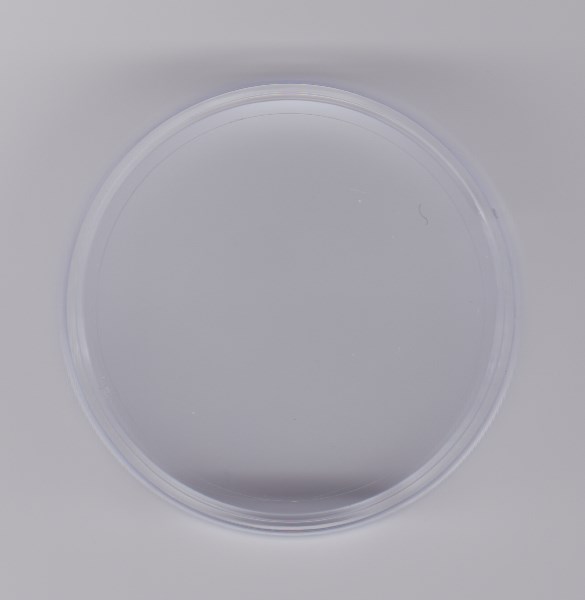

Supplement: Supplementary file 5 — Supplementary Information 5 A dataset of crystal violet staining experiment including raw images, software code and analysis results. [file 41598_2019_44167_MOESM5_ESM.zip › images/d00_v_1.jpg]

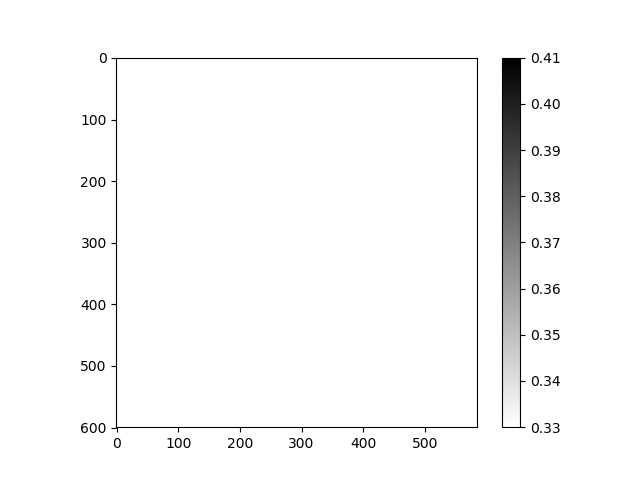

Supplement: Supplementary file 5 — Supplementary Information 5 A dataset of crystal violet staining experiment including raw images, software code and analysis results. [file 41598_2019_44167_MOESM5_ESM.zip › images/d00_v_1.jpg.1.png]

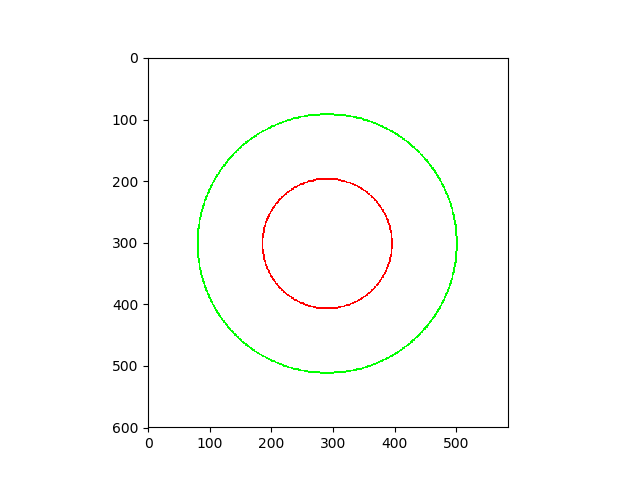

Supplement: Supplementary file 5 — Supplementary Information 5 A dataset of crystal violet staining experiment including raw images, software code and analysis results. [file 41598_2019_44167_MOESM5_ESM.zip › images/d00_v_1.jpg.2.png]

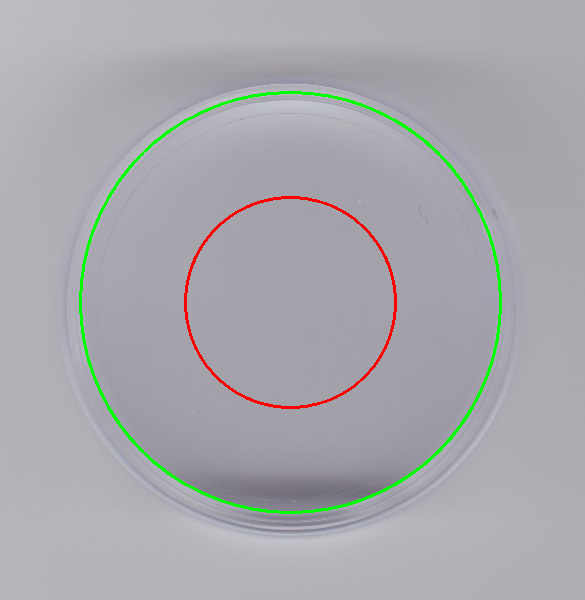

Supplement: Supplementary file 5 — Supplementary Information 5 A dataset of crystal violet staining experiment including raw images, software code and analysis results. [file 41598_2019_44167_MOESM5_ESM.zip › images/d00_v_1.jpg.tiff]

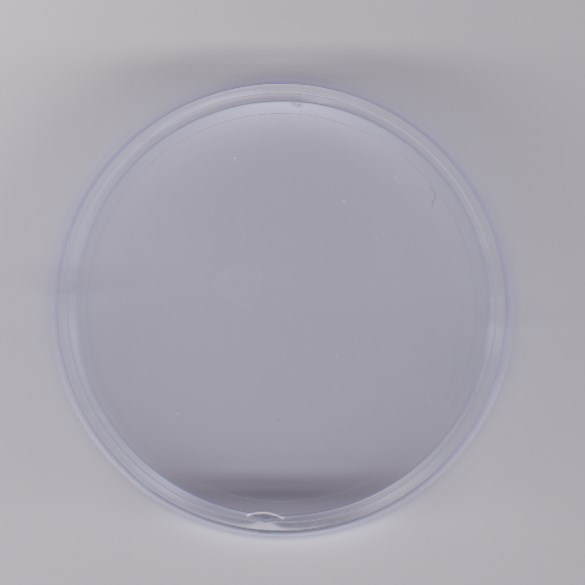

Supplement: Supplementary file 5 — Supplementary Information 5 A dataset of crystal violet staining experiment including raw images, software code and analysis results. [file 41598_2019_44167_MOESM5_ESM.zip › images/d00_v_2.jpg]

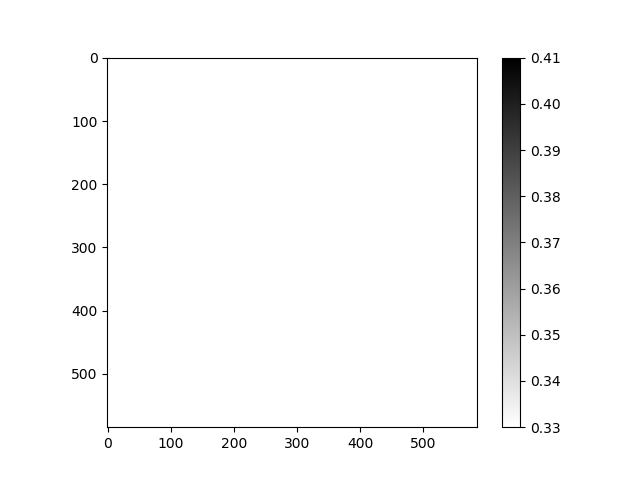

Supplement: Supplementary file 5 — Supplementary Information 5 A dataset of crystal violet staining experiment including raw images, software code and analysis results. [file 41598_2019_44167_MOESM5_ESM.zip › images/d00_v_2.jpg.1.png]

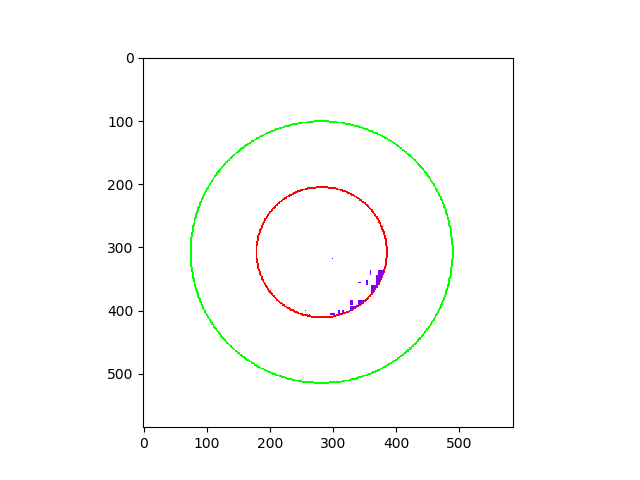

Supplement: Supplementary file 5 — Supplementary Information 5 A dataset of crystal violet staining experiment including raw images, software code and analysis results. [file 41598_2019_44167_MOESM5_ESM.zip › images/d00_v_2.jpg.2.png]

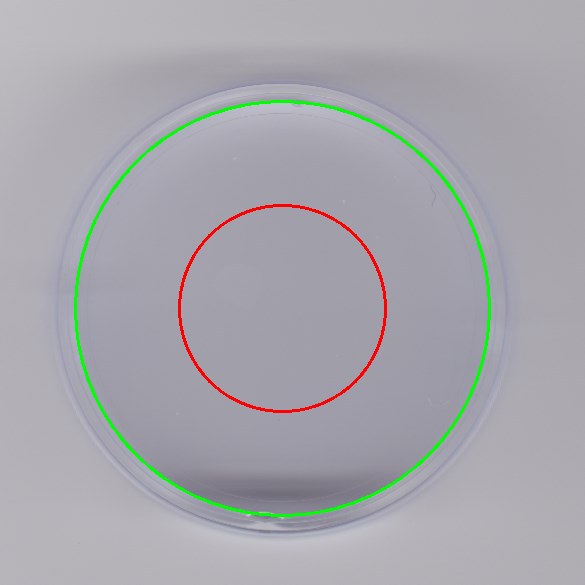

Supplement: Supplementary file 5 — Supplementary Information 5 A dataset of crystal violet staining experiment including raw images, software code and analysis results. [file 41598_2019_44167_MOESM5_ESM.zip › images/d00_v_2.jpg.tiff]

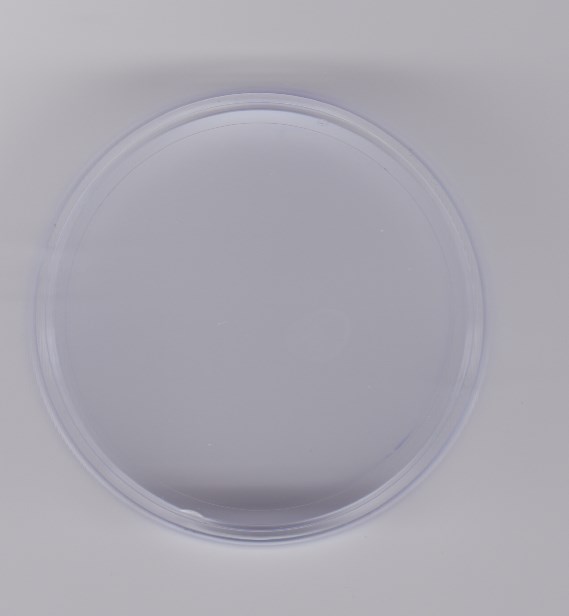

Supplement: Supplementary file 5 — Supplementary Information 5 A dataset of crystal violet staining experiment including raw images, software code and analysis results. [file 41598_2019_44167_MOESM5_ESM.zip › images/d00_v_3.jpg]

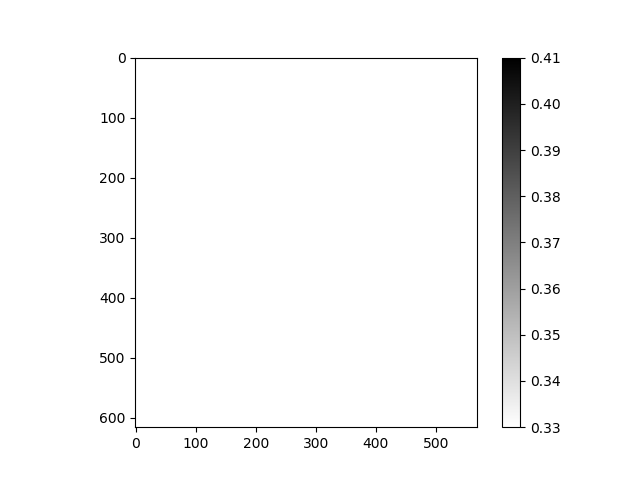

Supplement: Supplementary file 5 — Supplementary Information 5 A dataset of crystal violet staining experiment including raw images, software code and analysis results. [file 41598_2019_44167_MOESM5_ESM.zip › images/d00_v_3.jpg.1.png]

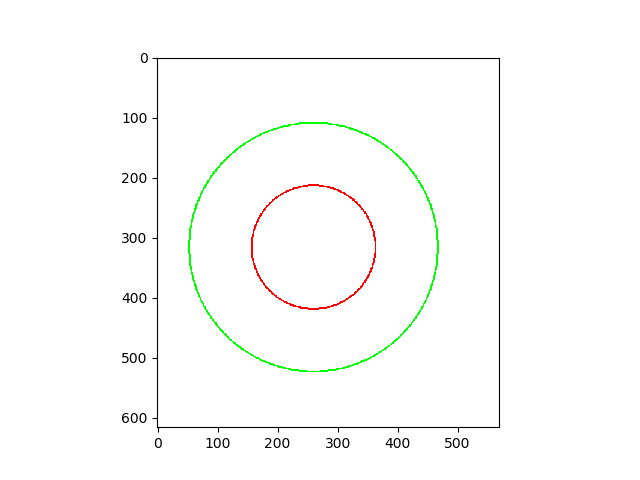

Supplement: Supplementary file 5 — Supplementary Information 5 A dataset of crystal violet staining experiment including raw images, software code and analysis results. [file 41598_2019_44167_MOESM5_ESM.zip › images/d00_v_3.jpg.2.png]

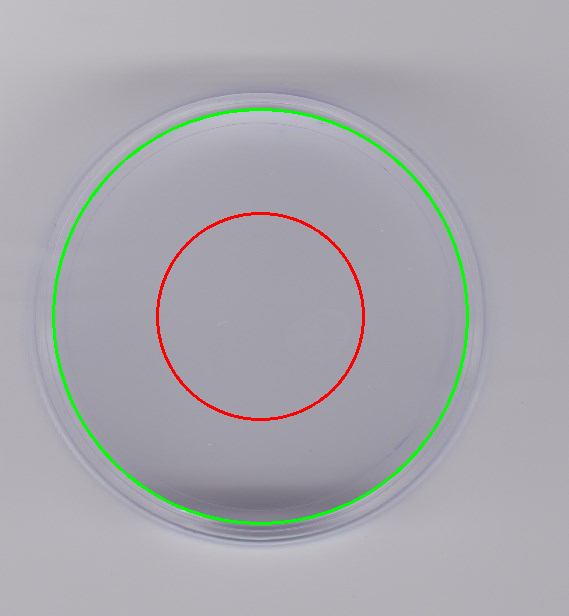

Supplement: Supplementary file 5 — Supplementary Information 5 A dataset of crystal violet staining experiment including raw images, software code and analysis results. [file 41598_2019_44167_MOESM5_ESM.zip › images/d00_v_3.jpg.tiff]

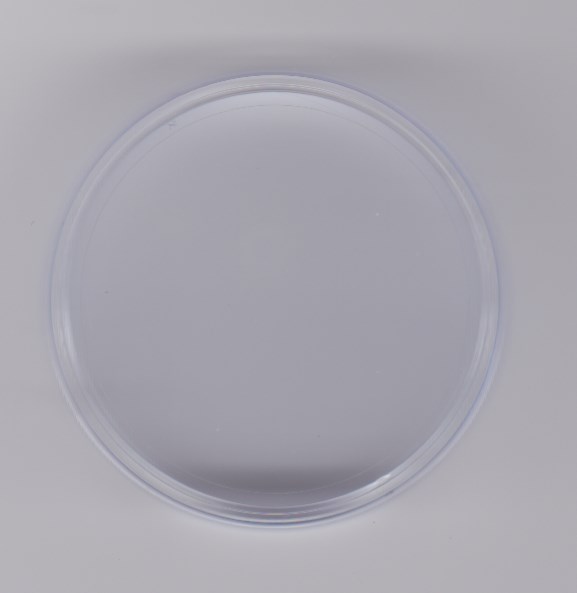

Supplement: Supplementary file 5 — Supplementary Information 5 A dataset of crystal violet staining experiment including raw images, software code and analysis results. [file 41598_2019_44167_MOESM5_ESM.zip › images/d00_v_4.jpg]

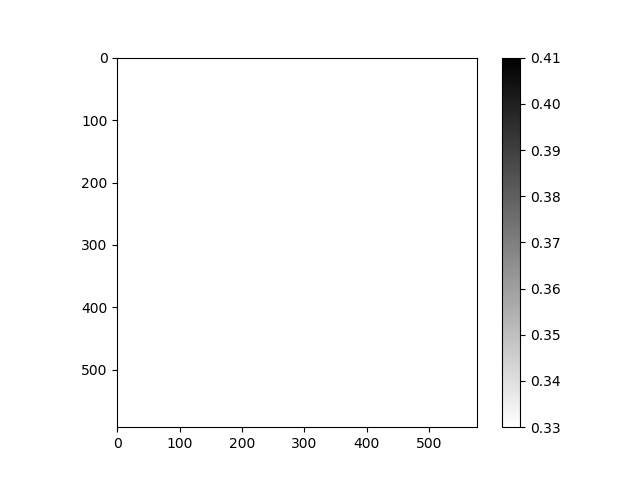

Supplement: Supplementary file 5 — Supplementary Information 5 A dataset of crystal violet staining experiment including raw images, software code and analysis results. [file 41598_2019_44167_MOESM5_ESM.zip › images/d00_v_4.jpg.1.png]

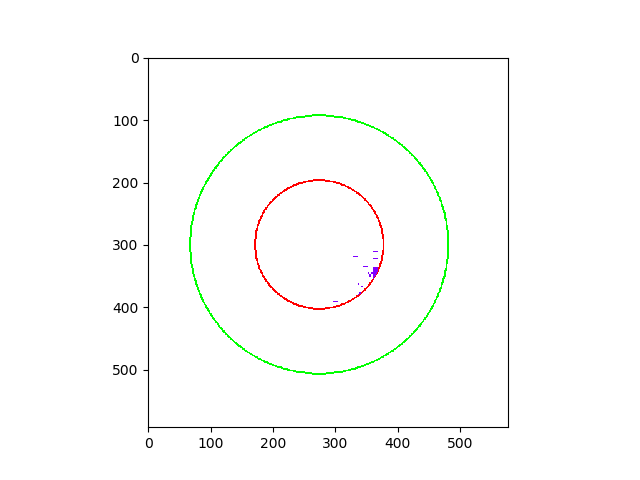

Supplement: Supplementary file 5 — Supplementary Information 5 A dataset of crystal violet staining experiment including raw images, software code and analysis results. [file 41598_2019_44167_MOESM5_ESM.zip › images/d00_v_4.jpg.2.png]

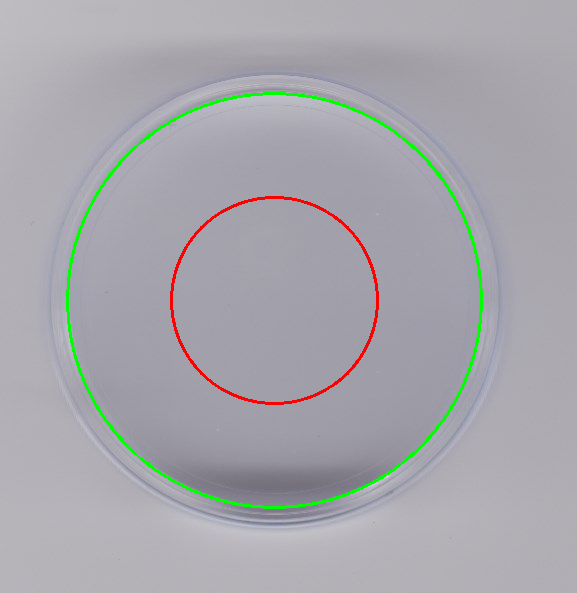

Supplement: Supplementary file 5 — Supplementary Information 5 A dataset of crystal violet staining experiment including raw images, software code and analysis results. [file 41598_2019_44167_MOESM5_ESM.zip › images/d00_v_4.jpg.tiff]

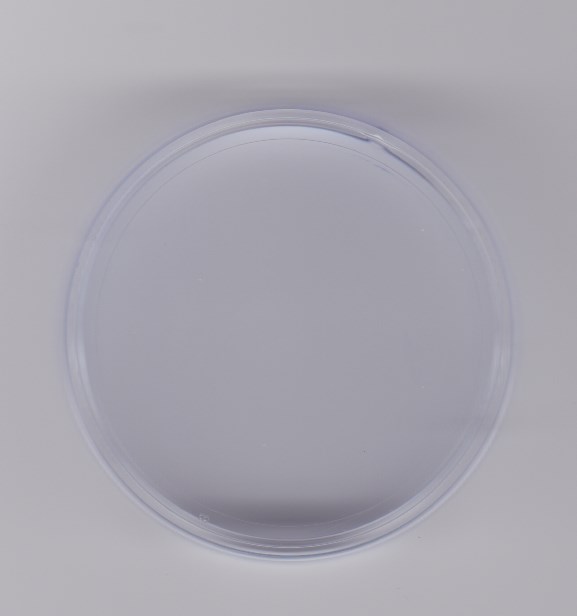

Supplement: Supplementary file 5 — Supplementary Information 5 A dataset of crystal violet staining experiment including raw images, software code and analysis results. [file 41598_2019_44167_MOESM5_ESM.zip › images/d00_v_5.jpg]

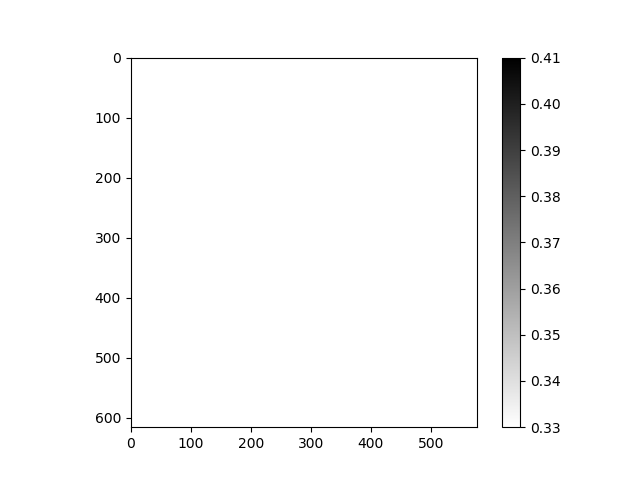

Supplement: Supplementary file 5 — Supplementary Information 5 A dataset of crystal violet staining experiment including raw images, software code and analysis results. [file 41598_2019_44167_MOESM5_ESM.zip › images/d00_v_5.jpg.1.png]

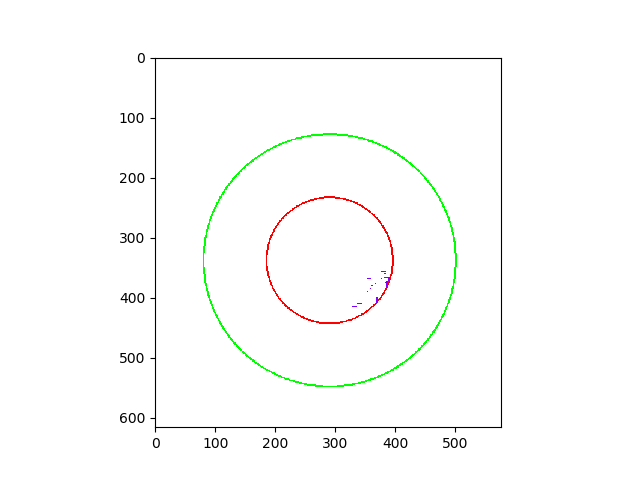

Supplement: Supplementary file 5 — Supplementary Information 5 A dataset of crystal violet staining experiment including raw images, software code and analysis results. [file 41598_2019_44167_MOESM5_ESM.zip › images/d00_v_5.jpg.2.png]

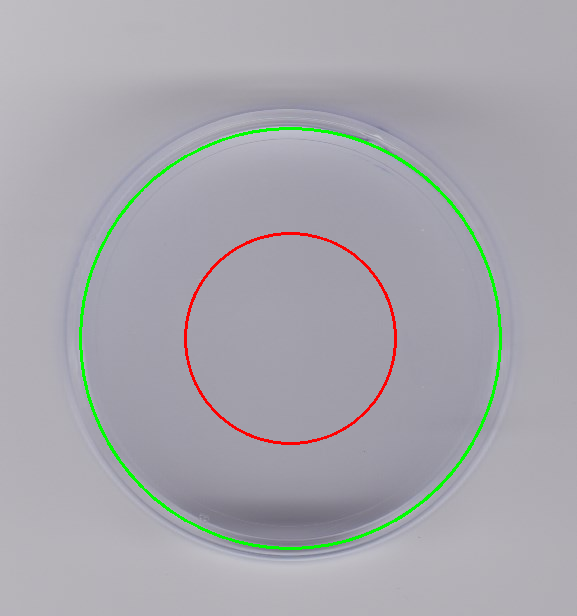

Supplement: Supplementary file 5 — Supplementary Information 5 A dataset of crystal violet staining experiment including raw images, software code and analysis results. [file 41598_2019_44167_MOESM5_ESM.zip › images/d00_v_5.jpg.tiff]

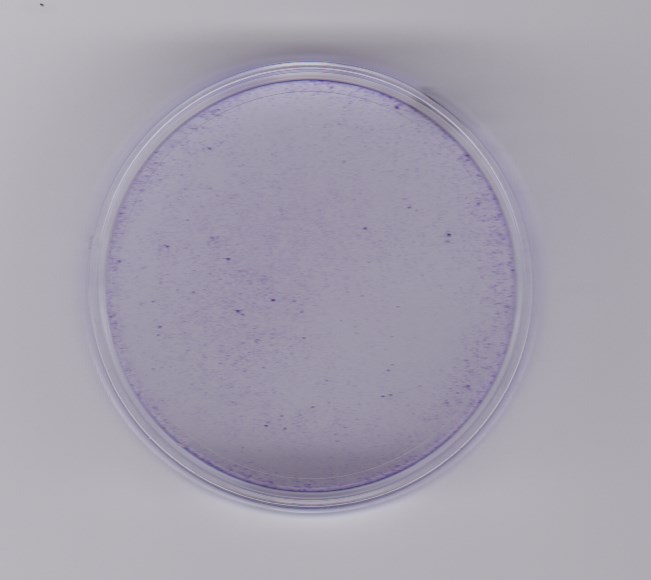

Supplement: Supplementary file 5 — Supplementary Information 5 A dataset of crystal violet staining experiment including raw images, software code and analysis results. [file 41598_2019_44167_MOESM5_ESM.zip › images/d07_p_1.jpg]

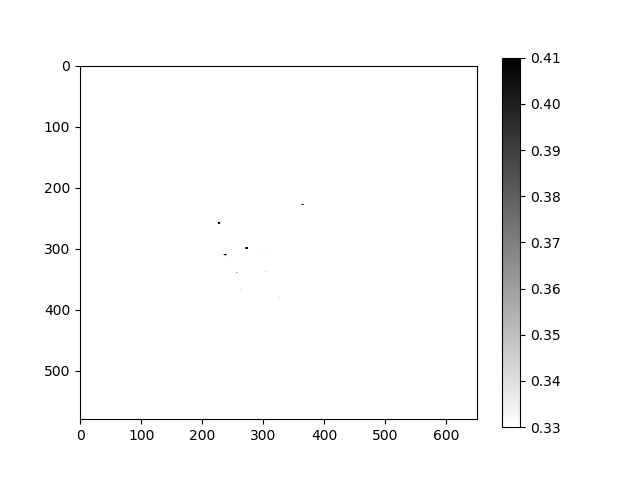

Supplement: Supplementary file 5 — Supplementary Information 5 A dataset of crystal violet staining experiment including raw images, software code and analysis results. [file 41598_2019_44167_MOESM5_ESM.zip › images/d07_p_1.jpg.1.png]

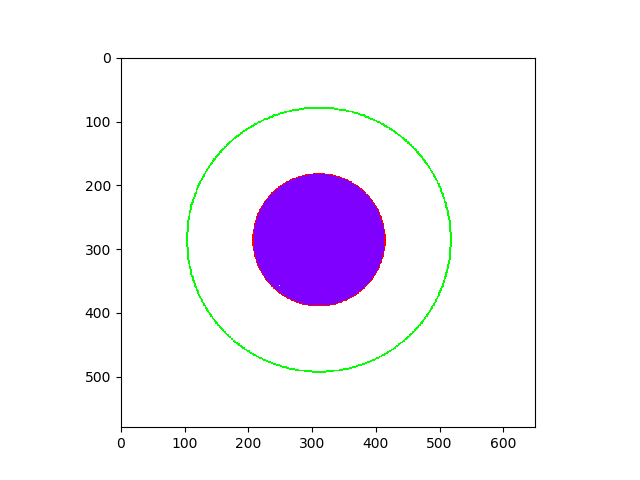

Supplement: Supplementary file 5 — Supplementary Information 5 A dataset of crystal violet staining experiment including raw images, software code and analysis results. [file 41598_2019_44167_MOESM5_ESM.zip › images/d07_p_1.jpg.2.png]

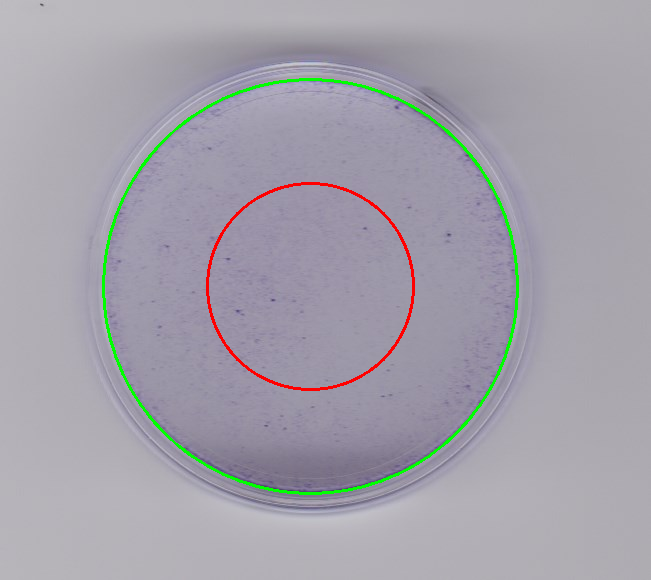

Supplement: Supplementary file 5 — Supplementary Information 5 A dataset of crystal violet staining experiment including raw images, software code and analysis results. [file 41598_2019_44167_MOESM5_ESM.zip › images/d07_p_1.jpg.tiff]

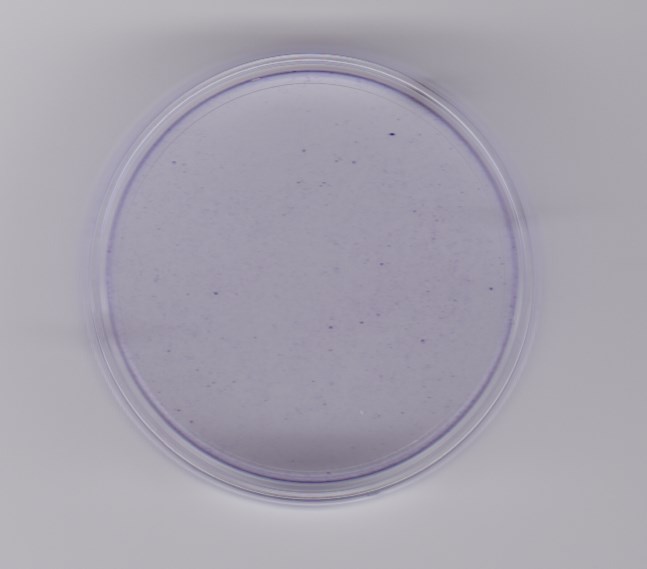

Supplement: Supplementary file 5 — Supplementary Information 5 A dataset of crystal violet staining experiment including raw images, software code and analysis results. [file 41598_2019_44167_MOESM5_ESM.zip › images/d07_p_2.jpg]

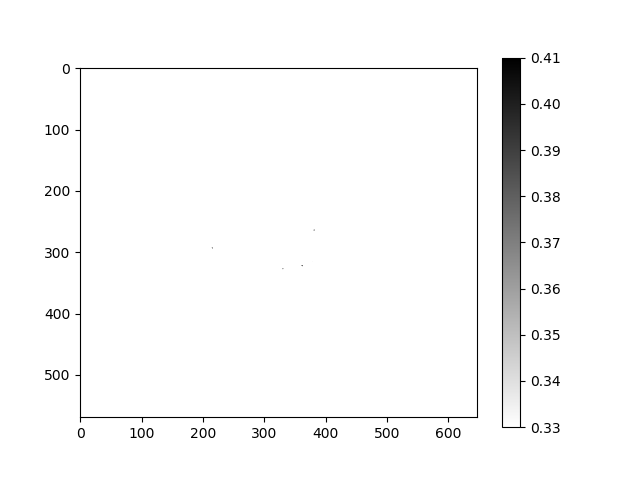

Supplement: Supplementary file 5 — Supplementary Information 5 A dataset of crystal violet staining experiment including raw images, software code and analysis results. [file 41598_2019_44167_MOESM5_ESM.zip › images/d07_p_2.jpg.1.png]

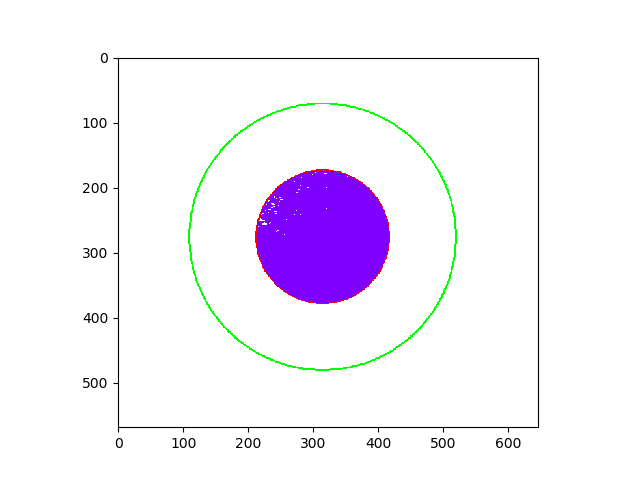

Supplement: Supplementary file 5 — Supplementary Information 5 A dataset of crystal violet staining experiment including raw images, software code and analysis results. [file 41598_2019_44167_MOESM5_ESM.zip › images/d07_p_2.jpg.2.png]

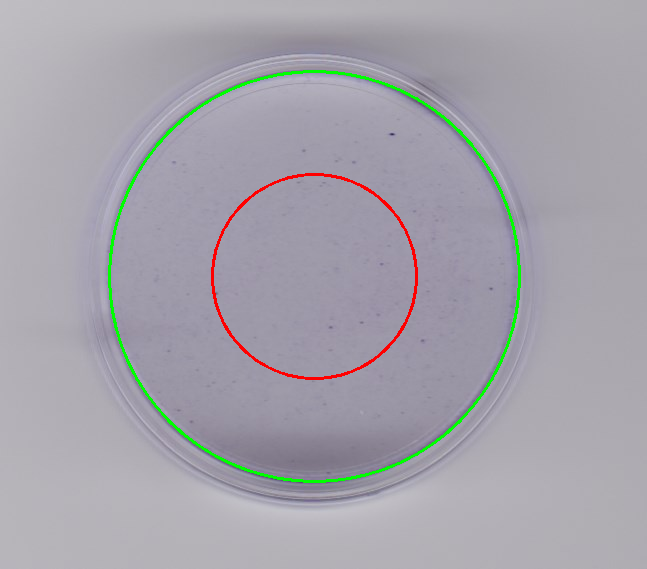

Supplement: Supplementary file 5 — Supplementary Information 5 A dataset of crystal violet staining experiment including raw images, software code and analysis results. [file 41598_2019_44167_MOESM5_ESM.zip › images/d07_p_2.jpg.tiff]

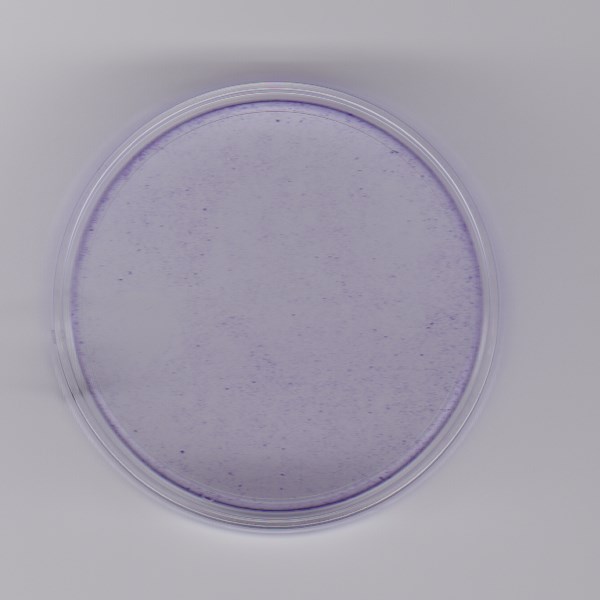

Supplement: Supplementary file 5 — Supplementary Information 5 A dataset of crystal violet staining experiment including raw images, software code and analysis results. [file 41598_2019_44167_MOESM5_ESM.zip › images/d07_p_3.jpg]

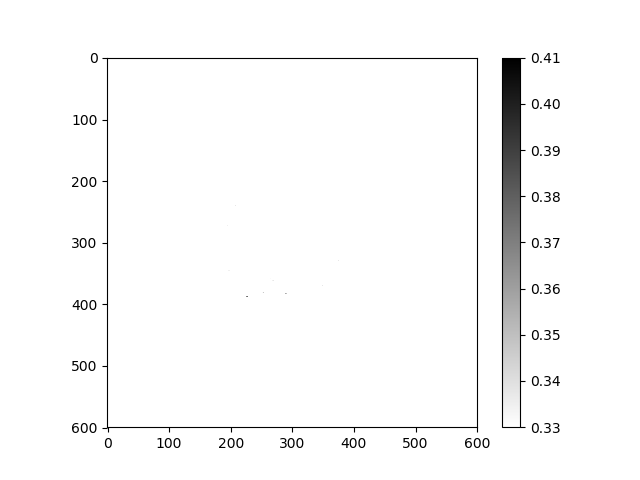

Supplement: Supplementary file 5 — Supplementary Information 5 A dataset of crystal violet staining experiment including raw images, software code and analysis results. [file 41598_2019_44167_MOESM5_ESM.zip › images/d07_p_3.jpg.1.png]

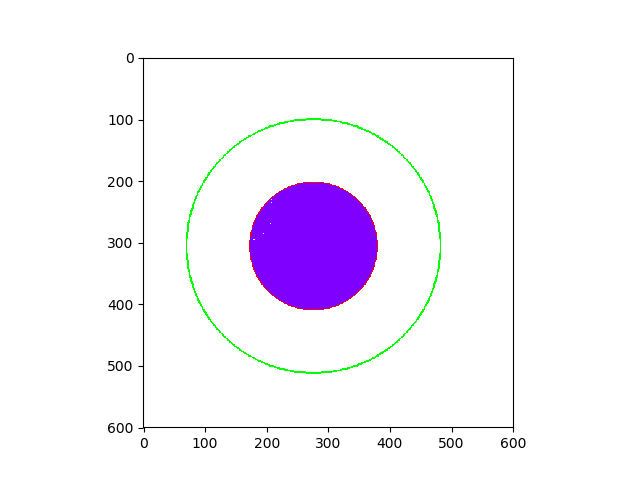

Supplement: Supplementary file 5 — Supplementary Information 5 A dataset of crystal violet staining experiment including raw images, software code and analysis results. [file 41598_2019_44167_MOESM5_ESM.zip › images/d07_p_3.jpg.2.png]

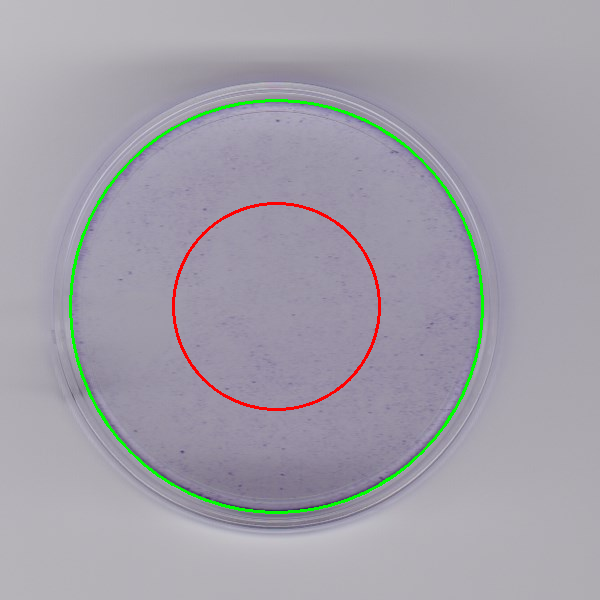

Supplement: Supplementary file 5 — Supplementary Information 5 A dataset of crystal violet staining experiment including raw images, software code and analysis results. [file 41598_2019_44167_MOESM5_ESM.zip › images/d07_p_3.jpg.tiff]

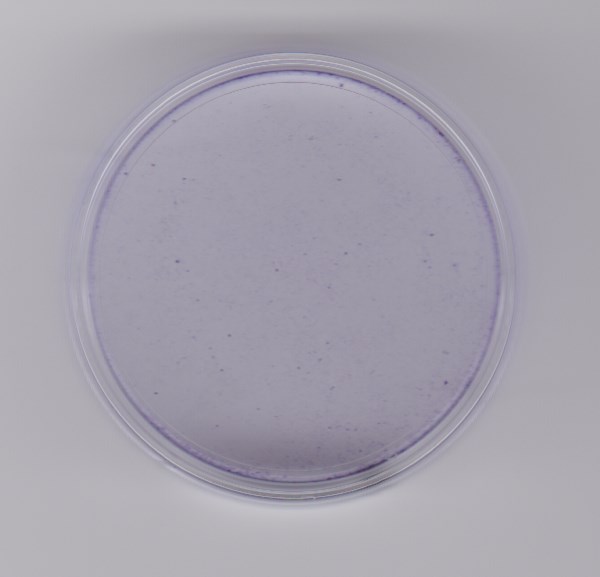

Supplement: Supplementary file 5 — Supplementary Information 5 A dataset of crystal violet staining experiment including raw images, software code and analysis results. [file 41598_2019_44167_MOESM5_ESM.zip › images/d07_p_4.jpg]

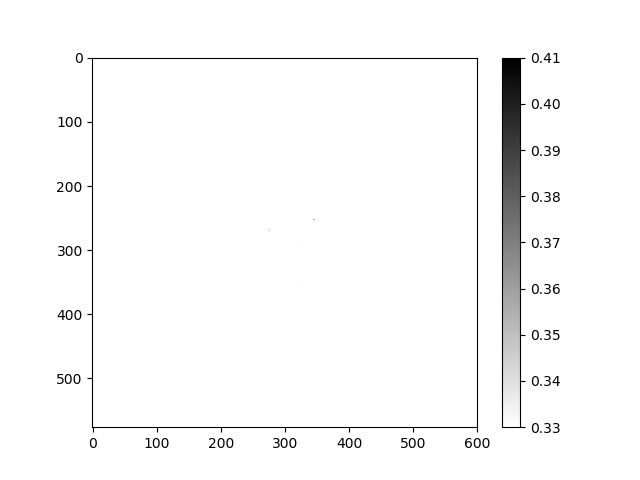

Supplement: Supplementary file 5 — Supplementary Information 5 A dataset of crystal violet staining experiment including raw images, software code and analysis results. [file 41598_2019_44167_MOESM5_ESM.zip › images/d07_p_4.jpg.1.png]

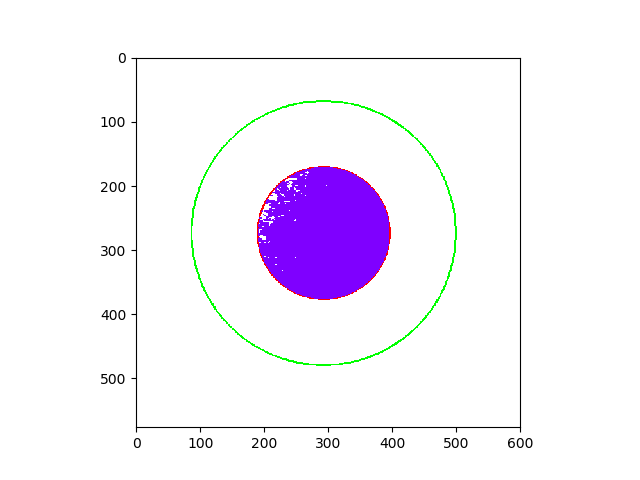

Supplement: Supplementary file 5 — Supplementary Information 5 A dataset of crystal violet staining experiment including raw images, software code and analysis results. [file 41598_2019_44167_MOESM5_ESM.zip › images/d07_p_4.jpg.2.png]

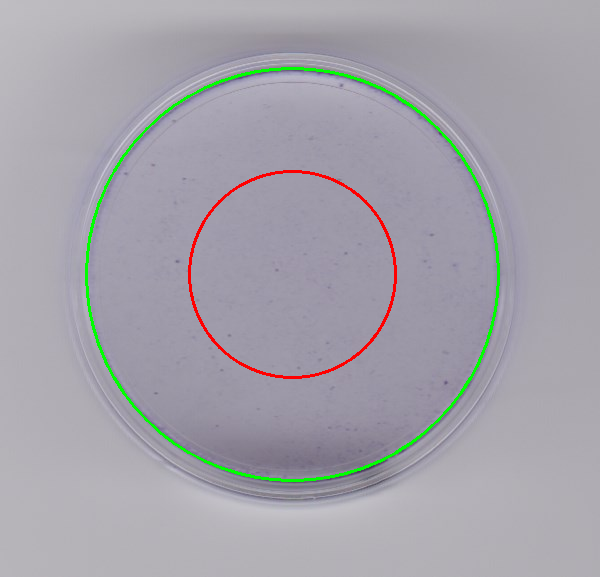

Supplement: Supplementary file 5 — Supplementary Information 5 A dataset of crystal violet staining experiment including raw images, software code and analysis results. [file 41598_2019_44167_MOESM5_ESM.zip › images/d07_p_4.jpg.tiff]

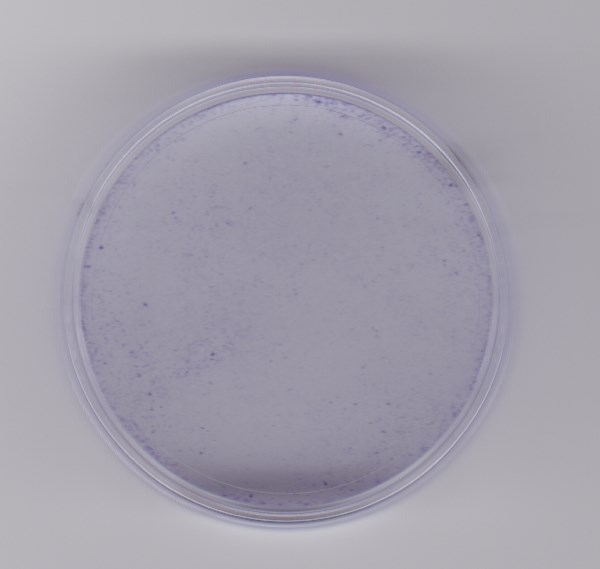

Supplement: Supplementary file 5 — Supplementary Information 5 A dataset of crystal violet staining experiment including raw images, software code and analysis results. [file 41598_2019_44167_MOESM5_ESM.zip › images/d07_p_5.jpg]

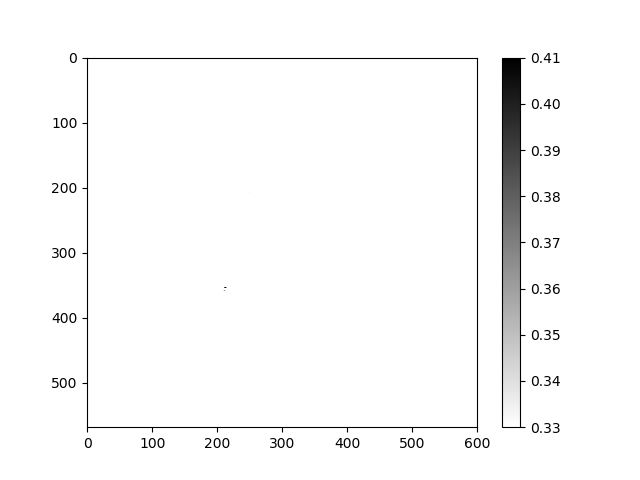

Supplement: Supplementary file 5 — Supplementary Information 5 A dataset of crystal violet staining experiment including raw images, software code and analysis results. [file 41598_2019_44167_MOESM5_ESM.zip › images/d07_p_5.jpg.1.png]

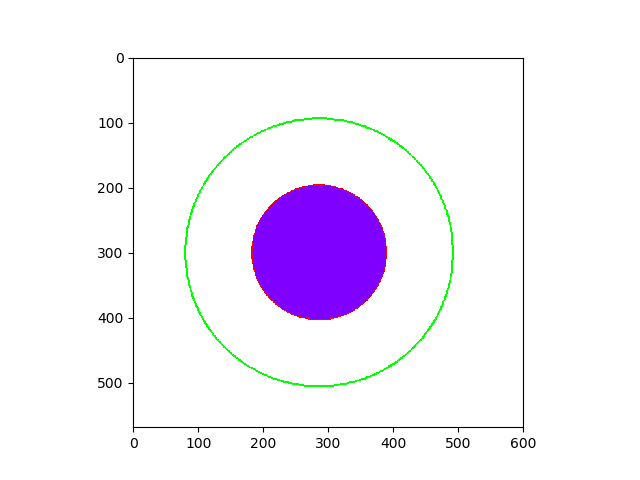

Supplement: Supplementary file 5 — Supplementary Information 5 A dataset of crystal violet staining experiment including raw images, software code and analysis results. [file 41598_2019_44167_MOESM5_ESM.zip › images/d07_p_5.jpg.2.png]

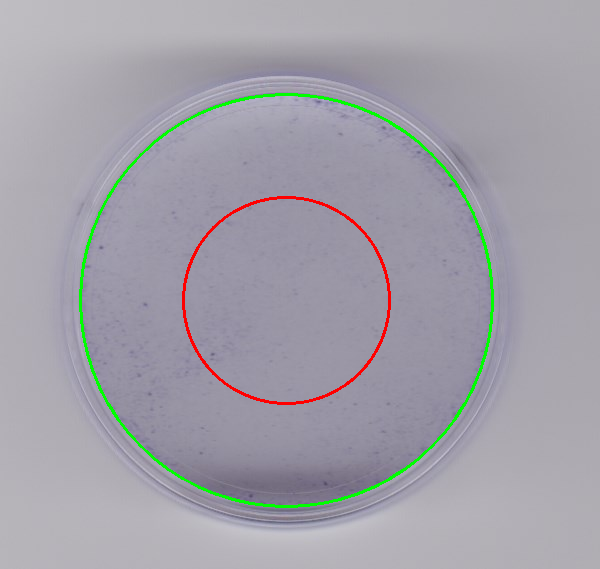

Supplement: Supplementary file 5 — Supplementary Information 5 A dataset of crystal violet staining experiment including raw images, software code and analysis results. [file 41598_2019_44167_MOESM5_ESM.zip › images/d07_p_5.jpg.tiff]

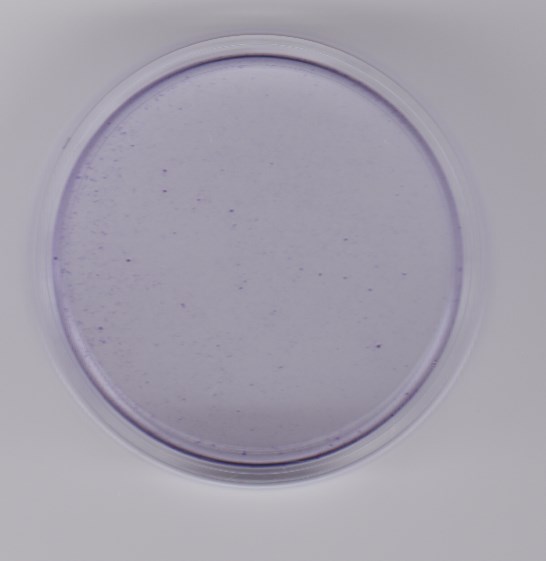

Supplement: Supplementary file 5 — Supplementary Information 5 A dataset of crystal violet staining experiment including raw images, software code and analysis results. [file 41598_2019_44167_MOESM5_ESM.zip › images/d07_v_1.jpg]

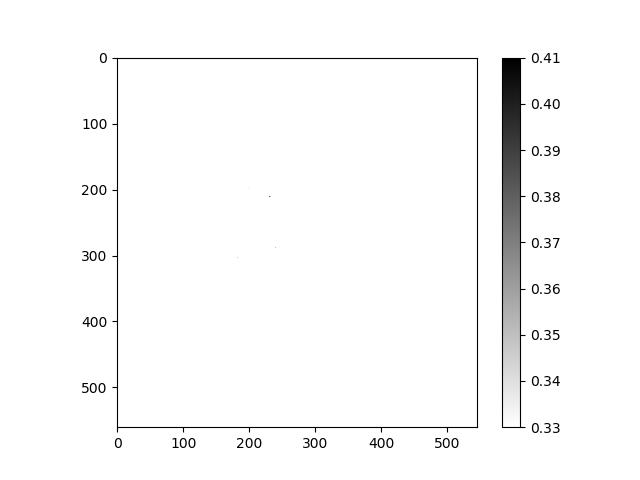

Supplement: Supplementary file 5 — Supplementary Information 5 A dataset of crystal violet staining experiment including raw images, software code and analysis results. [file 41598_2019_44167_MOESM5_ESM.zip › images/d07_v_1.jpg.1.png]

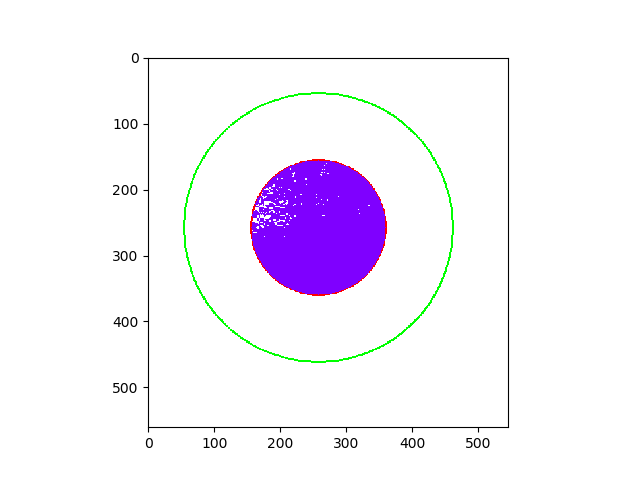

Supplement: Supplementary file 5 — Supplementary Information 5 A dataset of crystal violet staining experiment including raw images, software code and analysis results. [file 41598_2019_44167_MOESM5_ESM.zip › images/d07_v_1.jpg.2.png]

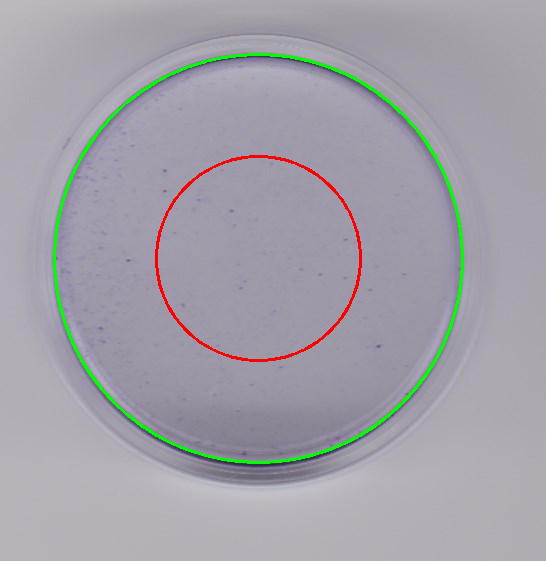

Supplement: Supplementary file 5 — Supplementary Information 5 A dataset of crystal violet staining experiment including raw images, software code and analysis results. [file 41598_2019_44167_MOESM5_ESM.zip › images/d07_v_1.jpg.tiff]
